# Rapid Asymmetric Transfer Hydroformylation (ATHF) of Disubstituted Alkenes Using Paraformaldehyde as a Syngas Surrogate

**DOI:** 10.1002/chem.201502049

**Published:** 2015-06-25

**Authors:** José A Fuentes, Rachael Pittaway, Matthew L Clarke

**Affiliations:** [a] School of Chemistry, University of St Andrews EaStCHEM, St Andrews, Fife, KY16 9ST (UK), Fax: (+44)1334-463808 E-mail: mc28@st-andrews.ac.uk

**Keywords:** carbonylation, enantioselectivity, formaldehyde, hydroformylation, microwave chemistry

## Abstract

As an alternative to conventional asymmetric hydroformylation (AHF), asymmetric transfer hydroformylation (ATHF) by using formaldehyde as a surrogate for syngas is reported. A catalyst derived from commercially available [Rh(acac)(CO)_2_] (acac=acetylacetonate) and 1,2-bis[(2*S*,5*S*)-2,5-diphenylphospholano]ethane(1,5-cyclooctadiene) (Ph-BPE) stands out in terms of both activity and enantioselectivity. Remarkably, not only are high selectivities achievable, the reactions are very simple to perform, and higher enantioselectivity (up to 96 % *ee*) and/or turnover frequencies than those achievable by using the same catalyst (or other leading catalysts) can be obtained by using typical conditions for AHF.

The development of catalytic reactions that make use of surrogates for carbon monoxide is currently experiencing intense research activity.[1] Many carbonylation reactions, such as asymmetric hydroformylation (AHF) are efficient, atom economic, produce very little waste and make use of a very cheap and available feedstock, CO. In the best case, almost perfect chemo-, regio- and enantioselectivities are possible.[2]

However, in pharmacy research and organic synthesis, the use of pressurised CO can require initial investment in infrastructure, thus creating a barrier to its wider use. In addition, although specialist companies use CO to produce millions of tonnes of chemicals with a low *E* factor,[2] some possible commercial applications might be compromised by a lack of a suitable reactor, or perceived safety concerns regarding the transport or usage of CO gas. Therefore, these issues provide the impetus for research on catalysis with CO surrogates. The potential impact of a CO surrogate is significantly larger if its use does not involve the introduction of any expensive stoichiometric reagents and the surrogate is cheap; relatively few papers demonstrate these features.[1b,c] On this basis, the transfer hydroformylation of alkenes using formaldehyde is a potentially ideal transformation,[3] but had not been reported in an enantioselective fashion.[3c] Herein, we show that asymmetric transfer hydroformylation (ATHF) can, in fact, be carried out on low-reactivity alkenes, giving products in minutes, often with high enantioselectivity. Moreover, the enantiomeric purities of the aldehydes and/or TONs/TOFs exceed those observed in the conventional hydroformylation using syngas.

At the onset of this work, we had found that some typical easy substrates for hydroformylation, such as mono-substituted vinyl arenes, did not seem suited to ATHF, because we obtained the linear isomer as the major product. This can be explained by the low pressures and high temperatures; conditions at which the regioselectivity is very sensitive to changes in pressure, as was recently explained by the Landis group.[4a] Therefore, we did not pursue this initial step towards the development of ATHF further. We postulated that quite poorly reactive substrates, such as 1,2-difunctionalised alkenes, might actually be a better choice of alkene for ATHF, because the temperatures required for the formation of aldehydes would be nicely balanced with those needed for decarbonylation, there will be less CO available to inhibit alkene coordination, and the aldehydes might be more stable to racemisation. Conventional AHF of such substrates is still a challenging task (Scheme 1).

**Scheme 1:**
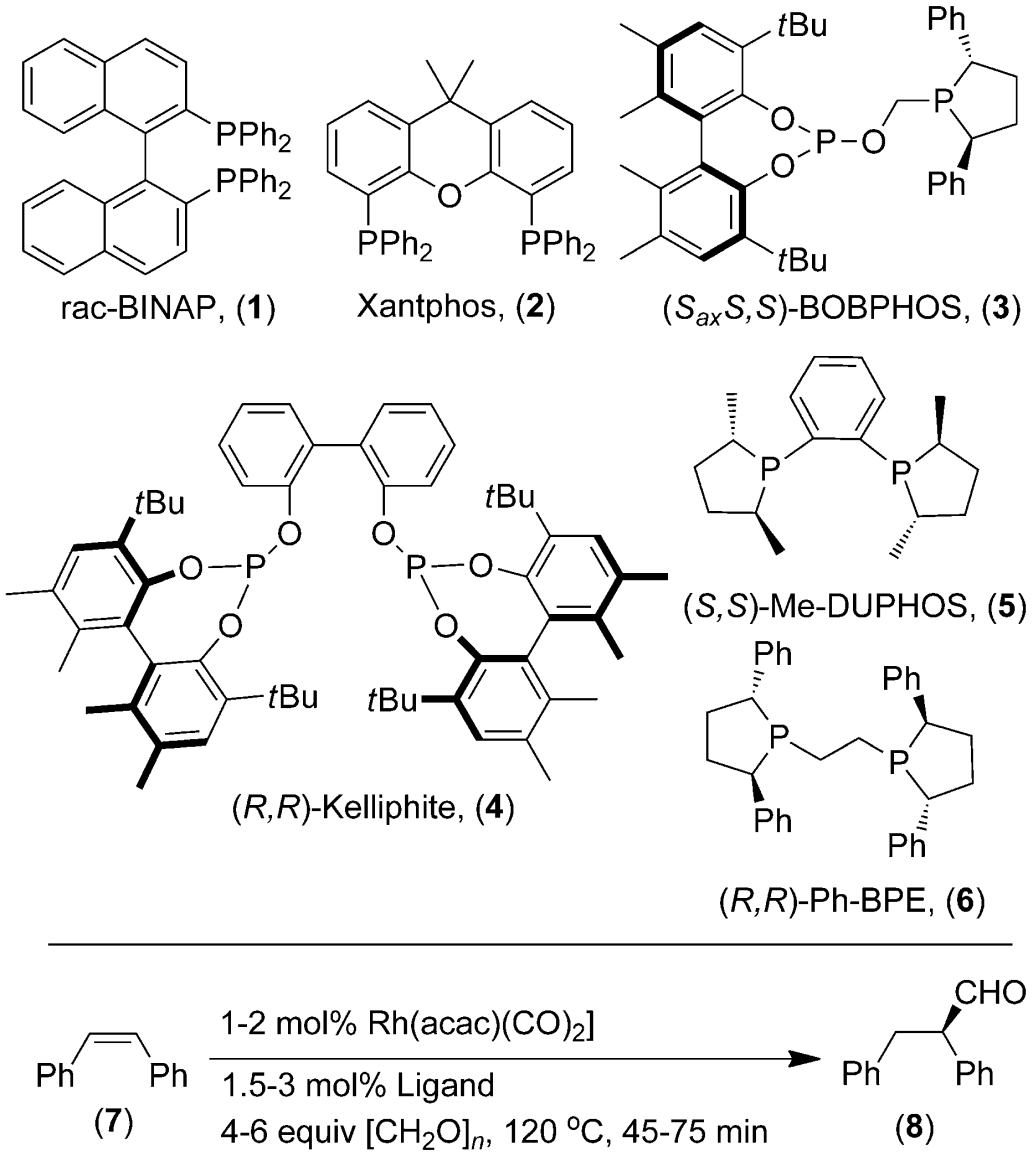
Asymmetric transfer hydroformylation of *cis*-stilbene using a range of hydroformylation catalysts.

A variety of ligands were screened in the asymmetric transfer hydroformylation of *cis*-stilbene (Table 1). Although ligands that give very reactive conventional enantioselective hydroformylation catalysts, such as Kelliphite[5a] and BOBPHOS,[5b] gave very low yields, *(R,R*)-Ph-BPE/[Rh(acac)(CO)_2_],[5c] stands out for ATHF. During the preparation of this manuscript, Morimoto and co-workers disclosed the use of [RhCl(COD)]_2_/(*R*,*R*)-Ph-BPE as the catalyst for transfer hydroformylation of mono-substituted vinyl arenes, with only slightly lower regioselectivity than in conventional AHF, and with 67–95 % *ee* after 10–40 h at 80 °C.[3c] The [Rh(acac)(CO)_2_]/Ph-BPE catalyst shows very useful scope for transfer hydroformylation: notable in the context of the hydroformylation reaction, which is often very sensitive to alkene structure. In the case of the *cis*-stilbene hydroformylation, we found that [RhCl(COD)]_2_ and Ph-BPE, as was used in Reference 3 c, was not an effective catalyst (2 % conversion), although a significant amount of gas pressure built up (Table 1, entry 10). The use of a combination of [RhCl(COD)]_2_ and [Rh(acac)(CO)_2_] was also studied, but did not hold any advantages over using [Rh(acac)(CO)_2_] alone (Table 2, entry 3). These reactions can be carried out using commercially available disposable microwave vessels with crimp caps, either using an oil bath or microwave heating, or in commercially available, very basic, low-pressure glass or steel reactors (Table 2, entries 1–4). Good results were also achieved at 0.2 mol % Rh loading (Table 2, entry 5).

**Table 1:** Asymmetric transfer hydroformylation of *cis*-stilbene (7) using a range of hydroformylation catalysts

Entry^[a]^	Ligand [mol %]	Conversion [%]^[b]^	Product,8[%]^[b]^	*ee* [%]^[c]^
1	**1** [3]	4	0	n.d.
2	**2** [3]	12	0	n.d.
3	**3** [3]	83	8	55 (*S*)
4	**4** [3]	48	0	n.d.
5	PPh_3_ [6]	25	0	n.d.
6	dppe [3]	23	0	n.d.
7	dcype [3]	2	1	n.d.
8	**5** [3]	22	4	n.d.
9	**6** [3]	88	80 [72]	95 (*R*)
10^[d]^	**6** [3]	2	2	n.d.
11^[d]^	**1** [1.5]+**2** [1.5]	34	0	n.d.

[a] Standard conditions according to Scheme 1 using 6 equiv [CH_2_O]_*n*_ in toluene and using 2 mol % [Rh(acac)(CO)_2_] at 120 °C for 45 min. [b] Conversion of *cis*-stilbene, and % aldehyde **8** determined by ^1^H NMR against cyclooctane as internal standard. The remaining mass balance is *trans*-stilbene. Isolated yield in square brackets is for the primary alcohol formed by NaBH_4_ reduction and after chromatography. [c] Measured on the primary alcohol by chiral HPLC (see the Supporting Information). [d] [Rh(μ**-**Cl)(COD)]_2_ (1 mol %). dppe=1,2-Bis(diphenylphosphino)ethane; dcype=1,2-bis(dicyclohexylphosphino); n.d.=not determined.

**Table 2:** Asymmetric transfer hydroformylation of *cis*-stilbene and substituted stilbene derivatives

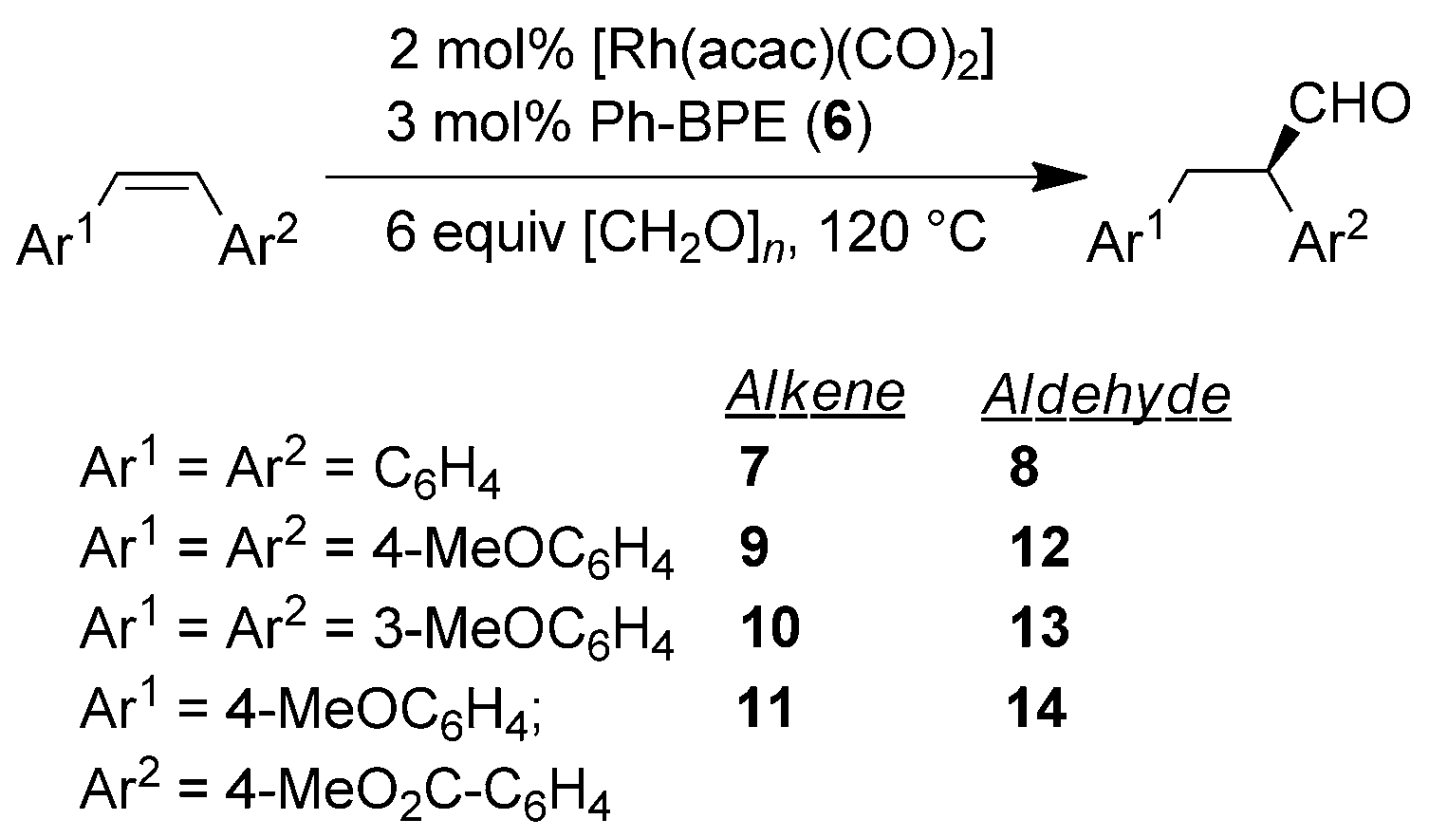					
Entry^[a]^	Alkene	Ligand [mol %]	Conversion [%]^[b]^	Product [%]^[b]^	*ee* [%]^[c]^
1	**7**	(*R,R*)-**6** [3]	88	80 [72]	95 (*R*)
2^[d]^	**7**	(*R,R*)-**6** [1.5]	92	82	95 (*R*)
3^[e]^	**7**	(*R,R*)-**6** [1.5]	79	71	94 (*R*)
4^[d]^	**7**	(*R,R*)-**6** [1.5]	85	77	95 (*R*)
5^[f]^	**7**	(*S,S*)-**6** [0.3]	84	59	93 (*S*)
6	**9**	(*R,R*)-**6** [3]	68	67 [58]	96 (−)
7	**10**	(*R,R*)-**6** [3]	99	88 [82]	92 (−)
8^[g]^	**11**	(*R,R*)-**6** [3]	85	61[43]^[g]^	84 (94)

[a] Standard conditions: 6 equiv [CH_2_O]_*n*_ in toluene using 2 mol % [Rh(acac)(CO)_2_] and 3 mol % of (*R*,*R*)-PhBPE (**6**) at 120 °C for 60 min. [b] Conversion of alkene, and % aldehyde determined by ^1^H NMR against cyclooctane as internal standard [isolated yield is for the primary alcohol formed after NaBH_4_ reduction and chromatography in brackets]. [c] Measured on the primary alcohol by chiral HPLC (see the Supporting Information). [d] [Rh(acac)(CO)_2_] (1 mol %), [CH_2_O]_*n*_ (4 equiv) 75 min. The reaction in entry 4 utilised a 50 mL glass pressure vessel at 110 °C for 180 min. [e] [Rh(acac)(CO)_2_] (0.5 mol %) and [RhCl(COD)]_2_ (0.25 mol %), 75 min. [f] [Rh(acac)(CO)_2_] (0.2 mol %), 240 min. [g] Reaction time=30 min. Aldehyde **14** was formed as 68:32 mix of regioisomers. The major isomer is drawn (*ee* in brackets is for minor isomer).

The conventional AHF of *cis*-stilbene using Rh/Ph-BPE catalysts has not been reported; in fact, as is documented in the Supported Information, we found that this substrate is very sluggish under conditions that give very high rates and *ee* for AHF of terminal alkenes. The best results we were able to achieve in AHF was 74 % *ee* at 69 % conversion after 20 hours using a S/C of 250 (average TOF ca. 9 h^−1^). The results obtained herein in ATHF with an average TOF of around 65–95 h^−1^ with high *ee* are quite an improvement, irrespective of the convenience of using formaldehyde. In fact, these appear to be the highest *ee* and TOF reported in any AHF of this substrate.[4b] We do not present this as evidence of a different mechanism, but suggest that the conditions for hydroformylation generated by using a surrogate at a high temperature for a short time are beneficial in these reactions. It does not appear that the same combination of TOF and *ee* are achievable easily in conventional AHF; this could be connected to alkene coordination in the conventional hydroformylation being inhibited by CO pressure, and possibly the effect of different pressures, reaction times and temperatures on various pathways for erosion of enantioselectivity.

With these results in hand, we synthesised several other *cis*-stilbene derivatives and examined them in the ATHF reaction. The alkenes can be readily synthesised by Sonogashira coupling, followed by *Z*-selective partial reduction, as described in the Supporting Information. It was pleasing to find that these substrates also gave high enantioselectivity (Table 2, entries 6–8). The non-symmetric substrate **11** gave slightly lower chemoselectivity due to the enhanced isomerisation to the *trans*-alkene; *trans*-stilbene derivatives do not react under these ATHF (or conventional AHF conditions) using Rh/PhBPE catalysts.

There has been some interest in the conventional hydroformylation of pyrroline derivatives since β-proline is a useful building block,[4d, 6] so this type of substrate was examined. Using CBz-3-pyrroline, essentially no reaction was observed; this substrate was also found to poison the ATHF of *cis*-stilbene (see the Supporting Information). We suggest that at these very low pressures, a stable chelate complex is formed. In the case of the ATHF of *N*-tosyl 3-pyrroline, we observed a minor side-product arising from isomerisation and subsequent hydroformylation.[4d, 6] Seeking to eliminate this, we investigated starting the reactions at high temperature to generate syngas, then stirring at a significantly lower temperature while hydroformylation took place. Although this was a slight improvement in terms of *ee*, the yield did not vary regardless of how much the temperature was lowered in the second stage (see the Supporting Information). Subsequently, we discovered that this substrate undergoes ATHF in only five minutes (Table 3, entry 3).

**Table 3:** Rapid transfer hydroformylation of a selection cyclic alkenes

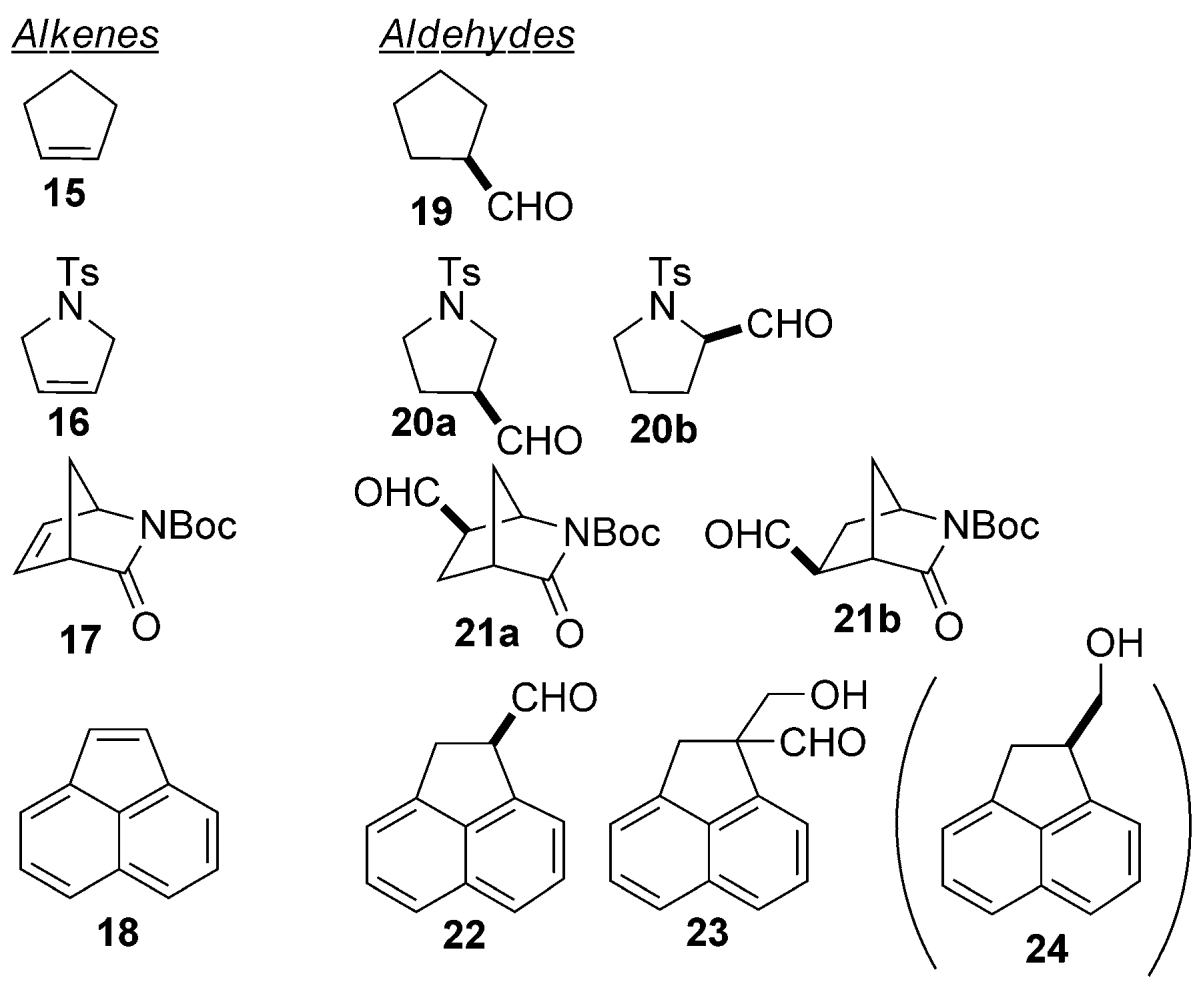					
Entry^[a]^	Alkene	*t* [min]	Conversion [%]^[b]^	Product [%]^[b]^	*ee* [%]^[c]^
1^[d]^	**15**	10	91	88	n.a.
2^[e]^	**15**	25	97	90 [44]	n.a.
3	**16**	5	>99	**20 a**, 70	78
				**20 b**, 5	74
4^[f]^	**16**	40	>99	**20 a**, 82 [72]	77
				**20 b**, 8 [5]	70
5^[g]^	**17**	5	>99	**21 a**, 62	n.a.
				**21 b**, 25	n.a.
6	**18**	5	64	**22** [31]	43
				**23** [26]	n.a.
7	**18**	10	84	n.d. [45 % of **24**]	46
				n.d. [26 % of **23 a**]	n.a.

[a] Standard conditions: 6 equiv [CH_2_O]_*n*_ in toluene using 2 mol % [Rh(acac)(CO)_2_] and 3 mol % of (*R*,*R*)-PhBPE (**6**) at 120 °C. [b] Conversion of alkene, and % aldehyde determined by ^1^H NMR against cyclooctane as internal standard [isolated yield for the primary alcohol formed after NaBH_4_ reduction and chromatography]. [c] Determined by using the corresponding primary alcohol using chiral HPLC (see the Supporting Information). [e] [Rh(acac)(CO)_2_] (1.0 mol %), PhBPE (1.5 mol %). Isolated yield obtained for the carboxylic acid formed by NaClO_2_/NaH_2_PO_4_/TEMPO oxidation. [f] [Rh(acac)(CO)_2_] (0.5 mol %), (*R*,*R*)-PhBPE (0.75 mol %). [g] (*S*,*S*)-PhBPE (**6**) was used. Isolated yield (69 %) of a 29:71 mixture of aldehydes **21 a**/**b** after chromatography; n.a.=not applicable.

The reactivity of [Rh(acac)(CO)_2_]/Ph-BPE in transfer hydroformylation appears much higher than previous transfer hydroformylation catalysts.[3] Therefore, this catalyst is also useful for achiral transformations. For example, cyclopentene is readily converted to aldehyde **19** in ten minutes (Table 3, entry 1). The catalyst-controlled diastereoselective hydroformylation of *N*-Boc lactam **17** is also of interest, because the products can lead to a variety of building blocks for carbocyclic nucleoside analogues.[7] This substrate was also found to be completely converted in a five-minutes reaction time (Table 3, entry 5). Hydroformylation of acenaphthylene has been studied previously only in an achiral sense, although the products are potentially interesting building blocks for biologically active compounds.[8] Surprisingly, we observed a new aldehyde side-product (Table 3, entries 6 and 7). This was proven to formed from an uncatalysed aldol reaction between aldehyde **22** and formaldehyde (Scheme 2). Aldehyde **22** presumably has a strong tendency to exist as its conjugated enol form. The enol form is expected to be the reactive species in the aldol reaction. The enolisation could be expected to preclude asymmetric hydroformylation, and under conditions that would give high *ee* for vinyl arenes, product of only 17 % *ee* was isolated. The ATHF does give higher *ee* (Table 3, entry 7), and is also complete in five minutes, but we have never observed higher enantioselectivity.

**Scheme 2:**
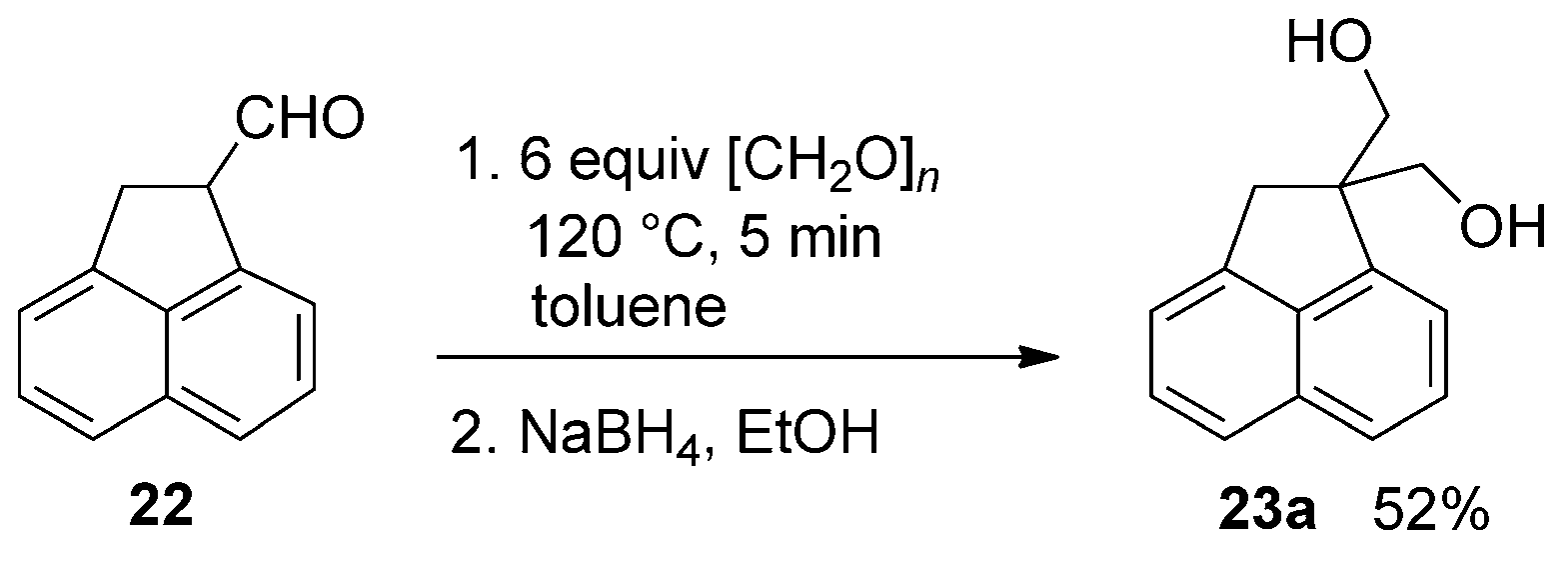
Aldol condensation of aldehyde **22** with formaldehyde under the same conditions used in ATHF.

It is possible that ATHF operates by either a decarbonylation/hydroformylation process, or some form of hydroacylation-type mechanism. We have measured the pressure inside the microwave vessels and noted that pressure builds up at an early stage of the reaction and then gradually falls off as the reaction proceeds. This is consistent with the formaldehyde being decomposed to CO and hydrogen. We have carried out a deuteroformylation of *cis*-stilbene using commercially available [CD_2_O]_*n*_. There is a lot of interest in preparing labelled aldehydes as intermediates, but the nature of conventional AHF is that very expensive labelled gases are often wasted when conducting AHF at pressure. This (unoptimised) deuteroformylation process is cheap and straightforward, so it could be synthetically useful, although we focused on using the labelling to shed light on the mechanism. In a reaction that went to 91 % conversion of starting material, inspection of the ^2^H NMR reveals that the product mixture is composed of deuterated aldehydes and deuterated *trans*-stilbene in a 5.7:1 ratio. This is consistent with the intermediacy of a Rh–alkyl species, as would have been observed in hydroformylation. The ^1^H NMR data revealed that, as a consequence of β-hydride elimination from Rh–alkyl species producing the unreactive deuterated *trans*-alkene, the majority of the aldehydes formed have the ^1^H isotope in the α-position (ca. 73 % of aldehydes detected). If this deuteroformylation reaction is done in the presence of 1.5 bar H_2_ gas, a greater proportion of the aldehydes produced have the ^1^H isotope at the formyl and β positions. This is consistent with a mechanism involving the reaction with either D_2_ or H_2_ gas, rather than the intermediacy of a Rh–formyl species. Further discussion and spectra are given in the Supporting Information.

In the seminal work on linear selective transfer hydroformylation using formaldehyde, several species were detected by ^31^P{^1^H} NMR data.[3a] Heating [Rh(acac)(CO)_2_] and Ph-BPE at 120 °C with an excess of [CH_2_O]_*n*_ (24 equiv) in toluene for five minutes gave a single Rh species, as was detected by ^31^P{^1^H} and ^1^H NMR spectroscopies. This can be assigned as trigonal bypyramidal equatorial-axial [Rh(H)(Ph-BPE)(CO)_2_], which has previously been detected by Vogt and Cornelissen under conventional hydroformylation conditions (Scheme 3).[9]

**Scheme 3:**
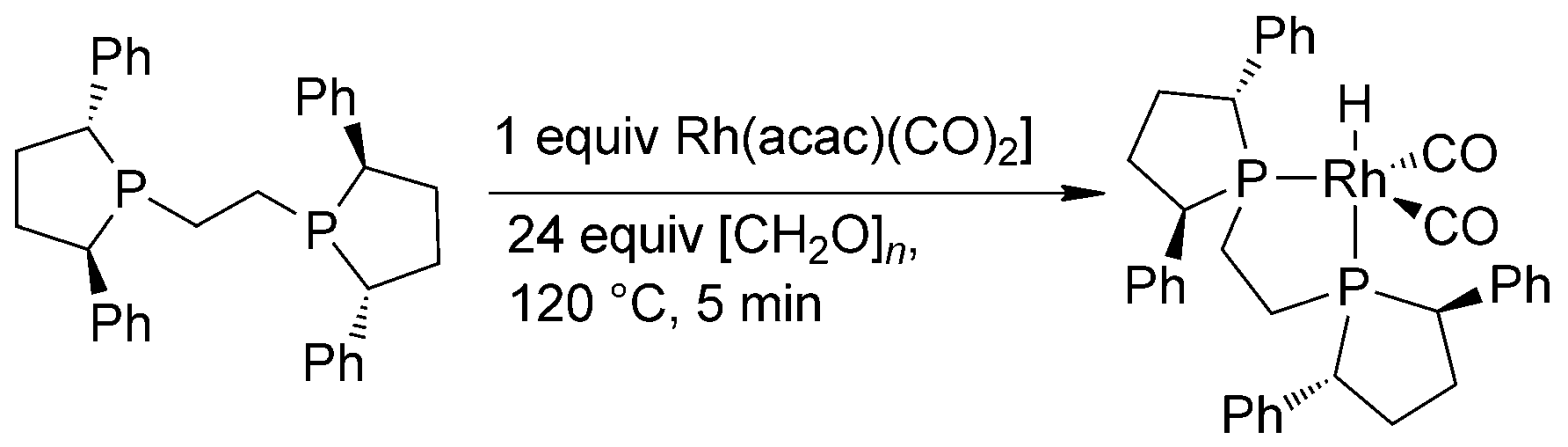
Only the same single active species was detected as in conventional hydroformylation using the Ph-BPE ligand.

In our view, this species is clinching evidence for a tandem decarbonylation/hydroformylation pathway for these reactions. Given that [IrH_3_(P P)(CO)] complexes have been isolated and been found to eliminate dihydrogen,[10] we would suggest that the analogous Rh species could be an intermediate in the decarbonylation stage. This could be formed after C–H activation, reductive elimination of acacH, carbonyl migration and loss of CO, followed by a second cycle of formaldehyde decarbonylation. A [RhH_3_(P P)(CO)] complex could lose hydrogen to directly give the hydroformylation catalyst, [Rh(H)(Ph-BPE)(CO)_*n*_].

In summary, ATHF has been demonstrated to be an easily carried out reaction; it uses commercially available components, gives better results on some less activated substrates than those easily obtained using conventional hydroformylation conditions. The mechanism is a decarbonylation/hydroformylation pathway. This reaction should become a valuable part of the synthetic chemist toolbox.

## Experimental Section

A Biotage 5 mL microwave vial containing a stirring bar was charged with [Rh(acac)(CO_2_)] (2 mol %, 4.3 mg, 0.0166 mmol), (*R,R*)-Ph-BPE (3 mol %, 12.6 mg, 0.0249 mmol) and paraformaldehyde (6 equiv, 150 mg). The vial was sealed with a crimp cap, purged with three vacuum/argon cycles and left under an argon atmosphere. Alkene (150 mg, 0.832 mmol), toluene (3 mL) and an internal standard (1 drop of cyclooctane) were added to a Schlenk flask under an inert atmosphere. The resulting solution was mixed, and a small sample was taken for a *t*_0_ NMR analysis. The solution was then added to the microwave vial and heated to 120 °C using microwave radiation. After 45 min, the vial was cooled, and the positive pressure inside the vial was released by piercing the cap with a needle. A small sample was taken and analysed by ^1^H NMR spectroscopy to calculate the conversion to the resulting aldehyde. The aldehyde was reduced using NaBH_4_ to the corresponding alcohol (see the Supporting Information) and isolated using flash chromatography on silica gel (*n*-hexane/EtOAc 4:1) to give 2,3-diphenylpropan-1-ol as a white solid (126 mg, 0.60 mmol). The enantiomeric excess of the alcohol was determined by chiral HPLC on a Chiralcel OD-H, 250×4.6 mm, *n*-hexane/2-propanol 90:10, 0.5 mL min^−1^, 254 nm, *t*_R_[(−)-(*R*), major]=18.3 min, *t*_R_[(+)-(*S*), minor]=20.5 min; [*α*]_D_^20^ −85.5 (*c* 1.0, CHCl_3_, *ee* 95 %; lit: [*α*]_D_^30^ −80.7 (*c* 1.13, CHCl_3_), *ee* 93 %) See the Supporting Information for NMR data.

**CAUTION!!!** This procedure gives enhanced convenience and requires far less complicated safety measures than conventional AHF, because the syngas is added to the reactor in solid form. Using the amounts above, the highest pressure detected during an ATHF using 6 equiv of [CH_2_O]_*n*_ was 7 bar at reaction temperature (4 bar at RT). This is well inside the 20 bar limit of the microwave vessels. The highest pressure detected at 120 °C when no ATHF was taking place, but decarbonylation did was 13 bar. In the 50 mL glass or steel pressure vessels, the highest pressure detected was 3 bar. Similar to the majority of chemical reactions with volatile components, scale-up of these procedures needs careful consideration of the pressures possible and the maximum operating pressure of the vessel used.

## Supporting Information

As a service to our authors and readers, this journal provides supporting information supplied by the authors. Such materials are peer reviewed and may be re-organized for online delivery, but are not copy-edited or typeset. Technical support issues arising from supporting information (other than missing files) should be addressed to the authors.

miscellaneous_information
