# Supplementary material for: Rapid Asymmetric Transfer Hydroformylation (ATHF) of Disubstituted Alkenes Using Paraformaldehyde as a Syngas Surrogate
Source: Chemistry. 2015 Jun 25;21(30):10645–9. doi: 10.1002/chem.201502049 (PMC4539593; doi:10.1002/chem.201502049)
Supplement: Supplementary file 1 — miscellaneous_information [file chem0021-10645-sd1.pdf]

CHEMISTRY
A European Journal

Supporting Information

Rapid Asymmetric Transfer Hydroformylation (ATHF) of Disubstituted Alkenes Using Paraformaldehyde as a Syngas Surrogate

José A. Fuentes, Rachael Pittaway, and Matthew L. Clarke*[a]

chem_201502049_sm_miscellaneous_information.pdf

Table of contents

1. General information

2. Preparation of substrates for hydroformylation

2.1 Synthesis of *cis*-alkenes. General methods

3. Hydroformylations. General procedures

3.1.General procedure for rhodium catalysed hydroformylation using phenylphosphatrioxa-adamantane. Preparation of racemic samples.

3.2 General procedure for reduction of aldehyde products to alcohols

3.3. Conventional Hydroformylation (AHF) using Argonaut Endeavour (AE) parallel autoclave.

3.4. Asymmetric hydroformylation of acenaphthylene (18) using a low pressure autoclave

3.5. General procedure for the asymmetric transfer hydroformylation of alkenes.

3.6. General procedure for the asymmetric transfer hydroformylation of alkenes using a low pressure autoclave ATHF

4. ATHF of (–)-(1*R*,4*S*)-*tert*-butyl 3-oxo-2-azabicyclo[2.2.1]hept-5-ene-2-carboxylate (17)

5. ATHF of pyrroline derivatives and cyclopentene

6. Analytical data of selected compounds

7. Deuterioformylation Experiments

8. Formation of [RhH(CO)$_2$Ph-BPE)] under ATHF conditions. Coordination studies.

9. NMR spectra of selected compounds

10. HPLC chromatograms for racemic alcohols and catalysis products

11. References

1. General Information

Dry toluene was obtained from an Innovative Technologies Puresolve 400 solvent still. Other solvents were bought and used as received without further purification. ^{Me}CgPPh was prepared according to the literature.[1] Chiral ligands were donated by Chirotech Technology Centre, Dr Reddys or obtained form Aldrich. All manipulations were carried out under an inert atmosphere of nitrogen or argon unless otherwise stated. Syngas was obtained from BOC. Microwave reactions were carried out in a Biotage® Initiator using 10 ml heavy-walled reactor vials (5 ml maximum loading) equipped with an air tight seal (crimp cap). Solvents were removed by rotary evaporation on a Heidolph labrota 4000. Flash column chromatography was performed on Davisil silica gel Fluorochem 60 Å, particle size 35-70 μm. HPLC analysis was determined on a Varian Prostar operated by Galaxie workstation software. NMR spectra were recorded on Bruker Avance 300, 400 and 500 instruments. Proton chemical shifts are referenced to internal residual solvent protons. Proton signal multiplicities are given as s (singlet), d (doublet), t (triplet), q (quartet), m (multiplet), br (broad) or a combination of the above. When appropriate, coupling constants (*J*) are quoted in Hz and are reported to the nearest 0.1 Hz. All spectra were recorded at room temperature unless otherwise stated and the solvent for a particular spectrum is given in parentheses. Carbon chemical shifts are referenced to the carbon signal of the deuterated solvents. Chemical ionisation mass spectroscopy and electron ionisation mass spectroscopy were performed on a Micromass GCT spectrometer. Electrospray mass spectroscopy was performed on a Micromass LCT spectrometer. All were operated by Mrs Caroline Horsburgh at St Andrews University, or at the ESPSRC National Mass Spectrometry Service Centre, Swansea University, using Waters ZQ4000, Thermofisher LTQ Orbitrap XL and Finnigan MAT 900 XLT Instruments. Only major peaks are reported, and intensities are quoted as percentages of the base peaks. Optical rotations were measured on a Perkin elmer 341 polarimeter using a 1 ml cell with a 1 dm path length at 20 °C using the sodium D-line.

2. Preparation of Substrates for hydroformylation

4-(4-methoxyphenylethynyl)benzoic acid methyl ester.[2] General method.

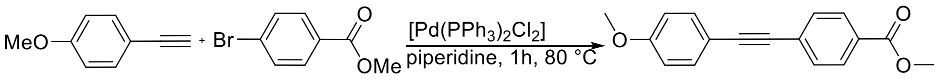

An oven-dried Schlenk was charged with 4-methoxyphenylacetylene (0.8 mL, 6.1 mmol), 4-methylbromobenzoate (1.04 g, 6.1 mmol), [$PdCl_2(PPh_3)_2$] (0.17 g, 0.24 mmol, 2 mol%) and piperidine (1.5 mL) and placed under an argon atmosphere. The reaction was heated to 80 °C for 1 hour and then cooled to room temperature. The obtained solid mixture was then diluted with toluene (30 mL) and the organic layer was extracted with water (1 x 30 mL) and 1M HCl (1 x 15 mL). The organic layer was then washed further with water (2 x 30 mL) before being dried over $MgSO_4$, filtered and concentrated using a rotary evaporator. The crude product was then purified by trituration with methanol (30 mL) to obtain 4-(4-methoxyphenylethynyl)benzoic acid methyl ester an orange solid (1.23 g, 76 %). ^{1}H NMR (500 MHz, $CDCl_3$) δ 8.02 (2H, d, *J* = 8.5 Hz, ArCH), 7.57 (2H, d, *J* = 8.5 Hz, ArCH), 7.48 (2H, d, *J* = 9.1 Hz, 2H, ArCH), 6.88 (2H, d, *J* = 9.3 Hz, 2H, ArCH), 3.92 (3H, s, OCH_3), 3.84 (3H, s, OCH_3). ^{13}C NMR (126 MHz, $CDCl_3$) δ 167.1 (C=O), 160.5 (ArC), 133.8 (ArCH), 131.8 (ArCH), 130.0 (ArCH), 129.6 (ArC), 128.9 (ArC), 115.2 (ArC), 114.6 (ArCH), 93.0 (CC), 88.0 (CC), 55.8 (OCH_3), 52.7 (OCH_3). HRMS $(ES)^+$: 289.0828 $[M+Na]^+$, $C_{17}H_{14}NaO_3^+$ requires 289.0835.

1,2-bis(3-methoxyphenyl)ethyne[3]

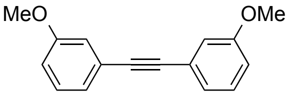

Purified by chromatography on SiO_2 using Hexane/EtOAc 6:1 as eluent to give the alkyne as a pale yellow solid (0.898 g, 3.77 mmol, 50%). ^{1}H NMR (500 MHz, $CDCl_3$) δ 7.30-7.26 (2H, m, ArCH), 7.18-7.16 (2H, m, ArCH), 7.10 (2H, dd, *J* = 2.5, 1.4 Hz, ArCH), 6.93 (2H, ddd, *J* = 8.3, 2.6, 0.9 Hz, ArCH), 3.86 (6H, s, OCH_3); ^{13}C NMR (126 MHz, $CDCl_3$) δ 159.5 (ArC), 129.6 (2xArCH), 124.3 (2xArCH), 124.3 (ArC), 116.4 (2xArCH), 115.2 (2xArCH), 89.2 (CC), 55.4 ($2xOCH_3$).

1,2-bis(4-methoxyphenyl)ethyne[4]

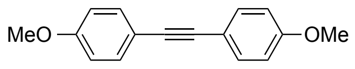

Purified by chromatography on SiO_2 using Hexane/EtOAc 8:1 as eluent to give the alkyne as a white solid (0.442 g, 56%). ^1H NMR (500 MHz, $CDCl_3$) δ 7.45 (4H, d, *J* = 8.8 Hz, ArCH), 6.87 (4H, d, *J* = 8.8 Hz, ArCH), 3.82 (6H, s, OCH_3); ^{13}C NMR (126 MHz, $CDCl_3$) δ 159.5 (ArC), 133.0 (4xArCH), 115.8 (ArC), 114.1 (4xArCH), 88.1 (CC), 55.4 (2xOCH_3).

2.1 Synthesis of cis-alkenes. General methods

Preparation of (*Z*)-methyl-4-(4-methoxystyryl)benzoate. Method A

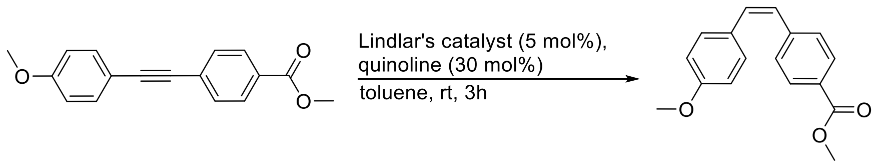

A 50 mL two-necked round-bottom flask was charged with 4-(4-methoxyphenylethynyl)benzoic acid methyl ester (407 mg, 1.35 mmol), Lindlar's catalyst (162.5 mg, 0.077 mmol, 5 mol%), quinoline (59.4 mg, 0.46 mmol, 30 mol%) and anhydrous toluene (11.5 mL) under a N_2 atmosphere. The flask was the purged with H_2 using a H_2 filled balloon before being stirred at room temperature for 3 hours under a positive pressure of H_2 (H_2 filled balloon). The reaction mixture was then filtered through celite to remove the catalyst. The celite was washed with diethyl ether. The combined organic layer were extracted using water and then brine before drying over Na_2SO_4, filtering and concentrating *in vacuo*. The crude product was purified via column chromatography (SiO_2, hexane: ethyl acetate 9:1) to remove the quinoline before triturating with methanol (30 mL) to remove any unreacted starting material to afford (*Z*)-methyl-4-(4-methoxystyryl)benzoate as a yellow oil (81 %). ^1H NMR (500 MHz, $CDCl_3$) δ 7.90 (2H, d, *J* = 8.3, ArH), 7.34 (2H, d, *J* = 8.3, ArH), 7.15 (2H, d, *J* = 9.1, ArH), 6.77 (2H, d, *J* = 8.8, ArH), 6.62 (1H, d, *J* = 11.9 Hz, HC=CH), 6.52 (1H, d, *J* = 12.2 Hz, HC=CH), 3.90 (3H, s, OCH_3), 3.78 (3H, s, OCH_3). ^{13}C NMR (126 MHz, $CDCl_3$) δ 166.8 (C=O) 158.8 (ArC), 142.4 (ArC), 131.6 (C=C), 130.0 (ArCH), 129.4 (Ar-CH), 128.9 (ArC), 128.6 (ArCH), 128.2 (ArC), 127.5 (C=C), 113.5 (ArCH), 55.0 (OCH_3), 51.8 (OCH_3); HRMS $(ES)^+$: 291.0985 $[M+Na]^+$, $C_{17}H_{16}NaO_3^+$ requires 291.0992.

Method B

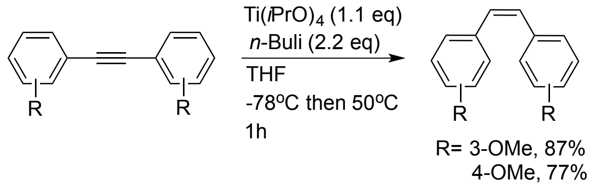

A modification of a known procedure was followed.[5] A Schlenk tube was charged with the diarylalkyne, 1,2-bis(3-methoxyphenyl)ethyne, (1 equiv, 2.94 mmol, 0.7 g) under a N_2 atmosphere. THF was added (10 mL) and the Schlenk was cooled down to -78 ºC. $Ti(O^iPr)_4$ (1.1 equiv, 3.24 mmol, 0.96 mL) was added and then *n*-BuLi (2.2 equiv, 6.47 mmol, 1.6 M in hexanes, 4.04 mL) was added dropwise. The resulting solution was left to reach room temperature for 15 min, warmed to 50 ºC and left for 1 hour. The reaction was quenched by adding saturated aqueous NH_4Cl solution (5 mL) and diluted with ether (10 mL). The resulting mixture was stirred at room temperature for 10 minutes and then separated. The aqueous solution was extracted with Et_2O (3 x 15 mL). The organic phases were combined and dried over magnesium sulfate. After removal of the solvent under reduced pressure, the resulting crude product was purified by flash chromatography on silica gel to give pure *cis*-olefin.

(*Z*)-1,2-bis(3-methoxyphenyl)ethene[6]

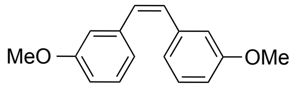

Purified by chromatography on SiO_2 using Hexane/Et_2O 10:1 as eluent to give the alkene as a colourless oil (0.613 g, 2.55 mmol, 87%). ^1H NMR (500 MHz, $CDCl_3$) δ 7.17-7.14 (2H, m, ArCH), 6.87-6.85 (2H, m, ArCH), 6.82-6.81 (2H, m, ArCH), 6.76-6.74 (2H, m, ArCH), 6.59 (2H, s, HC=CH), 3.67 (6H, s, OCH_3); ^{13}C NMR (126 MHz, $CDCl_3$) δ 159.5 (2xArC), 138.7 (2xArC), 130.5 (2xArCH), 129.3 (2xArCH), 121.6 (HC=CH), 113.9 (2xArCH), 113.4 (2xArCH), 55.2 (2xOCH_3); HRMS $(ES)^+$: 241.1216 $[M+H]^+$, $C_{16}H_{17}O_2$ requires 241.1223.

(*Z*)-1,2-bis(4-methoxyphenyl)ethene[7]

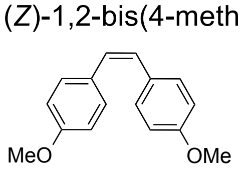

Purified by chromatography on SiO_2 using Hexane/Et_2O 10:1 as eluent to give the alkene as a colourless oil (0.282 g, 1.18 mmol, 77%). ^1H NMR (400 MHz, $CDCl_3$) δ 7.21 (4H, d, *J* = 8.9 Hz, ArCH), 6.78 (4H, d, *J* = 8.9 Hz, ArCH), 6.46 (2H, s, HC=CH), 3.80 (6H, s, OCH_3); ^{13}C NMR

(101 MHz, $CDCl_3$) δ 158.6 (2xArC), 130.2 (4xArCH), 130.1 (2xArC), 128.5 (HC=CH), 113.7 (4xArCH), 55.3 (2xOCH_3).

3. Hydroformylations. General procedures

3.1. General procedure for rhodium catalysed hydroformylation using phenylphosphatrioxa-adamantane. Preparation of racemic samples.

All reactions were run using a Parr 50 mL stainless steel autoclave equipped with a pressure gage, gas inlet, safety valve and injection port equipped with rubber septum. [Rh(acac)(CO_2)] (0.4 mol%) and ^{Me}CgPPh (2 mol%) were placed into a glass vial. A stirring bar was added and the vial was sealed with a crimp cap and put under inert atmosphere. Two needles were pierced into the vial and this was introduced into the autoclave, which had been previously purged with three vacuum/argon cycles. Toluene (3 mL), an internal standard (approximately 50 μl of 1-methylnaphthalene or 1 drop of cyclooctane) and the alkene (1 mmol) were added using a syringe. The autoclave was then purged three times with CO/H_2 (1:1), pressurised to 20 bar and immersed into an oil bath preheated to the desired temperature (80-100 °C). After the desired reaction time (2.5-4 h), the autoclave was cooled down to room temperature, the pressure slowly released and opened. A small sample was taken and analysed by ^{1}H NMR to calculate the conversion of the resulting aldehydes. Products were reduced to the corresponding alcohols and isolated using flash chromatography if required to develop HPLC methods for the measuring of the enantiomeric excess.

3.2. General procedure for reduction of aldehyde products to alcohols

The crude reaction mixture was diluted with EtOH (3 ml). $NaBH_4$ (111 mg, 3 mmol) was added and the reaction mixture was stirred at room temperature under a N_2 atmosphere for 3 h. The contents of the vial were then transferred to a round bottom flask and the solvents partially removed under vacuum. The reaction mixture was then diluted with dichloromethane (5 ml), quenched with dilute HCl (1M) until it reached acidic pH, and transferred to a separation funnel. The organic layer was separated and the aqueous layer extracted 3 times with dichloromethane (3 x 10 ml). The combined organic layers were dried over anhydrous $MgSO_4$ and the solvent was removed with a rotary evaporator to give the crude mixture, which was purified by chromatography on SiO_2 with unoptimised methods. In all cases, this primarily yielded samples of pure alcohols.

Table 1. Hydroformylation of alkenes using Rh / ^{Me}CgPPh (phenylphosphatrioxa-adamantane) catalyst and analytical methods for ee determination.

Entry[a]	Alkene	P. (bar)	T (°C)	t (h)	Conv (%)[b]	Conv (%)[c]	Analytical method for e.e. determination[d,e]
1	**7**	20	120	2	95	41	Chiralcel OD-H, 90:10 hexane: 2-propanol, 0.5 ml/min.
2	**10**	20	90	3	30	22	Chiralcel OD-H, 80:20 hexane: 2-propanol, 0.5 ml/min.
3	**9**	20	100	4	43	31	Chiralcel OD-H, 90:10 hexane: 2-propanol, 0.5 ml/min.
4	**16-cbz**	25	100	2.5	>99	**20a** 70	Chiralpak AD-H, 95:5 *n*-hexane: 2-propanol, 1.0 ml/min.
						**20b** 30	Chiralpak AD-H, 95:5 *n*-hexane: 2-propanol, 1.0 ml/min.
5	**16-Ts**	20	80	3	98	**20a** 94	Chiralcel OD-H, 95:5 hexane: 2-propanol, 1.0 ml/min.
						**20b** 2	Chiralpak AD-H, 90:10 *n*-hexane: 2-propanol, 1.0 ml/min.
6	**11**	20	80	72	>99	95 (57:43)	Chiralcel OD-H, 95:5 hexane: 2-propanol, 0.5 ml/min.
7	**22**	20	100	3	>99	95	Chiralpak AD-H, 95:5 *n*-hexane: 2-propanol, 0.5 ml/min.

[a] Reactions performed using 1 mmol of substrate, 0.4 % [Rh(acac)(CO)$_2$] and 2 % (^{Me}CgPPh) in toluene (3 ml) at 80-120 °C for 2.5-4 h at syngas pressure specified. [b] % alkene consumed determined by ^{1}H NMR using cyclooctane or 1-methylnaphthalene as internal standard. [c] % aldehyde determined by ^{1}H NMR using cyclooctane or 1-methylnaphthalene as internal standard. [d] All products were reduced to racemic alcohols using NaBH$_4$. [e] Analytical methods developed by chiral HPLC using the corresponding alcohols.

3.3. Conventional Hydroformylation (AHF) using Argonaut Endeavour (AE) parallel autoclave system.

Conventional Hydroformylation (AHF) using Argonaut Endeavour (AE) parallel autoclave system reveals *cis*-stilbene to be sluggish using normally highly reactive catalysts. Prior to carrying out the reaction the vessels of the AE were purged, initially with nitrogen. The required amount of [Rh(acac)(CO)$_2$] and each ligand (L:Rh= 1.25:1), as a solution in dry toluene, was injected into each well of the AE (typically 2mL of solution added). The mixture was then placed under 10 bar CO:H$_2$ (1:1) and heated to 50 °C for ~ 40 min in order to 'pre-activate' the catalyst prior to substrate addition. The pressure was then vented and the apparatus allowed to cool to room temperature. *Cis*-stilbene was then added (3 mmol) as a solution in toluene doped with cyclooctane internal standard (an NMR had been taken of this solution to calibrate the conversion measured by NMR later). The total volume of liquid in each well was 5 mL. The apparatus was then purged three times with CO:H$_2$ (1:1) , placed under the required pressure and heated to the required reaction temperature for the required time. The crude reaction mixtures were then analysed by ^{1}H NMR spectroscopy with the enantiomeric excess recorded on the resulting primary alcohols obtained after NaBH$_4$ reduction by HPLC as described elsewhere in this ESI.

Enantioselective Hydroformylation of *cis*-stilbene

Entry[a]	Ligand (mol%)	T (°C)	Time (h)	P (bar)	Conv. %	e.e.[b] %
1	PPh_3 (2%)	70	18	10	26.6	n.d.
2	(*S,S,S*)-BOBPHOS (0.8%)	70	18	3	88.7	60
3	(*S,S,S*)-BOBPHOS (0.8%)	70	20	15	53.5	62
5	(*R,R*)-Ph-BPE (0.5%)	75	20	3	68.7	74
6	(*R,R*)-Ph-BPE (0.5%)	75	20	15	27.5	84
7	(*R,R*)-Kelliphite (0.5%)	75	20	3	23.2	36

[a] Reaction carried out in Argonaut using 0.4 mol% |Rh(acac)$(CO)_2$] and x mol % ligand that was pre-activated at 10 bar syngas, 50 °C, 40 minutes in 2 ml toluene, prior to the addition of substrate in 3 ml toluene. % conversion determined by NMR on crude sample with no side products detected. [b] e.e. determined after reduction of the crude reaction mixtures to alcohols using $NaBH_4$ and using chiral HPLC.

3.4. Asymmetric hydroformylation of acenaphthylene (18) using a low pressure autoclave

The reactions was run using a Parr 50 mL stainless steel autoclave equipped with a pressure gage, gas inlet, safety valve and injection port equipped with rubber septum. [Rh(acac)(CO_2)] (0.5 mol%) and (*S,S*)-PhBPE (0.75 mol%) were placed into a glass vial. A stirring bar was added and the vial was sealed with a crimp cap and put under inert atmosphere. Two needles were pierced into the vial and this was introduced into the autoclave, which had been previously purged with three vacuum/argon cycles. Toluene (3 mL), an internal standard (1 drop of cyclooctane) and acenaphthylene (126 mg, 0.832 mmol) were added using a syringe. The autoclave was then purged three times with CO/H_2 (1:1), pressurised to 5 bar and immersed into an oil bath preheated to the desired temperature (60 °C). After the desired reaction time (17 h), the autoclave was cooled down to room temperature, the pressure slowly released and opened. A small sample was taken and analysed by ^1H NMR to calculate the conversion of the resulting aldehyde (59% conversion of alkene, 56 % conversion to aldehyde). The aldehyde was reduced to the corresponding alcohol in order to measure the corresponding enantiomeric excess (17 % ee).

3.5. General procedure for the asymmetric transfer hydroformylation of alkenes

A Biotage 5 ml microwave vial (see general information) containing a stirring bar was charged with [Rh(acac)$(CO)_2$] (2 mol%, 4.3 mg, 0.0166 mmol), (*R,R*)-Ph-BPE (3 mol%, 12.6 mg, 0.0249 mmol) and paraformaldehyde (6 equiv, 150 mg). The vial was sealed with a crimp cap, purged with three vacuum/argon cycles and left under a nitrogen atmosphere. Alkene (0.832 mmol), toluene (3 mL) and an internal standard (approximately 50 µl of 1-methylnaphthalene

or 1 drop of cyclooctane) were added to a Schlenk flask under an inert atmosphere. The resulting solution was mixed and a small sample taken for a t_0 NMR. The solution was then added to the microwave vial and heated to the desired temperature (80-120 °C). using microwave radiation. After the desired reaction time (5 min-4.5 h), the vial was cooled down and the positive pressure inside the vial was released by piercing the cap with a needle. A small sample was taken and analysed by ^{1}H NMR to calculate the conversion of the resulting aldehydes. Products were reduced using the general procedure to the corresponding alcohols and isolated using flash chromatography. The crude product of reduction reaction afforded the same ee as pure samples of the corresponding alcohols in all cases.

3.6. General procedure for the asymmetric transfer hydroformylation of alkenes using a low pressure autoclave ATHF

A glass vial containing a stirring bar was charged with [Rh(acac)$(CO)_2$] (2 mol%, 4.3 mg, 0.0166 mmol), (*R,R*)-Ph-BPE (3 mol%, 12.6 mg, 0.0249 mmol) and paraformaldehyde (6 equiv, 150 mg). The vial was sealed with a crimp cap, purged with three vacuum/argon cycles, placed inside the autoclave and left under a nitrogen atmosphere. Alkene (0.832 mmol), toluene (3 mL) and an internal standard (approximately 50 μl of 1-methylnaphthalene or 1 drop of cyclooctane) were added to a Schlenk flask under an inert atmosphere. The resulting solution was mixed and a small sample taken for a t_0 NMR. The solution was then added to the microwave vial and heated to the desired temperature in a preheated oil bath. After the desired reaction time, the autoclave was cooled to room temperature and the pressure was released. A small sample was taken and analysed by ^{1}H NMR to calculate the conversion of the resulting aldehydes. Products were reduced using the general procedure to the corresponding alcohols.

4. ATHF of (–)-(1*R*,4*S*)-*tert*-butyl 3-oxo-2-azabicyclo[2.2.1]hept-5-ene-2-carboxylate (17)

(–)-**17** was synthesised *via* a literature procedure[12].

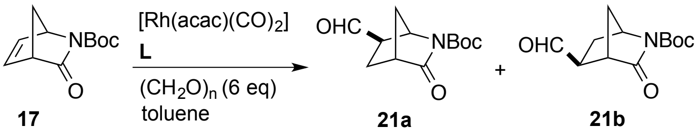

Entry	[Rh(acac)(CO)$_2$] %	[Rh(cod)Cl]$_2$ %	L %	T °C	t min	Conv.[a] %	Conv[b] 21a %	Conv[b] 21b %
1	2.0	-	(*R,R*)-Ph-BPE, 3	110	75	>99	41	40
2	2.0	-	(*R,R*)-Ph-BPE, 3	120	5	>99	42	42
3	1.0	0.5	(*R,R*)-Ph-BPE, 1.5 (*R,R*)-Kelliphite, 1.5	100	60	>99	56	37
4[d]	1.0	0.5	(*R,R*)-Ph-BPE, 1.5 (*R,R*)-Kelliphite, 1.5	100	60	>99	53	35
5	2.0	-	(*R,R*)-Kelliphite, 3	100	60	14	0	0.5
6[c]	2.0	-	(*S,S*)-Ph-BPE, 3	100	5	>99	25	62

[a] Conversion of (–)-**13** measured by ^{1}H NMR against 1-methylnaphthalene as internal standard. [b] Conversion to aldehydes measured by ^{1}H NMR against 1-methylnaphthalene as internal standard. [c] 69% isolated yield obtained as a mixture of aldehydes after filtration through a plug of neutral alumina using toluene as solvent. [d] [Rh(cod)Cl]$_2$ and (*R,R*)-Kelliphite were stirred in toluene in a Schlenk flask for 30 min at room temperature and then were added to the microwave vial.

5. ATHF of pyrroline derivatives and cyclopentene

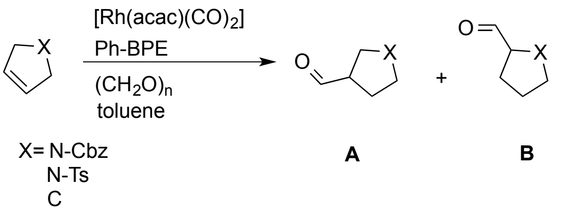

Ent[a]	X	[Rh(acac)(CO)$_2$] %	[Rh(cod)Cl]$_2$ %	L %	(CH$_2$O)n eq	T °C	t min	Conv.[b] %	Conv[c] %	ee[d] %
1	N-Cbz	0.5	0.25	1.5	4	120	90'	59	0	-
2	N-Cbz	0.5	0.25	1.5	4	140	90'	99	0	-
3[g]	N-Cbz + *cis*-Stilb	0.5	0.25	1.5	4	120	75'	28	0	-
4	N-Ts	2.0	-	3	6	100	90'	>99	**A**: 76, **B**:16	68, 81
5[e]	N-Ts	2.0	-	3	6	120, 70	5',2h	>99	**A**: 69, **B**:7	76, 80
6[e]	N-Ts	2.0	-	3	6	120, 60	5',4h	>99	**A**: 66, **B**:7	76, 80
7[e]	N-Ts	2.0	-	3	6	120, 40	5',17h	>99	**A**: 65, **B**:6	78, 78
8	N-Ts	2.0	-	3	6	120	5'	>99	**A**: 70, **B**:5	78, 74
9[f]	N-Ts	0.5	-	0.75	6	120	40'	>99	**A**: 82[72], **B**:8[5]	77, 70
10	C	2.0	-	3	6	120	10'	91	**A:** 88	-
11	C	1.0	-	1.5	6	120	25'	97	**A:** 90[44]	-

[a] Reactions were carried out following the general procedure for the asymmetric transfer hydroformylation of alkenes described in the ESI. [b] Conversion of alkene by ^{1}H NMR against 1-methylnaphthalene or cyclooctane as internal standard. [c] Conversion to aldehydes measured by ^{1}H NMR against 1-methylnaphthalene or cyclooctane as internal standard. [d] ee measured by chiral HPLC on the corresponding alcohols, first number correspond at the alcohol derived from A and the second number to the alcohol derived form B [e] The reaction mixture was heated at 120 ºC for 5 min using microwave radiation and then heated at the indicated T on the table using an oil bath for the remaining time. [f] [Isolated yield]. [g] Reaction performed using a mixture of *cis*-stilbene and N-Cbz protected substrate 8:2.

6. Analytical data of selected compounds

2,3-diphenylpropan-1-ol[8]

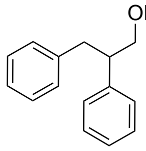

Prepared according to general procedure described in section 3.2. Purified by chromatography on SiO_2 using Hexane/EtOAc 4:1 as eluent to give the alcohol as a white solid (126 mg, 0.60 mmol, 72%). $[\alpha]_D^{20}$ –85.5 (*c* 1.0, $CHCl_3$, ee 95%) {lit[8]: $[\alpha]_D^{30}$ –80.7 (*c* 1.13, $CHCl_3$), ee 93%}. ^1H NMR (500 MHz, $CDCl_3$) δ 7.36-7.33 (2H, m, ArCH), 7.28-7.23 (4H, m, ArCH), 7.21-7.18 (2H, m, ArCH), 7.14-7.12 (2H, m, ArCH), 3.85-3.78 (2H, m, $HOCH_2$), 3.16-3.10 (1H, m, CH), 3.08-3.04 (1H, m, CH_2), 2.95 (1H, dd, *J* 13.5, 7.4 Hz, CH_2), 1.38 (1H, br t, *J* 5.2 Hz, OH); ^{13}C NMR (126 MHz, $CDCl_3$) δ 142.0 (ArC), 140.0 (ArC), 129.2 (2xArCH), 128.8 (2xArCH), 128.4 (2xArCH), 128.2 (2xArCH), 127.0 (ArCH), 126.2 (ArCH), 66.5 ($HOCH_2$), 50.3 (CH), 38.8 (CH_2); HRMS $(ES)^+$: 235.1091 $[M+Na]^+$, $C_{15}H_{16}ONa$ requires 235.1093. The enantiomeric excess of the alcohol was determined by HPLC on a Chiralcel OD-H, 250 x 4.6 mm, 90:10 *n*-hexane: 2-propanol, 0.5 ml/min, 254 nm, t_R[(–)-(*R*), major] = 18.3 min, t_R[(+)-(*S*), minor] = 20.5 min.

2,3-bis(3-methoxyphenyl)propan-1-ol

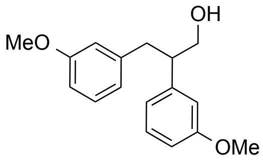

Prepared according to general procedure described in section 3.2. Purified by chromatography on SiO_2 using Hexane/EtOAc 1:1 as eluent to give the alcohol as a white solid (189 mg, 0.69 mmol, 82%). $[\alpha]_D^{20}$ –66.0 (*c* 1.0, $CHCl_3$, ee 92%); ^1H NMR (400 MHz, $CDCl_3$) δ 7.28-7.15 (2H, m, ArCH), 6.85-6.72 (5H, m, ArCH), 6.67-6.66 (1H, m, ArCH), 3.83-3.76 (2H, m, $HOCH_2$), 3.81 (3H, s, OCH_3), 3.76 (3H, s, OCH_3), 3.13-3.06 (1H, m, CH), 3.03-2.88 (2H, m, CH_2), 1.34 (1H, br s, OH); ^{13}C NMR (101 MHz, $CDCl_3$) δ 159.9 (ArC), 159.6 (ArC), 143.7 (ArC), 141.6 (ArC), 129.8 (ArCH), 129.4 (ArCH), 121.6 (ArCH), 120.5 (ArCH), 114.8 (ArCH), 114.2 (ArCH), 112.1 (ArCH), 111.7 (ArCH), 66.5 ($HOCH_2$), 55.3 (OCH_3), 55.2 (OCH_3), 50.2 (CH), 38.8 (CH_2); HRMS $(ES)^+$: 295.1297 $[M+Na]^+$, $C_{17}H_{20}O_3Na$ requires 295.1305. The enantiomeric excess of the alcohol was determined by HPLC on a Chiralcel OD-H, 250 x 4.6 mm, 80:20 *n*-hexane: 2-propanol, 0.5 ml/min, 254 nm, t_R[(–)-(*R*), major] = 18.4 min, t_R[(+)-(*S*), minor] = 22.4 min.

2,3-bis(4-methoxyphenyl)propan-1-ol[9]

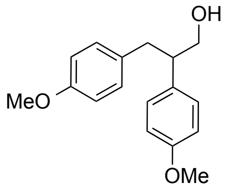

Prepared according to general procedure described in section 3.2. Purified by chromatography on SiO_2 using Hexane/EtOAc 1:1 as eluent to give the alcohol as a white solid (131 mg, 0.48 mmol, 58%). $[\alpha]_D^{20}$ –93.6 (*c* 1.0, $CHCl_3$, ee 96%); ^1H NMR (300 MHz, $CDCl_3$) δ 7.11 (2H, d, *J* = 8.7 Hz, ArCH), 6.99 (2H, d, *J* = 8.7 Hz, ArCH), 6.85 (2H, d, *J* = 8.7 Hz, ArCH), 6.76 (2H, d, *J* = 8.7 Hz, ArCH), 3.83-3.70 (2H, m, $HOCH_2$), 3.79 (3H, s, OCH_3), 3.76 (3H, s, OCH_3), 3.05-2.86 (2H, m, CH, CH_2), 2.81 (1H, dd, *J* 13.2, 7.4 Hz, CH_2), 1.31 (1H, br s, OH); ^{13}C NMR (75 MHz, $CDCl_3$) δ 158.5 (ArC), 158.0 (ArC), 133.9 (ArC), 132.1 (ArC), 130.1 (2xArCH), 129.2 (2xArCH), 114.2 (2xArCH), 113.8 (2xArCH), 114.8 (ArCH), 114.2 (ArCH), 112.1 (ArCH), 111.7 (ArCH), 66.6 ($HOCH_2$), 55.4 (OCH_3), 55.3 (OCH_3), 49.7 (CH), 38.1 (CH_2); HRMS $(ES)^+$: 295.1293 $[M+Na]^+$, $C_{17}H_{20}NaO_3$ requires 295.1305. The enantiomeric excess of the alcohol was determined by HPLC on a Chiralcel OD-H, 250 x 4.6 mm, 90:10 *n*-hexane: 2-propanol, 0.5 ml/min, 254 nm, t_R[(+), minor] = 28.1 min, t_R[(–), major] = 31.4 min.

Methyl 4-(1-hydroxy-3-(4-methoxyphenyl)propan-2-yl)benzoate and methyl 4-(3-hydroxy-2-(4-methoxyphenyl)propyl)benzoate:

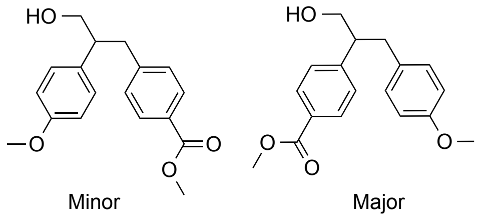

Prepared according to general procedure described in section 3.2. Purified via column chromatography (SiO_2, 9:1 hexane: ethyl acetate) to give the inseparable alcohols as an oily mixture (83 mg, 0.028 mmol, 43 % (combined yield) in a 68:32 ratio).

Minor isomer: methyl 4-(3-hydroxy-2-(4-methoxyphenyl)propyl)benzoate:

White oil, ^1H NMR (400 MHz, $CDCl_3$) δ 7.86 (2H, d, -C(O)OMeArH), 7.12 (2H, d, ArH), 7.05 (2H, d, ArH), 6.83 (2H, d, -OMeArH), 3.87 (3H, s, OCH_3), 3.74 - 3.81 (2H, m, $HOCH_2$), 3.77 (3H, s, OCH_3), 2.87-3.13 (1H, m, CH), 2.87-3.13 (1H, m, CH_2), 2.87-2.90 (1H, m, CH_2); ^{13}C NMR (101 MHz, $CDCl_3$) δ 166.9 (C=O), 158.3 (-OMeArC), 145.4 (ArC), 132.8 (ArC), 129.3 (2x-C(O)OMeArCH), 128.9 (2xArCH), 128.8 (2xArCH), 127.7 (-C(O)OMeArC), 113.8 (2x-OMeArCH), 66.3 ($HOCH_2$), 55.0 (CH_3-O), 51.8 (CH_3-O), 49.0 (CH), 38.6 (CH_2). HMBC ^1H- ^{13}C

NMR (400 MHz, $CDCl_3$) δ 7.12 - 38.6 (ArH – CH_2); HRMS $(ES)^+$: 323.1249 $[M+Na]^+$, $C_{18}H_{20}NaO_4^+$ requires 323.1254. The enantiomeric excess of the alcohol was determined by HPLC on a Chiralcel OD-H, 250 x 4.6 mm, 95:5 *n*-hexane: 2-propanol, 0.5 ml/min, 254 nm, t_R[minor] = 90.3 min, t_R[major] = 93.5 min.

Major isomer: methyl 4-(1-hydroxy-3-(4-methoxyphenyl)propan-2-yl)benzoate:

White oil, ^1H NMR (400 MHz, $CDCl_3$) δ 7.94 (2H, d –C(O)OMeArH), 7.25 (2H, d, ArH), 6.94 (2H, d, ArH), 6.75 (2H, d, -OMeArH), 3.89 (3H, s, OCH_3), 3.81 (2H, m, $HOCH_2$), 3.74 (3H, s, OCH_3), 2.98-3.13 (1H, m, CH), 2.98-3.13 (1H, m, CH_2), 2.80-2.85 (1H, m, CH_2); ^{13}C NMR (101 MHz, $CDCl_3$) δ 166.9 (C=O), 157.7 (-OMeArC), 147.5 (ArC), 131.2 (ArC), 129.7 (2x-C(O)OMeArCH), 129.6 (2xArCH), 128.4 (-C(O)OMeArC), 128.0 (2xArCH), 113.5 (2x-OMeArCH), 65.8 ($HOCH_2$), 55.0 (CH_3-O), 51.8 (CH_3-O), 50.3 (CH), 37.4 (CH_2). HMBC ^1H- ^{13}C NMR (400 MHz, $CDCl_3$) δ 6.94 – 37.4 (ArH – CH_2); HRMS $(ES)^+$: 323.1249 $[M+Na]^+$, $C_{18}H_{20}NaO_4^+$ requires 323.1254. The enantiomeric excess of the alcohol was determined by HPLC on a Chiralcel OD-H, 250 x 4.6 mm, 95:5 *n*-hexane: 2-propanol, 0.5 ml/min, 254 nm, t_R[minor] = 108 min, t_R[major] = 167 min.

Cyclopentanecarboxylic acid

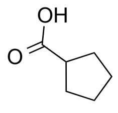

The aldehyde was oxidised by a modification of a literature procedure.[14] The reaction crude from the ATHF of cyclopentene (1.664 mmol) was diluted with *t*BuOH (3 ml) and TEMPO (10 mol%, 26 mg, 0.1664 mmol) was added to the mixture. In a separate flask NaH_2PO_4 (2 equiv., 0.400 g, 3.33 mmol) was dissolved in water (9 mL), to this solution was added $NaClO_2$ (2 equiv., 0.301 g, 3.33 mmol) and the mixture was stirred until a clear solution formed. This oxidant solution was added slowly to the crude aldehyde solution and the reaction mixture was stirred at room temperature for 2.5 h (^1H NMR of the reaction crude showed full consumption of aldehyde). Hexane (15 mL) was added to the flask and the mixture was vigorously stirred for 5 min. The organic phase was separated and the aqueous layer washed with toluene (6 mL). The combine organic layers were then washed with a solution of NaS_2O_3 (173 mg in 9 mL of water). The organic solution containing the carboxylic acid was next washed with a saturated solution of $NaHCO_3$ (3 x 10 mL) and the combined aqueous layers were washed with toluene (10 mL). The aqueous layer was then acidified with concentrated HCl until acidic pH and transferred to a separation funnel. The aqueous layer was extracted 3 times with dichloromethane (3 x 15 ml) and ethyl acetate (2 x 15 mL). The combined organic layers were dried over anhydrous $MgSO_4$ and the solvent was removed with a rotary evaporator to give

cyclopentanecarboxylic acid as a colourless oil (83 mg, 0.73 mmol, 44%). ^{1}H NMR (500 MHz, $CDCl_3$) δ 2.76 (1H, p, *J* = 7.8 Hz, CH), 1.95-1.88 (2H, m, CH_2), 1.87-1.79 (2H, m, CH_2), 1.76-1.68 (2H, m, CH_2), 1.63-1.55 (2H, m, CH_2); ^{13}C NMR (126 MHz, $CDCl_3$) δ 183.4 (COOH), 43.8 (CH), 30.61 (2xCH_2), 26.0 (2xCH_2); HRMS (ES)$^-$: 113.0603 [M]$^-$, $C_6H_9O_2$ requires 113.0608.

(1-tosylpyrrolidin-3-yl)methanol[10]

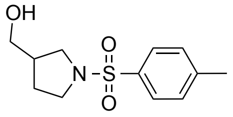

Prepared according to general procedure described in section 3.2. Purified by chromatography on SiO_2 using Hexane/EtOAc 1:2 as eluent to give the alcohol as a white solid (140 mg, 0.55 mmol, 66%). $[\alpha]_D^{20}$ +3.3 (*c* 1.0, $CHCl_3$, ee 63%); ^{1}H NMR (500 MHz, $CDCl_3$) δ 7.69 (2H, d, *J* = 8.2 Hz, ArCH), 7.31 (2H, d, *J* = 8.2 Hz, ArCH), 3.48-3.38 (2H, m, $HOCH_2$), 3.32-3.03 (4H, m, CH_2NCH_2), 2.42 (3H, s, CH_3), 2.33-2.25 (1H, m, CH), 2.00 (1H, br s, OH), 1.91-1.84 (1H, m, CH_2), 1.59-1.52 (1H, m, CH_2); ^{13}C NMR (126 MHz, $CDCl_3$) δ 143.6 (ArC), 133.3 (ArC), 129.8 (2xArCH), 127.7 (2xArCH), 64.1 ($HOCH_2$), 50.5 (CH_2), 47.5 (CH_2), 40.8 (CH), 27.6 (CH_2), 21.6 (CH_3); HRMS (ES)$^+$: 278.0815 [M+Na]$^+$, $C_{12}H_{17}NO_3SNa$ requires 278.0821. The enantiomeric excess of the alcohol was determined by HPLC on a Chiralcel OD-H, 250 x 4.6 mm, 95:5 *n*-hexane: 2-propanol, 1.0 ml/min, 254 nm, t_R[(–), minor] = 58.0 min, t_R[(+), major] = 61.1 min.

(1-tosylpyrrolidin-2-yl)methanol[11]

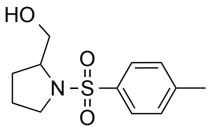

Prepared according to general procedure described in section 3.2. Purified by chromatography on SiO_2 using Hexane/EtOAc 1:2 as eluent to give the alcohol as a white solid (25 mg, 0.098 mmol, 12%). $[\alpha]_D^{20}$ –47.7 (*c* 1.0, $CHCl_3$, ee 76%) {lit[11]: $[\alpha]_D^{20}$ –62.3 (*c* 0.5, MeOH, ee 74%); ^{1}H NMR (500 MHz, $CDCl_3$) δ 7.73 (2H, d, *J* = 8.1 Hz, ArCH), 7.33 (2H, d, *J* = 8.1 Hz, ArCH), 3.70-3.60 (3H, m, C*H*, HOC*H*$_2$), 3.47-3.43 (1H, m, NCH_2), 3.27-3.23 (1H, m, NCH_2), 2.84 (1H, br s, OH), 2.43 (3H, s, CH_3), 1.82-1.40 (4H, m, CH_2CH_2); ^{13}C NMR (126 MHz, $CDCl_3$) δ 143.9 (ArC), 133.9 (ArC), 129.9 (2xArCH), 127.7 (2xArCH), 66.0 ($HOCH_2$), 62.0 (CH), 50.2 (CH_2), 29.0 (CH_2), 24.4 (CH_2), 21.7 (CH_3); HRMS (ES)$^+$: 278.0813 [M+Na]$^+$, $C_{12}H_{17}NO_3SNa$ requires 278.0821. The enantiomeric excess of the alcohol was determined by HPLC on a Chiralpak AD-H, 250 x 4.6 mm, 90:10 *n*-hexane: 2-propanol, 1.0 ml/min, 254 nm, t_R[(+)-(*R*), minor] = 27.8 min, t_R[(–)-(*S*), major] = 33.9 min.

(1*R*,4*R*,6*R*)-*tert*-butyl 6-formyl-3-oxo-2-azabicyclo[2.2.1]heptane-2-carboxylate (A) and (1*S*,4*S*,5*S*)-*tert*-butyl 5-formyl-3-oxo-2-azabicyclo[2.2.1]heptane-2-carboxylate (B)[12]

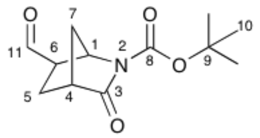

A

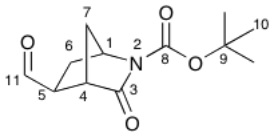

B

Analysis by ^{1}H NMR spectroscopy showed > 99% conversion to aldehydes, based on internal standard, regio-isomeric ratio **(A)**:**(B)** =1:2.4.

(1*R*,4*R*,6*R*)-*tert*-butyl 6-formyl-3-oxo-2-azabicyclo[2.2.1]heptane-2-carboxylate (A)
^{1}H NMR (300 MHz, $CDCl_3$) δ_H= 9.74 (s, 1H, C11H), 4.71 (s, 1H, C1H), 2.97-3.05 (m, 1H, C6H), 2.81-2.87 (m, 1H, C4H), 2.19-2.27 (m, 1H, C5H), 1.79-1.92 (m, 2H, C5H and C7H), 1.46 (s, 9H, $C10H_9$) 1.33-1.39 (dt, 1H, *J*= 10.5 1.3Hz C7H).
(1*S*,4*S*,5*S*)-*tert*-butyl 5-formyl-3-oxo-2-azabicyclo[2.2.1]heptane-2-carboxylate (B)
^{1}H NMR ($CDCl_3$) δ_H= 9.72 (s, 1H, C11H), 4.53 (t, 1H, *J*= 1.7, C1H), 3.08-3.12 (m, 1H, C4H), 2.90-2.98 (m, 1H, C5H), 2.17-2.30 (m, 1H, C6H), 1.82-1.97 (m, 2H, C6H and C7H), 1.45 (s, 9H, $C10H_9$), 1.31-1.39 (dt, 1H, *J*= 10.5, 1.4 Hz, C7H).

(1,2-dihydroacenaphthylen-1-yl)methanol[15]

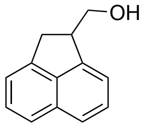

Prepared according to general procedure described in section 3.2. Purified by chromatography on SiO_2 using Hexane/EtOAc 1:1 as eluent to give the alcohol as a colourless oil (68 mg, 0.37 mmol, 45%). $[\alpha]_D^{20}$ –8.8 (*c* 1.0, $CHCl_3$, ee 46%) ^{1}H NMR (500 MHz, $CDCl_3$) δ 7.66 (1H, d, *J* 8.2, ArCH), 7.62 (1H, d, *J* 8.2, ArCH), 7.49-7.46 (2H, m, ArCH), 7.36 (1H, d, *J* 6.8, ArCH), 7.31 (1H, d, *J* 6.8, ArCH), 3.96-3.88 (3H, m, HOC*H*$_2$C*H*), 3.60-3.55 (1H, m, CH_2), 3.28-3.24 (1H, m, CH_2), 1.58 (1H, br s, OH); ^{13}C NMR (126 MHz, $CDCl_3$) δ 145.7 (ArC), 144.5 (ArC), 139.2 (ArC), 131.7 (ArC), 128.2 (ArCH), 127.8 (ArCH), 123.5 (ArCH), 122.6 (ArCH), 119.7 (ArCH), 119.5 (ArCH), 66.7 ($HOCH_2$), 46.2 (CH), 34.6 (CH_2); HRMS $(ES)^+$: 207.0775 $[M+Na]^+$, $C_{13}H_{12}ONa$ requires 207.0780. The enantiomeric excess of the alcohol was determined by HPLC on a Chiralpak AD-H, 250 x 4.6 mm, 95:5 *n*-hexane: 2-propanol, 0.5 ml/min, 254 nm, t_R[(–), major] = 21.7 min, t_R[(+), minor] = 23.5 min.

(1,2-dihydroacenaphthylene-1,1-diyl)dimethanol

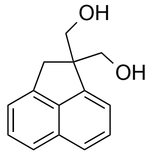

Prepared according to general procedure described in section 3.2. Purified by chromatography on SiO_2 using Hexane/EtOAc 1:1 as eluent to give the alcohol as a white solid (46 mg, 0.215 mmol, 26%). ^{1}H NMR (400 MHz, $CDCl_3$) δ 7.69 (1H, d, *J* 8.2, ArCH), 7.64 (1H, d, *J* 8.2, ArCH), 7.51-7.46 (2H, m, ArCH), 7.35-7.31 (2H, m, ArCH), 4.01 (2H, d, *J* 10.8, HOC*H*$_2$), 3.93 (2H, d, *J* 10.8, HOC*H*$_2$), 3.38 (2H, s, CH_2), 2.11 (2H, br s, OH); ^{13}C NMR (101 MHz, $CDCl_3$) δ 146.1 (ArC), 143.1 (ArC), 138.7 (ArC), 131.8 (ArC), 128.4 (ArCH), 127.8 (ArCH), 124.4 (ArCH), 122.8 (ArCH), 120.1 (ArCH), 119.6 (ArCH), 68.8 (2xHOCH_2), 55.1 (C), 38.4 (CH_2); HRMS $(ES)^+$: 237.0881 $[M+Na]^+$, $C_{14}H_{14}O_2Na$ requires 237.0886.

7. Deuterioformylation Experiments

Deuterioformylation of *cis*-stilbene using $[CD_2O]_n$ [98% D]

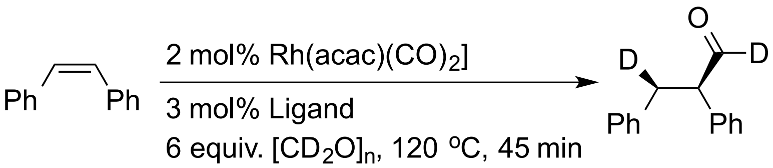

^{2}H NMR (toluene) of the crude mixture showing the product mixture of deuterated aldehydes and deuterated *trans*-stilbene in a 5.7:1 ratio.

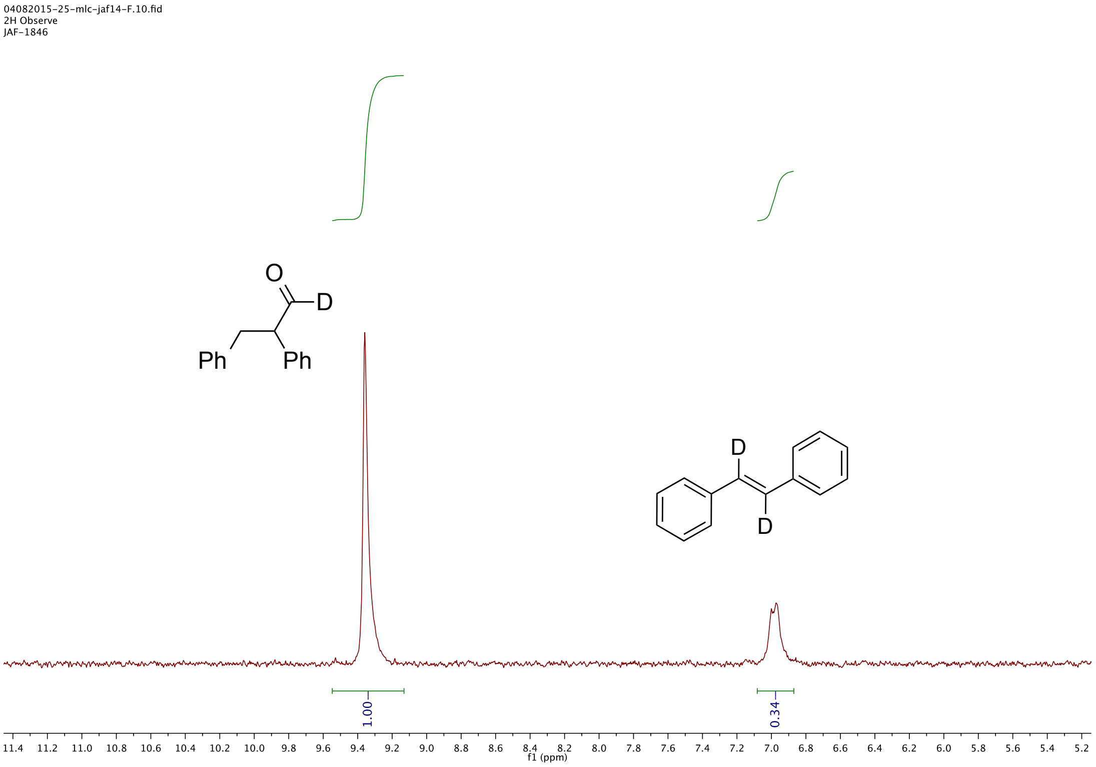

^{1}H-NMR ($CDCl_3$) of the crude mixture (above) and ^{1}H-NMR (C_6D_6) of *cis*-stilbene and cyclooctene (IS) in toluene (t_0 NMR) (below).

The ^{1}H-NMR and ^{2}H NMR reveals, assuming a product mixture of alkenes and aldehydes as observed in ATHF, the following amounts of products were observed:

Conversion of cis-stilbene: 90.5%

cis-stilbene left: 9.5%

^{1}H-aldehydes: 4%

^{2}H-aldehydes: 71.4%

^{2}H-*trans*-stilbene: 15.1 %

Aldehydes with ^{1}H in the alpha position: 55% conversion w.r.t. I.S. (73% of aldehydes)

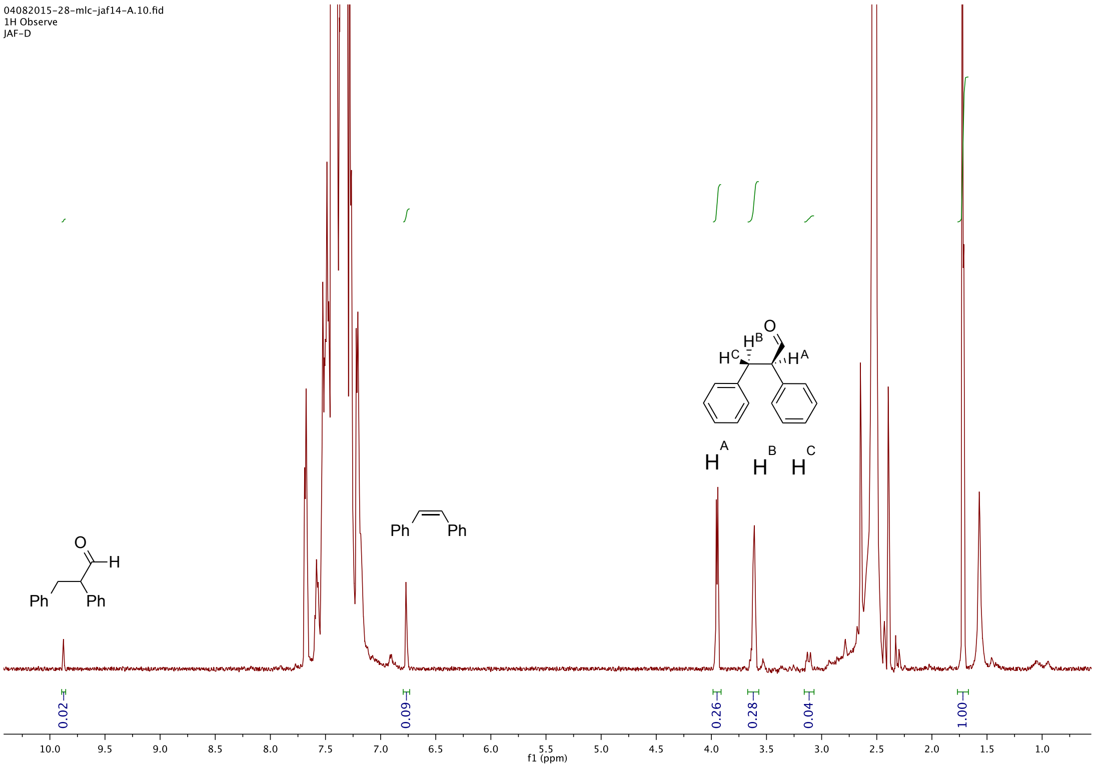
04082015-28-mlc-jaf14-A.10.fid
1H Observe
JAF-D
Ph
Ph
O
H
Ph
Ph
H^A
H^B
H^C
0.02
0.09
0.26
0.28
0.04
1.00
f1 (ppm)

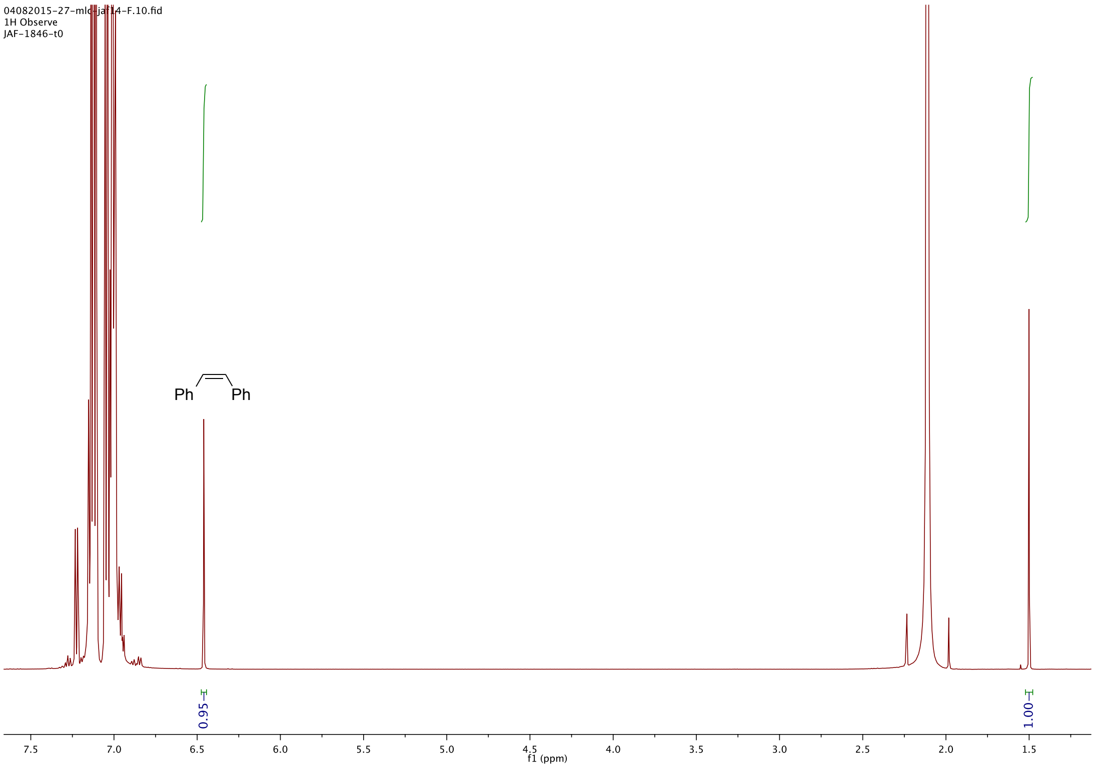
04082015-27-mlc-jaf14-F.10.fid
1H Observe
JAF-1846-t0
Ph
Ph
0.95
1.00
f1 (ppm)

Deuterioformylation of *cis*-stilbene using $[CD_2O]_n$ in the presence of H_2 gas

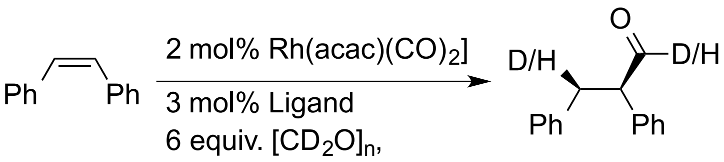

A Biotage 5 ml microwave vial containing a stirring bar was charged with $[Rh(acac)(CO)_2]$ (2 mol%, 4.3 mg, 0.0166 mmol), (*R,R*)-Ph-BPE (3 mol%, 12.6 mg, 0.0249 mmol) and paraformaldehyde (6 equiv, 150 mg). The vial was sealed with a crimp cap, purged with three vacuum/argon cycles and left under a nitrogen atmosphere. Toluene (2 mL) was added and the solution was heated to 120 °C using microwave radiation. After 5 min, the vial was cooled down and using a 20 mL syringe the solution was taken from the vial and injected in the autoclave containing a solution of *cis*-stilbene (0.832 mmol), toluene (2 mL) and an internal standard (approximately 50 µl of 1-methylnaphthalene). The autoclave was pressurised with 1.5 bar of H_2 and placed in a preheated oil bath at 120 °C. After 3 h, the autoclave was cooled to room temperature and the pressure was released. A sample was taken and analysed by ^{1}H NMR and ^{2}H NMR.

^{1}H-NMR (C_6D_6) of *cis*-stilbene and cyclooctene (IS) in toluene (t_0 NMR) (below).

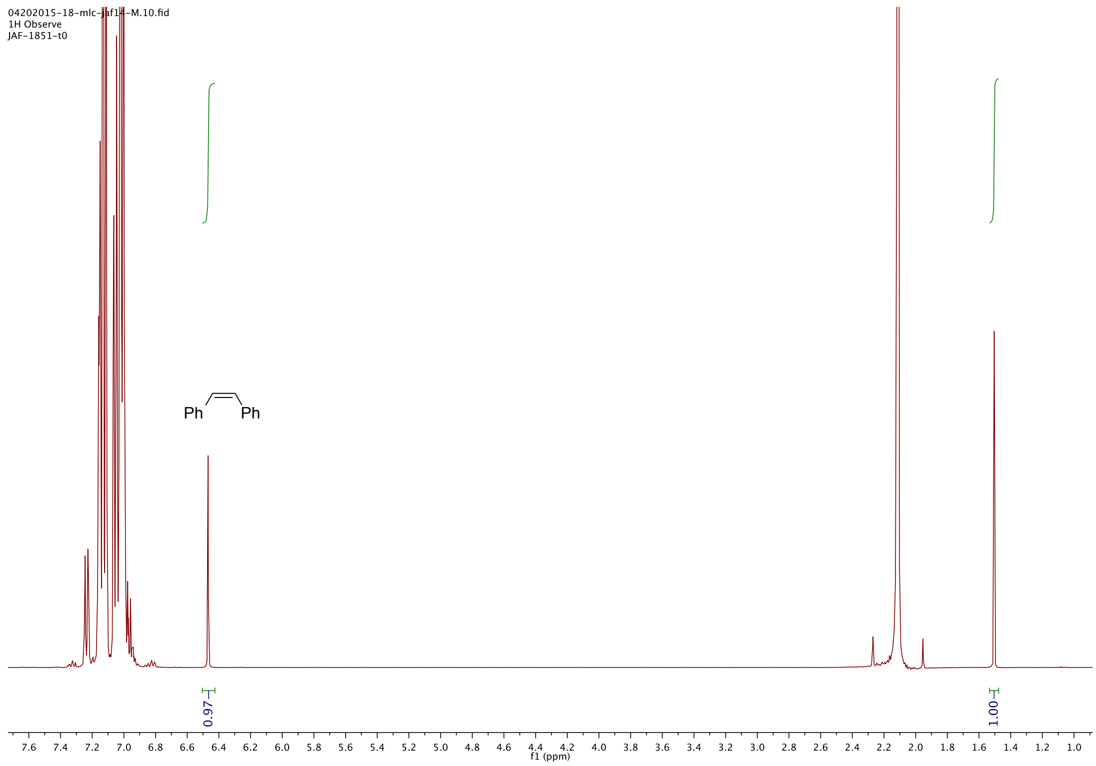

^{1}H NMR (toluene) of the crude reaction mixture showing that a greater proportion of the aldehydes produced have ^{1}H isotope at the formyl and beta positions.

A very high proportion of resonance H^{c} is ^{1}H isotope; this suggests D-H exchange occurs more rapidly than hydroformylation or there is a significant kinetic isotope effect on the migratory insertion of alkenes into the Rh-hydride/Rh-D.

While we have not done a quantitative kinetic study, the fact that similar conversions are observed in 45 minutes in transfer deuteroformylation as ATHF, would suggest there is not a large kinetic isotope effect (i.e. 3-7) on this reaction, which may have been expected if the rate-determining step was either C-H activation of formaldehyde or the formation of the formyl C-H bond of the product. Conventional hydroformylations also do not have large kinetic isotope effects.[13]

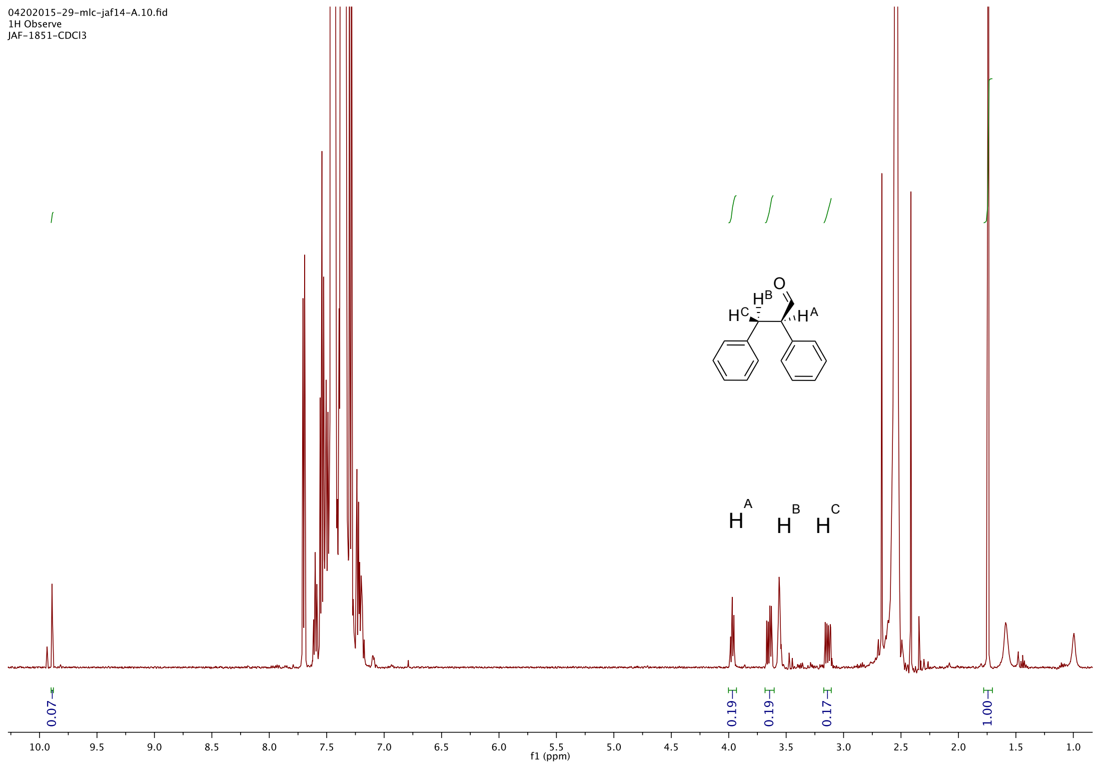

8. Formation of [RhH(CO)$_2$Ph-BPE)] under ATHF conditions. Coordination studies.

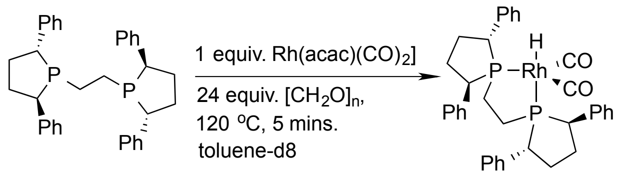

A Biotage 2.5 ml microwave vial containing a stirring bar was charged with [Rh(acac)(CO)$_2$] (8.6 mg, 0.0333 mmol), (*S,S*)-Ph-BPE (16.8 mg, 0.0333 mmol) and paraformaldehyde (24 equiv, 100 mg). The vial was sealed with a crimp cap, purged with three vacuum/argon cycles and left under an argon atmosphere. Toluene-d8 (1 mL) was added and the solution was heated to 120 °C using microwave radiation. After 5 min, the vial was cooled down and using a 20 mL syringe the solution (and H_2/CO) was taken from the vial and injected in an NMR tube placed under an Ar atmosphere. The NMR data obtained matched the reported data in the literature for the same complex formed under standard hydroformylation conditions.[16]

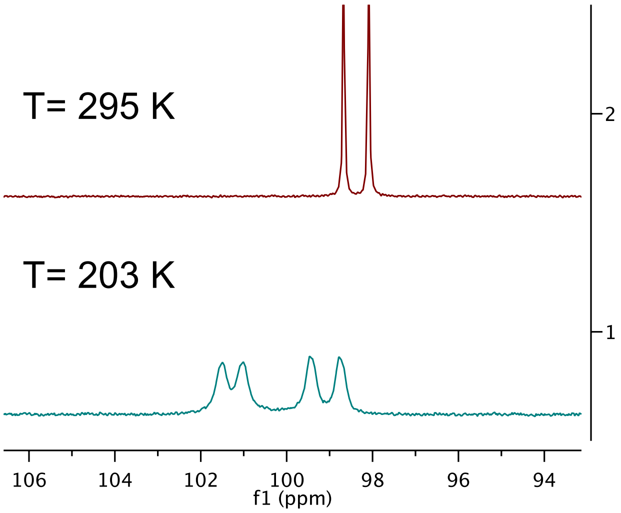

^{31}P{^{1}H}-NMR spectrum of [RhH(CO)$_2$((*S,S*)-Ph-BPE)] at rt (top). The presence of a doublet suggests an equatorial-axial (ea) coordination mode with fast exchanging of P atoms on the NMR time scale. $^2J_{P\text{-}P}$ could not be resolved.[16] ^{31}P{^{1}H}-NMR (202 MHz, Toluene-*d*$_8$) δ 98.38 (d, $^1J_{Rh\text{-}P}$ = 120 Hz). Cooling the sample to 203K resulted in the appearance of a dd indicative of two P atoms, but $^2J_{P\text{-}P}$ was still not resolved. ^{31}P{^{1}H}-NMR (202 MHz, Toluene-*d*$_8$) δ 101.25 (d, $^1J_{Rh\text{-}P}$ = 98 Hz), 99.12 (d, $^1J_{Rh\text{-}P}$ = 140 Hz).

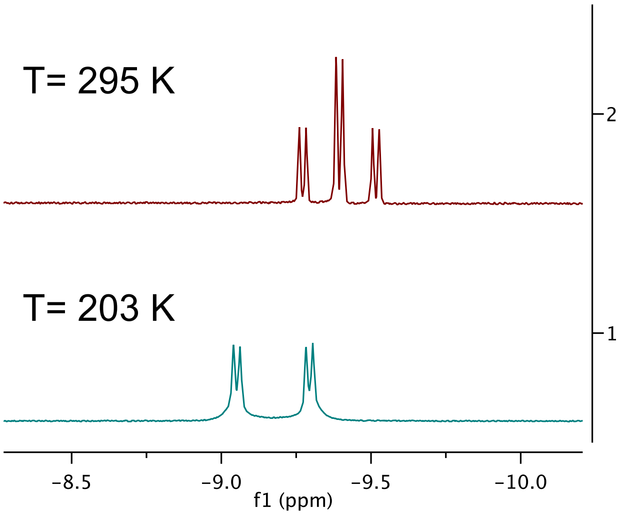

^{1}H-NMR spectrum of [RhH(CO)$_2$((*S,S*)-Ph-BPE)] showing the hydride region at rt (top). The presence of a triplet of doublets and the $^2J_{P\text{-}H}$ value suggest an equatorial-axial (ea) coordination mode with fast exchanging of P atoms on the NMR time scale.[16] ^{1}H-NMR (500 MHz, Toluene-*d*$_8$) δ -9.39 (td, $^1J_{P\text{-}H}$ = 61 Hz, $^1J_{Rh\text{-}H}$ = 11 Hz, 1H). Cooling the sample to 203K changed the signal to a broad dd. The resolution is not good enough to observe the expected ddd and the small *cis* $^1J_{P\text{-}H}$ coupling. ^{1}H-NMR (500 MHz, Toluene-*d*$_8$) δ -9.17 (dd, $^2J_{P\text{-}H}$ = 121 Hz, $^1J_{Rh\text{-}H}$ = 11 Hz, 1H).

9. NMR spectra of selected compounds

NMR spectra of (*Z*)-1,2-bis(4-methoxyphenyl)ethene

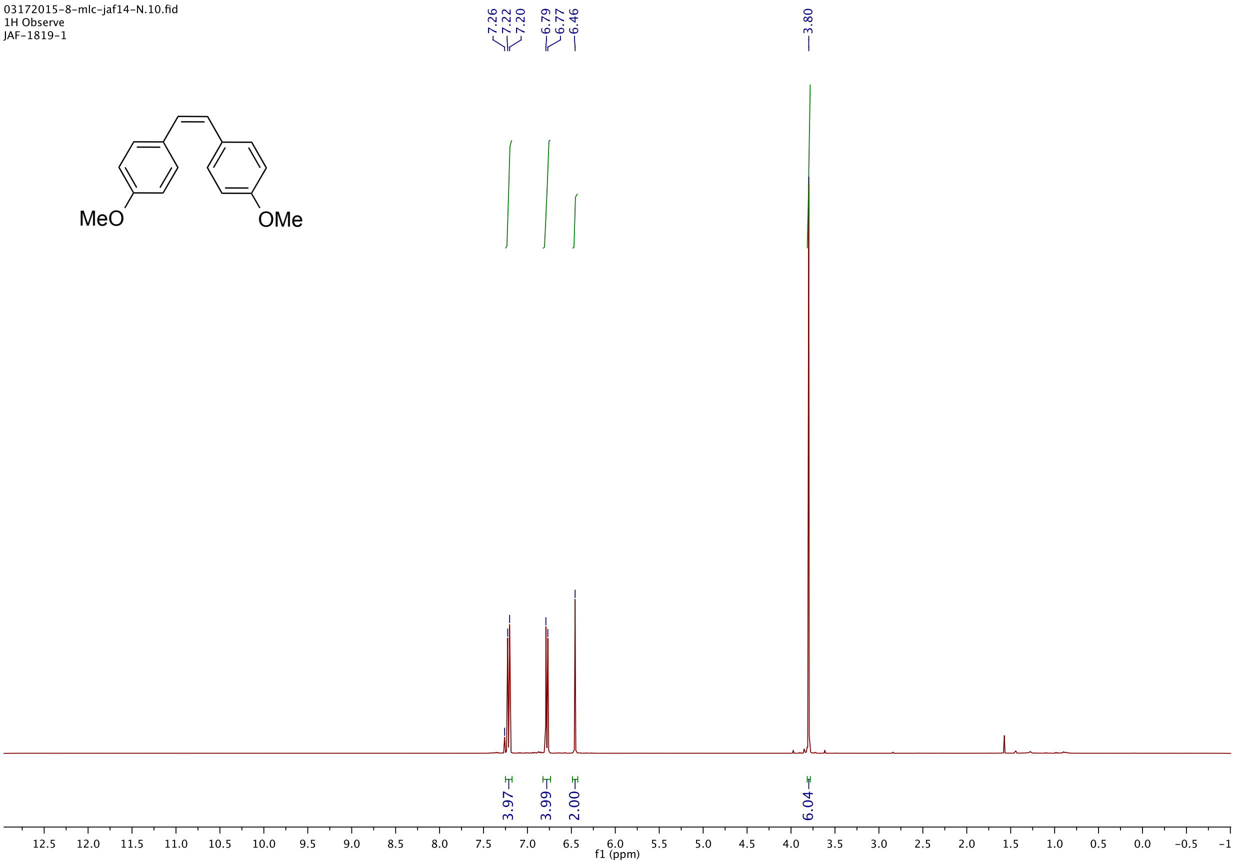

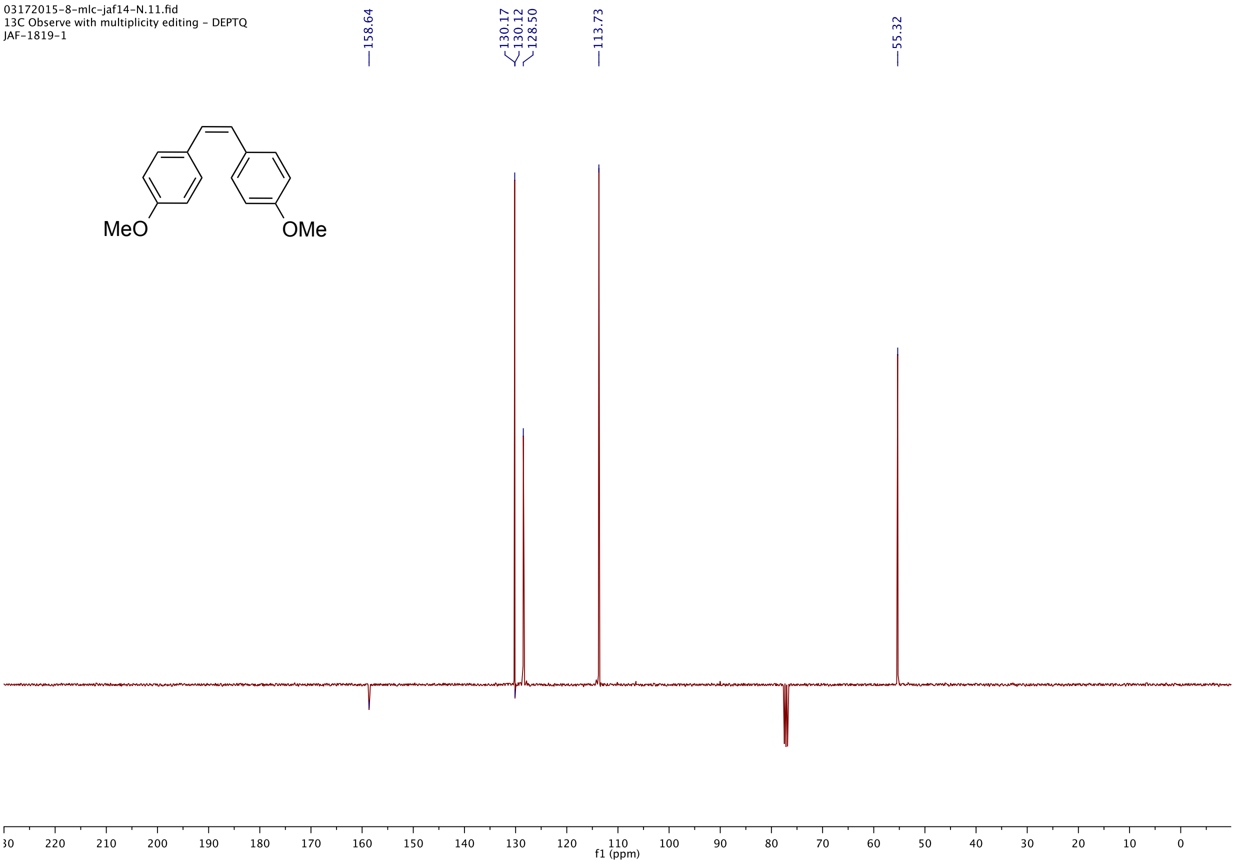

NMR spectra of (*Z*)-1,2-bis(3-methoxyphenyl)ethene[6]

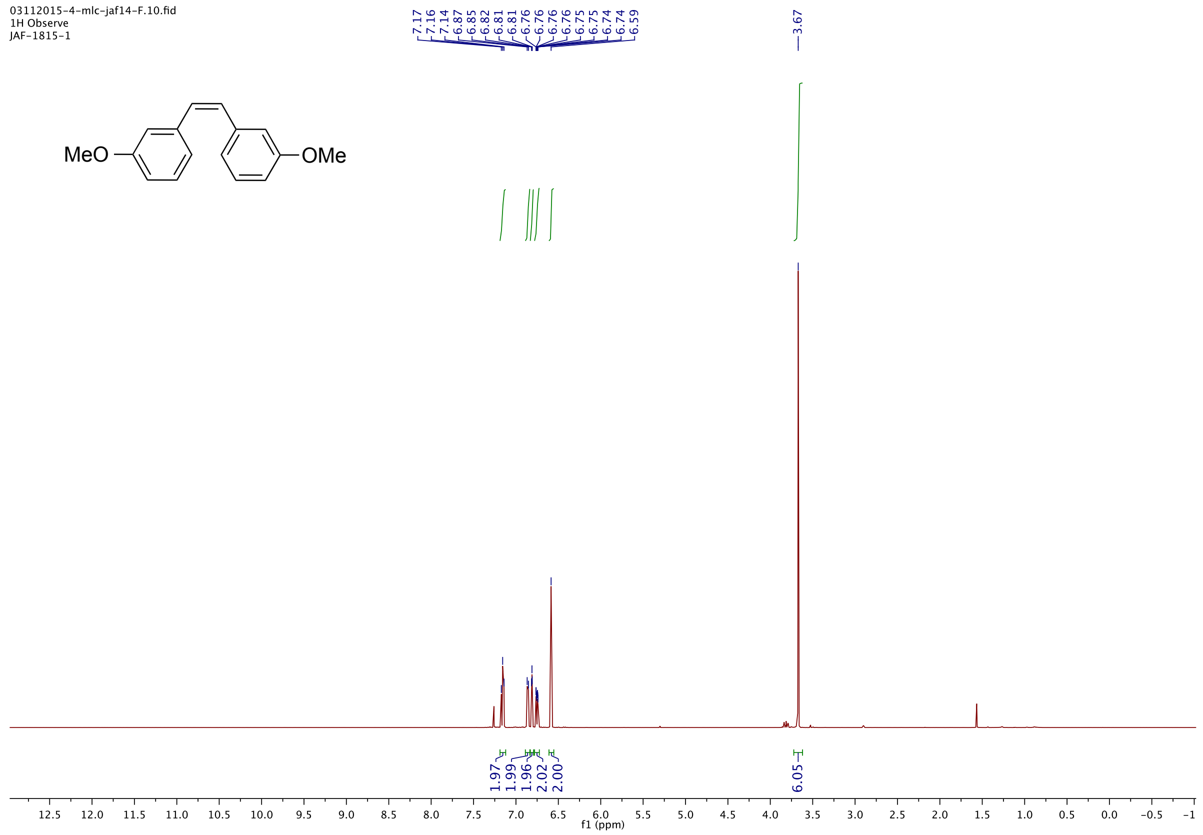

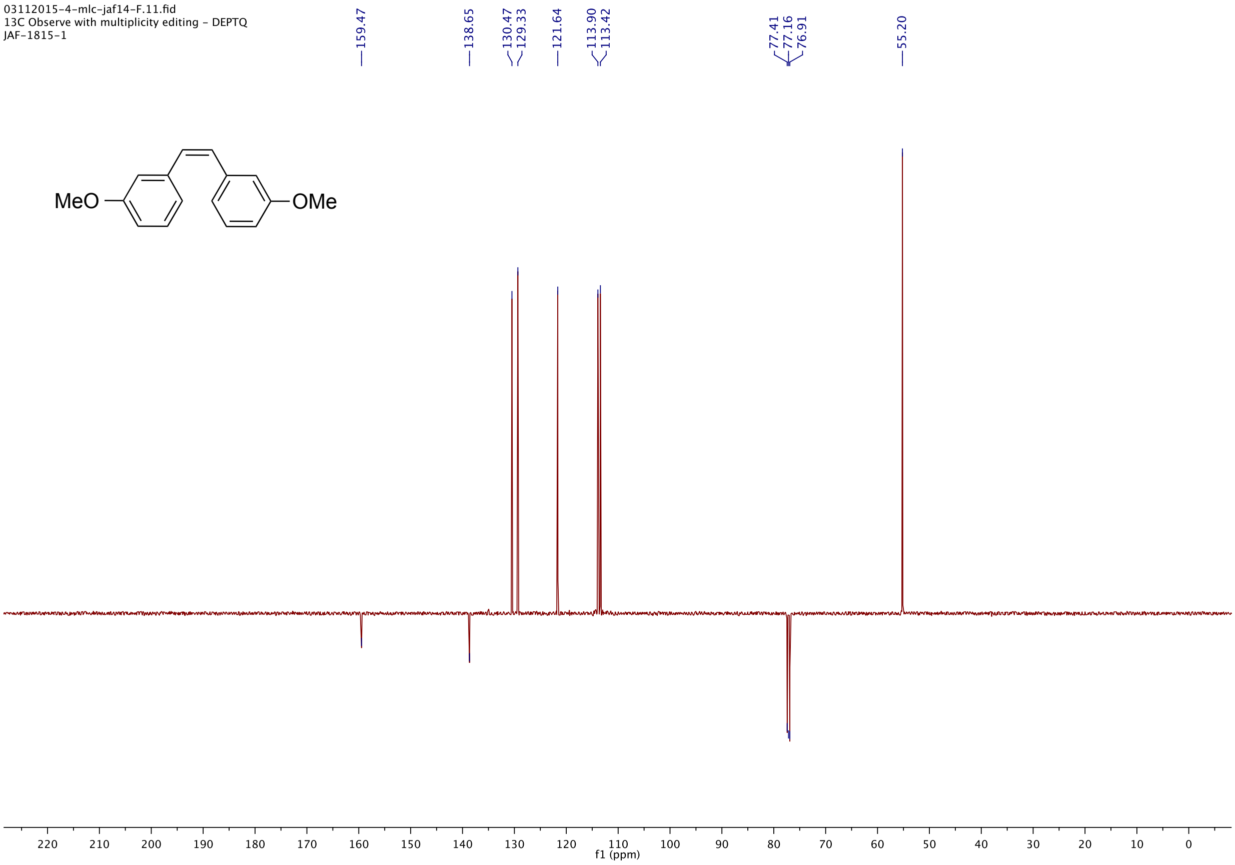

NMR spectra of (*Z*)-methyl -4-(4-methoxystyryl)benzoate

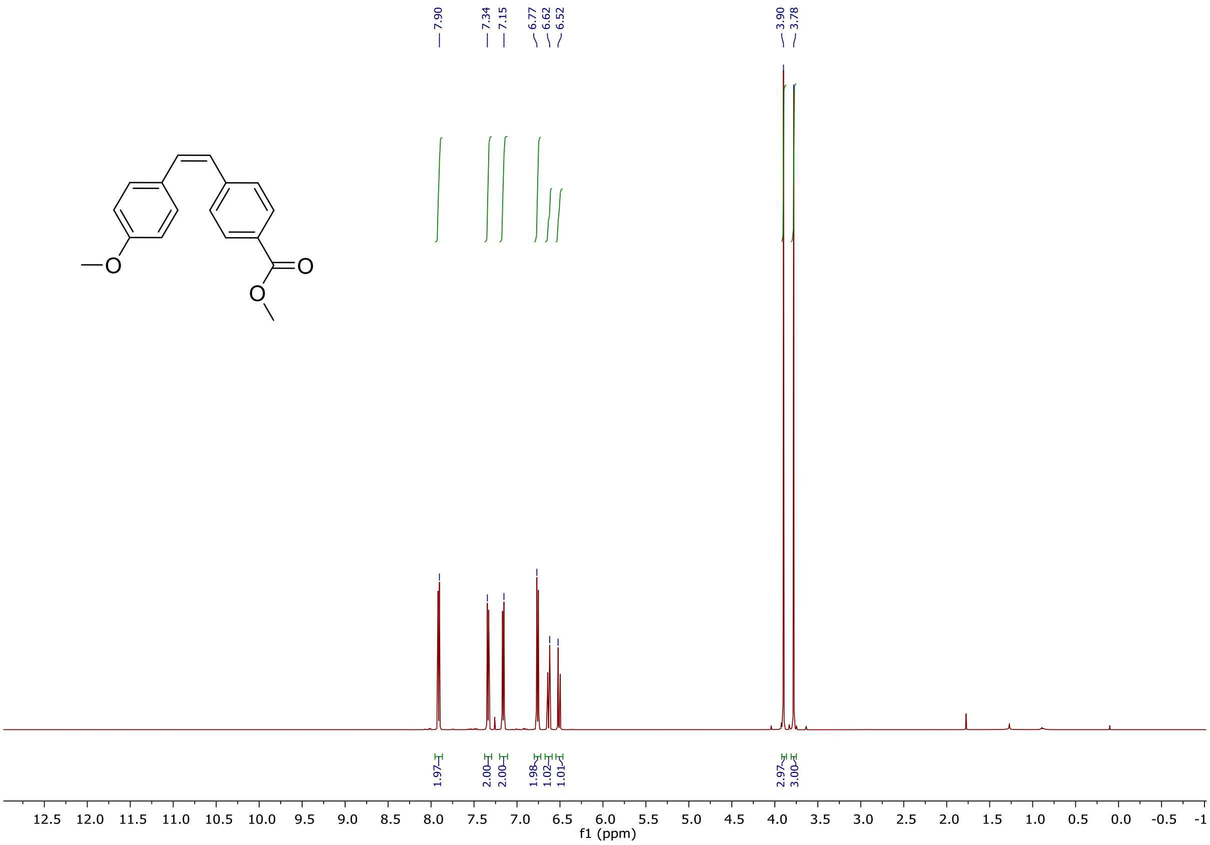

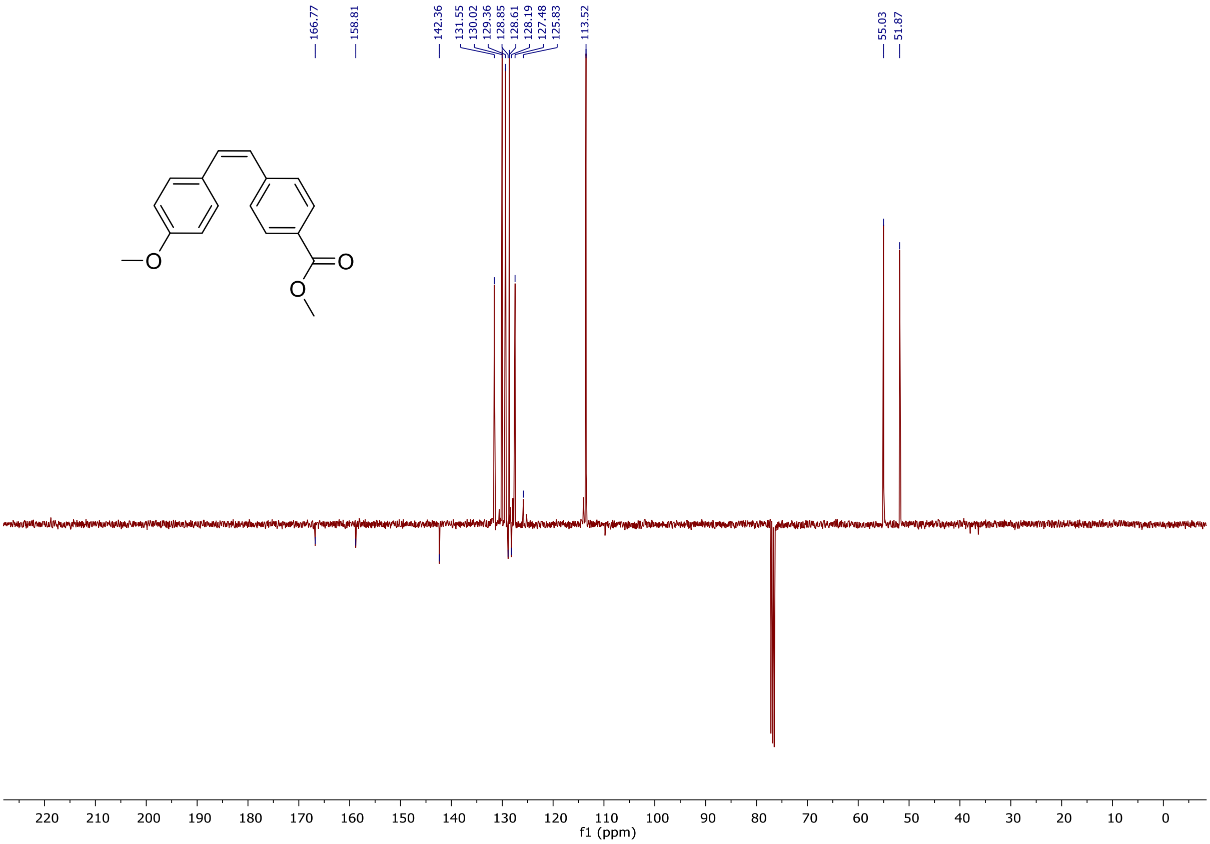

NMR spectra of 2,3-diphenylpropan-1-ol

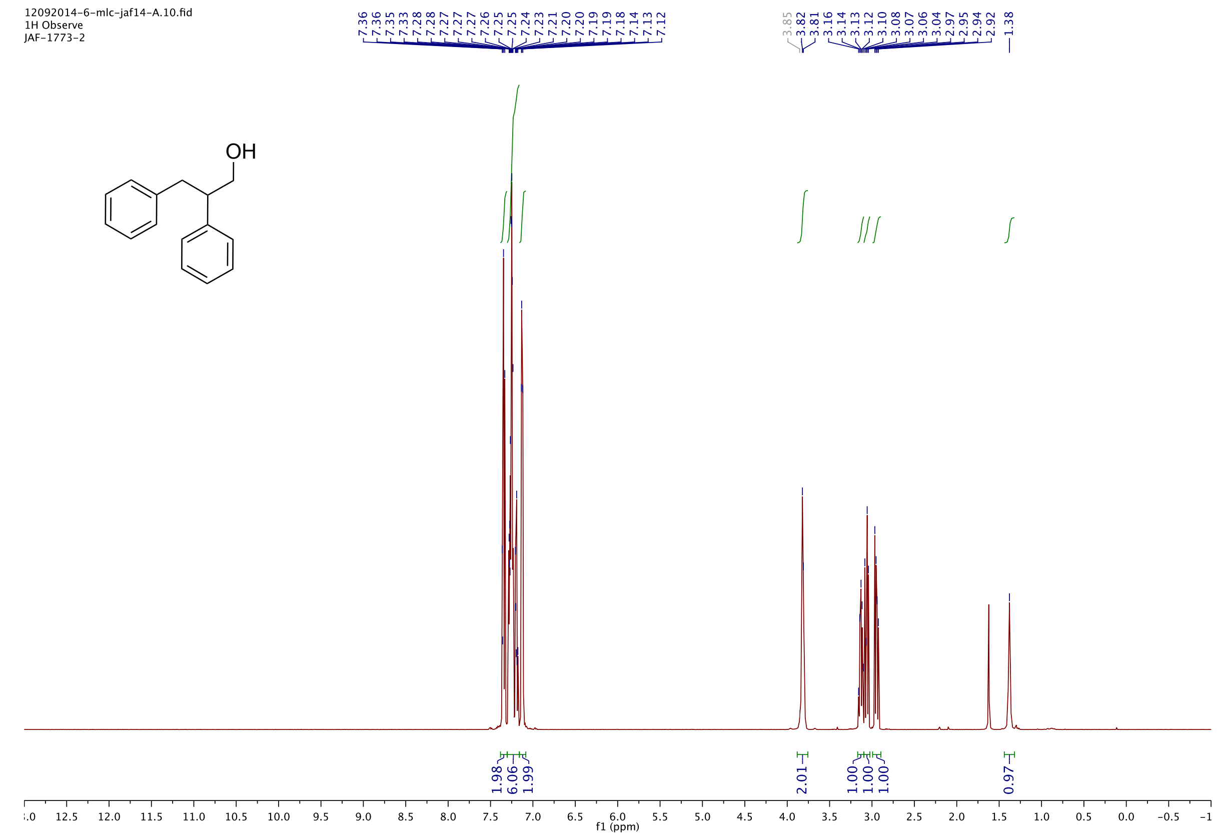

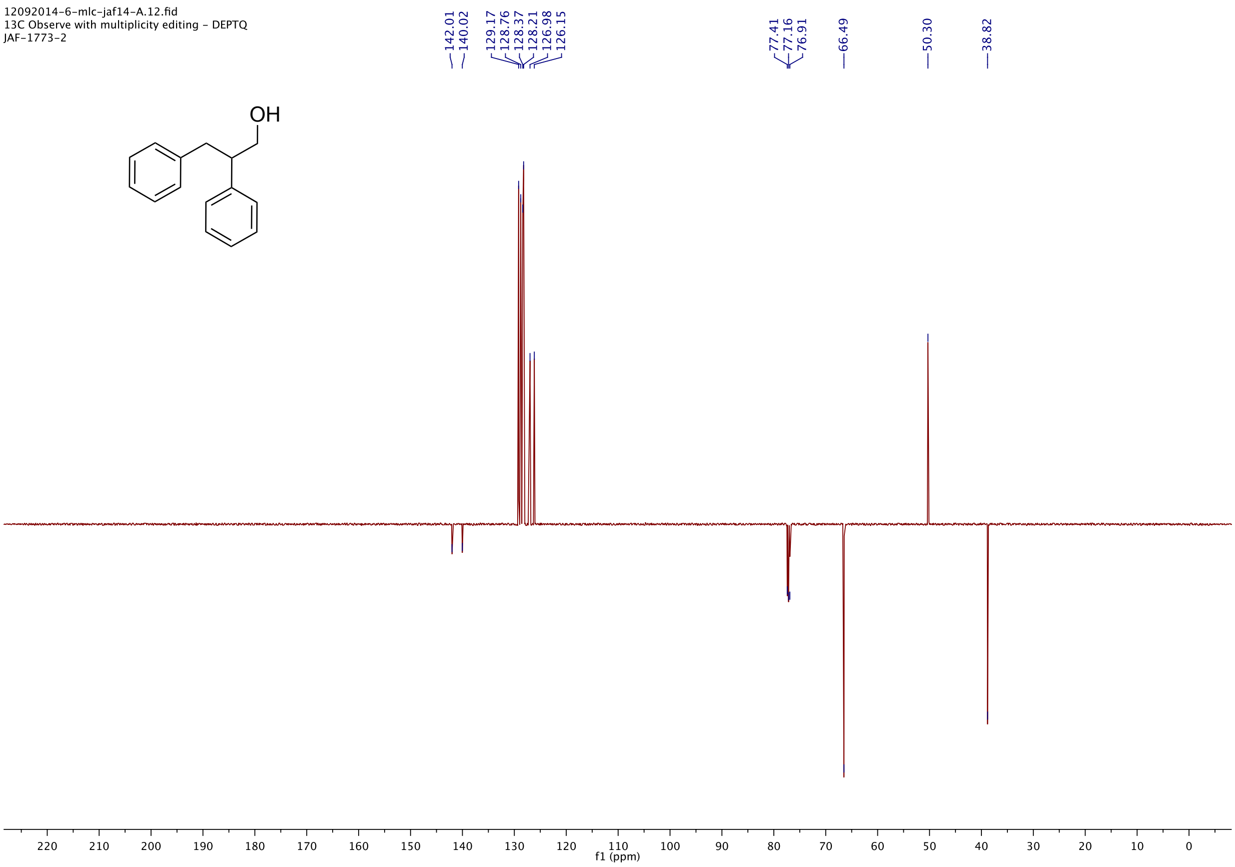

NMR spectra of 2,3-bis(3-methoxyphenyl)propan-1-ol

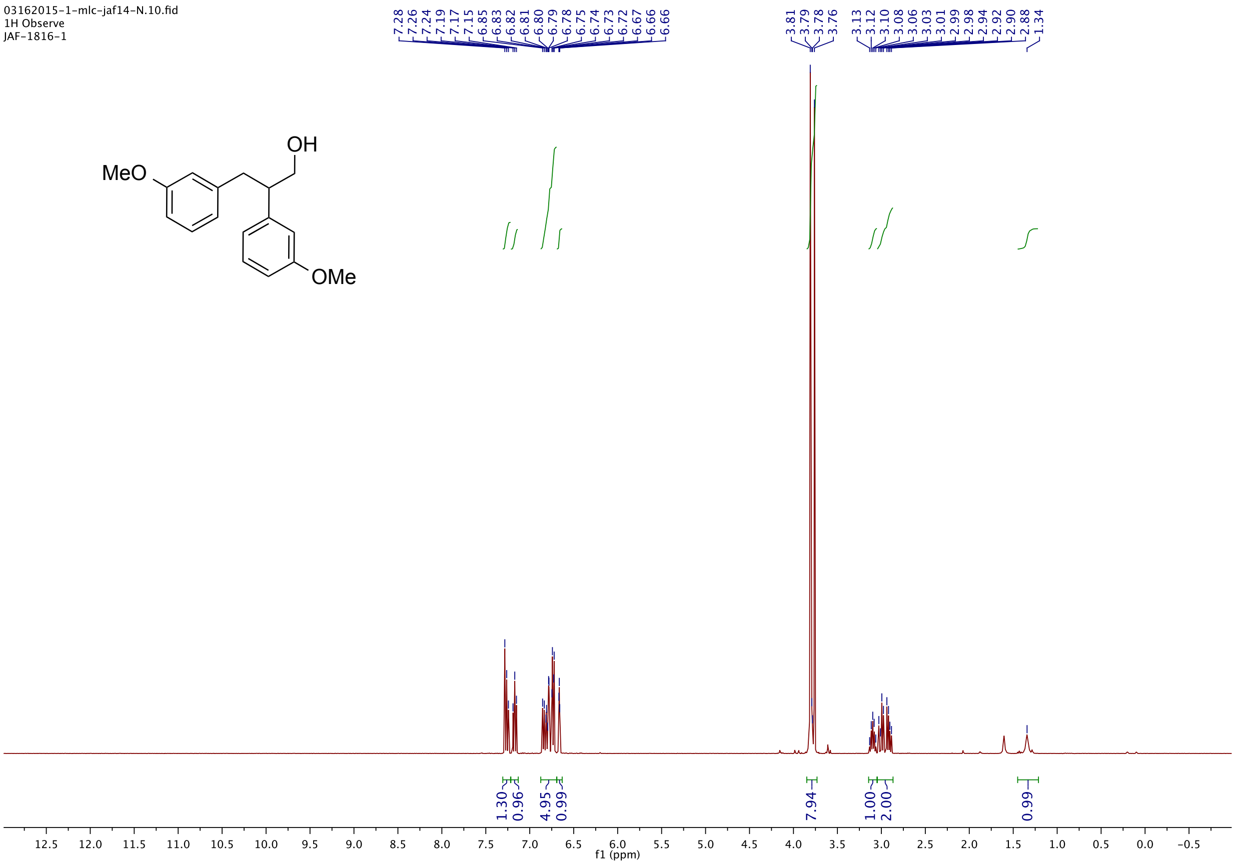

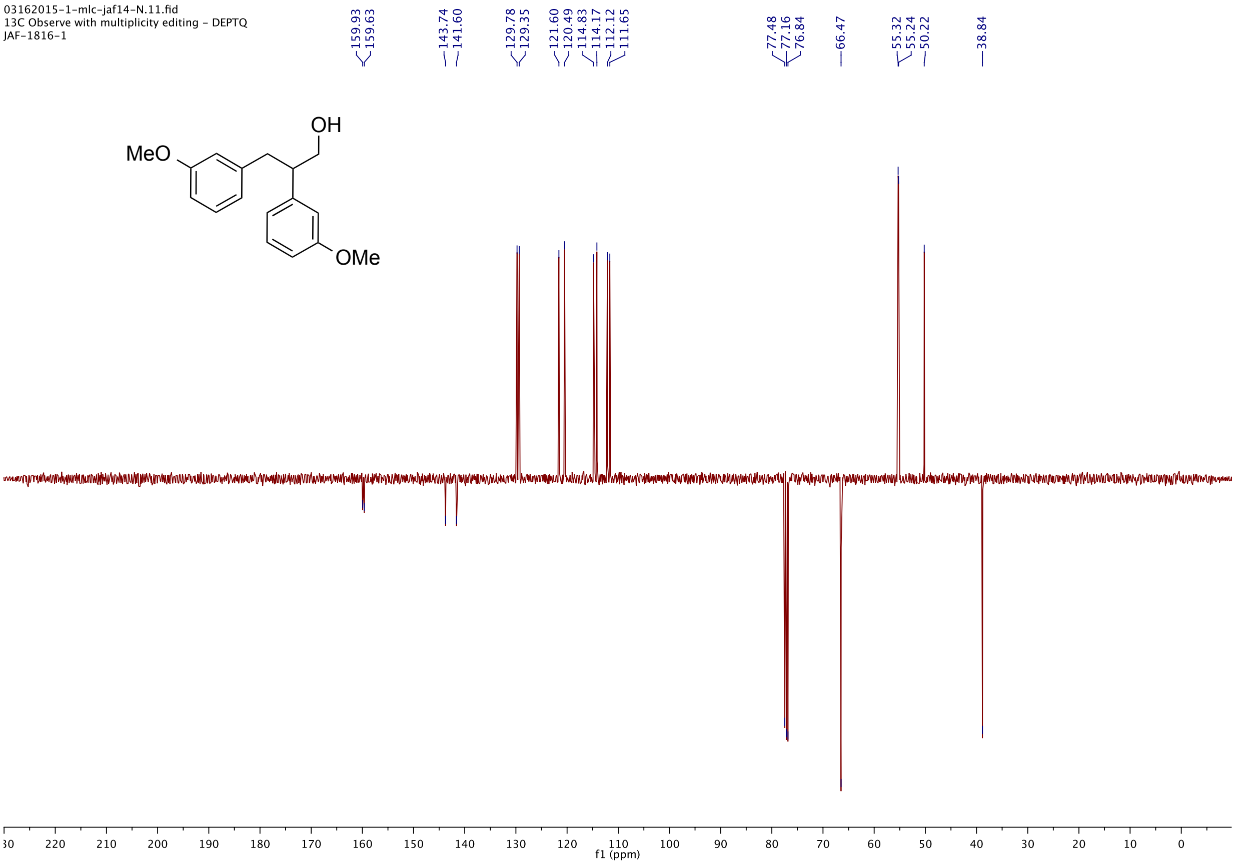

NMR spectra of 2,3-bis(4-methoxyphenyl)propan-1-ol[9]

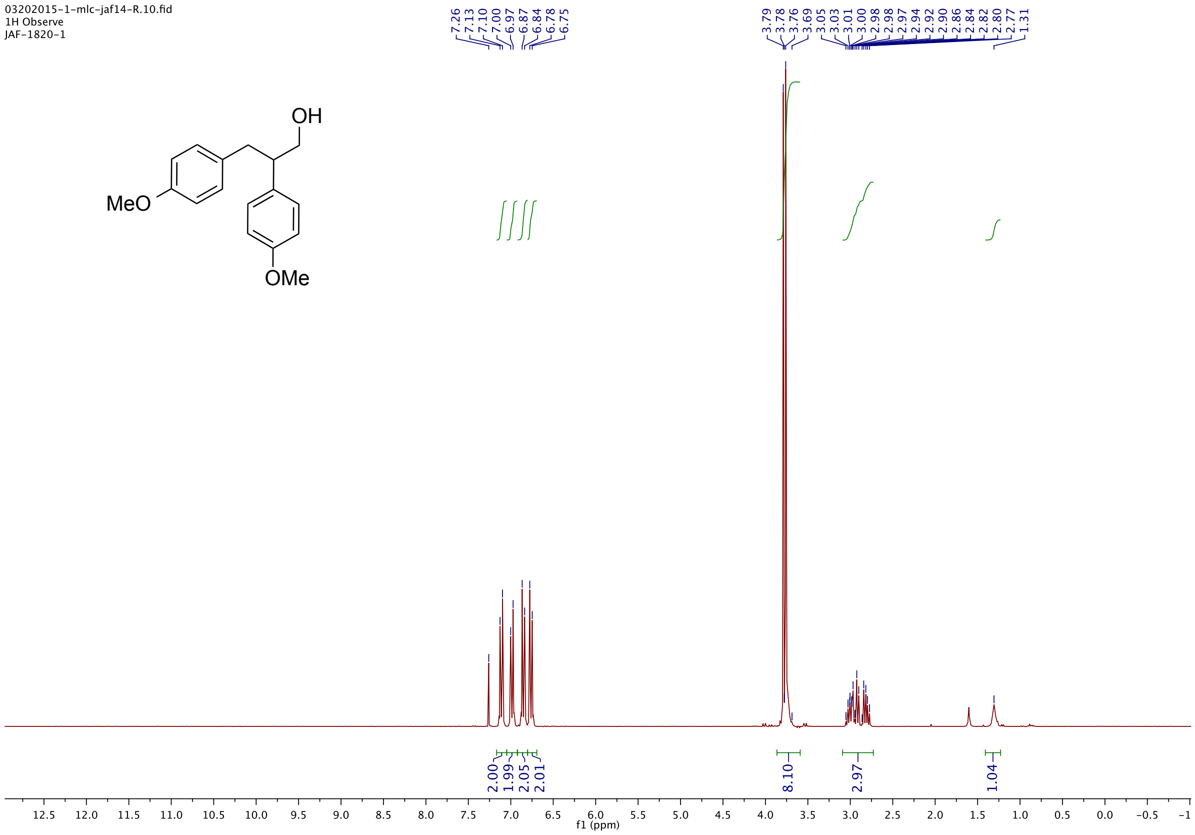

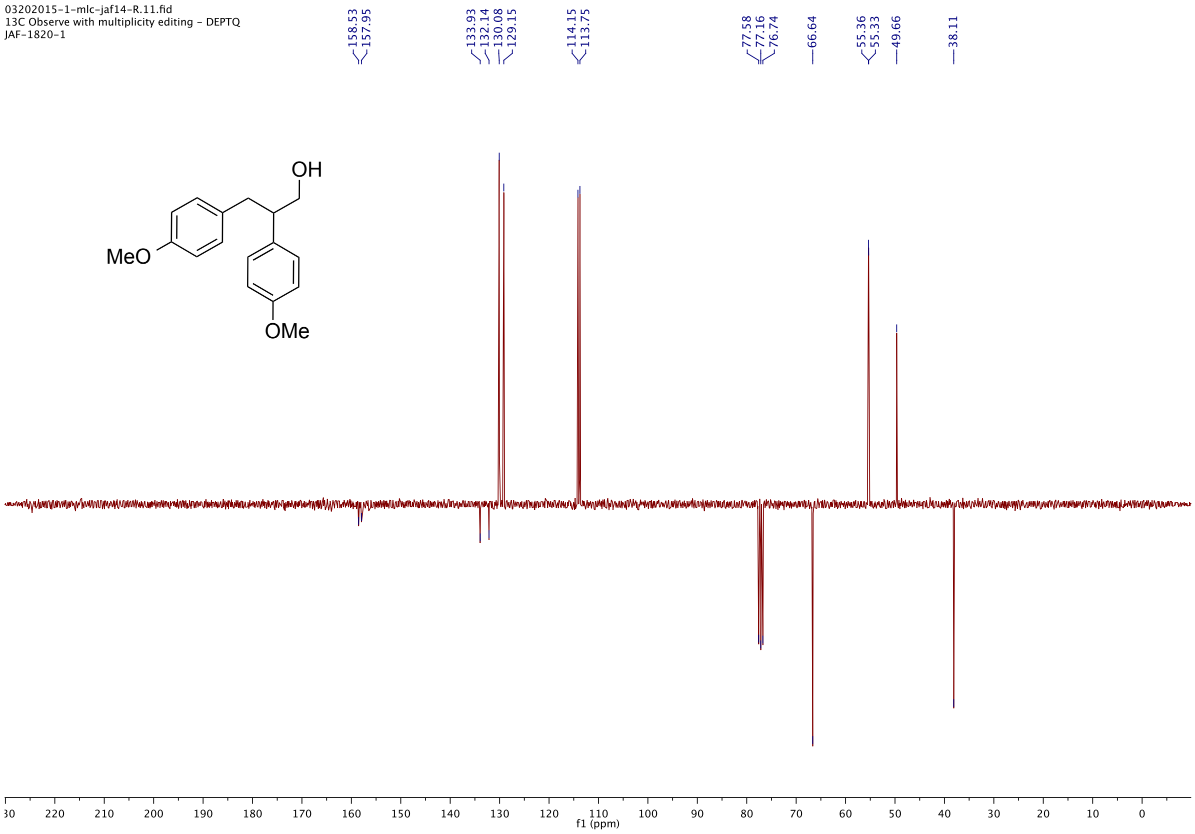

NMR spectra of Methyl 4-(1-hydroxy-3-(4-methoxyphenyl)propan-2-yl)benzoate and methyl 4-(3-hydroxy-2-(4-methoxyphenyl)propyl)benzoate

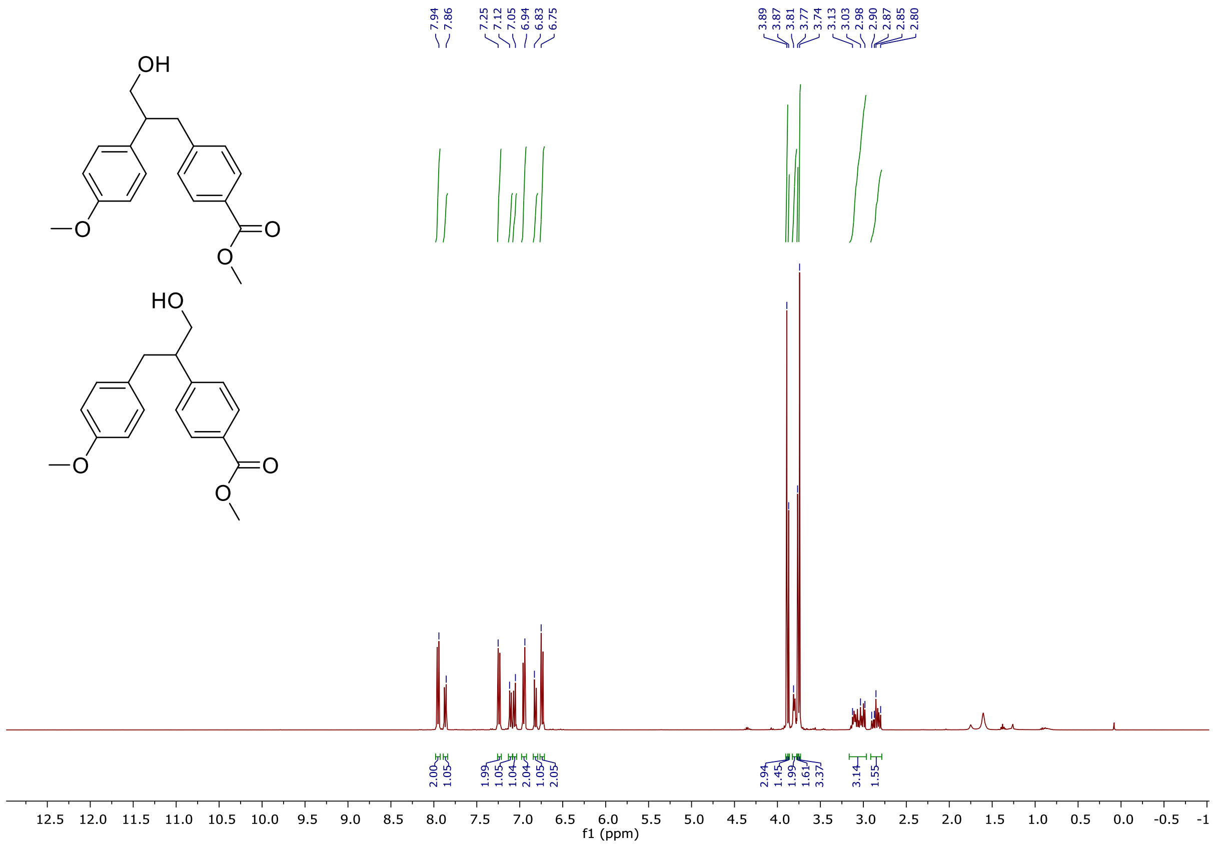

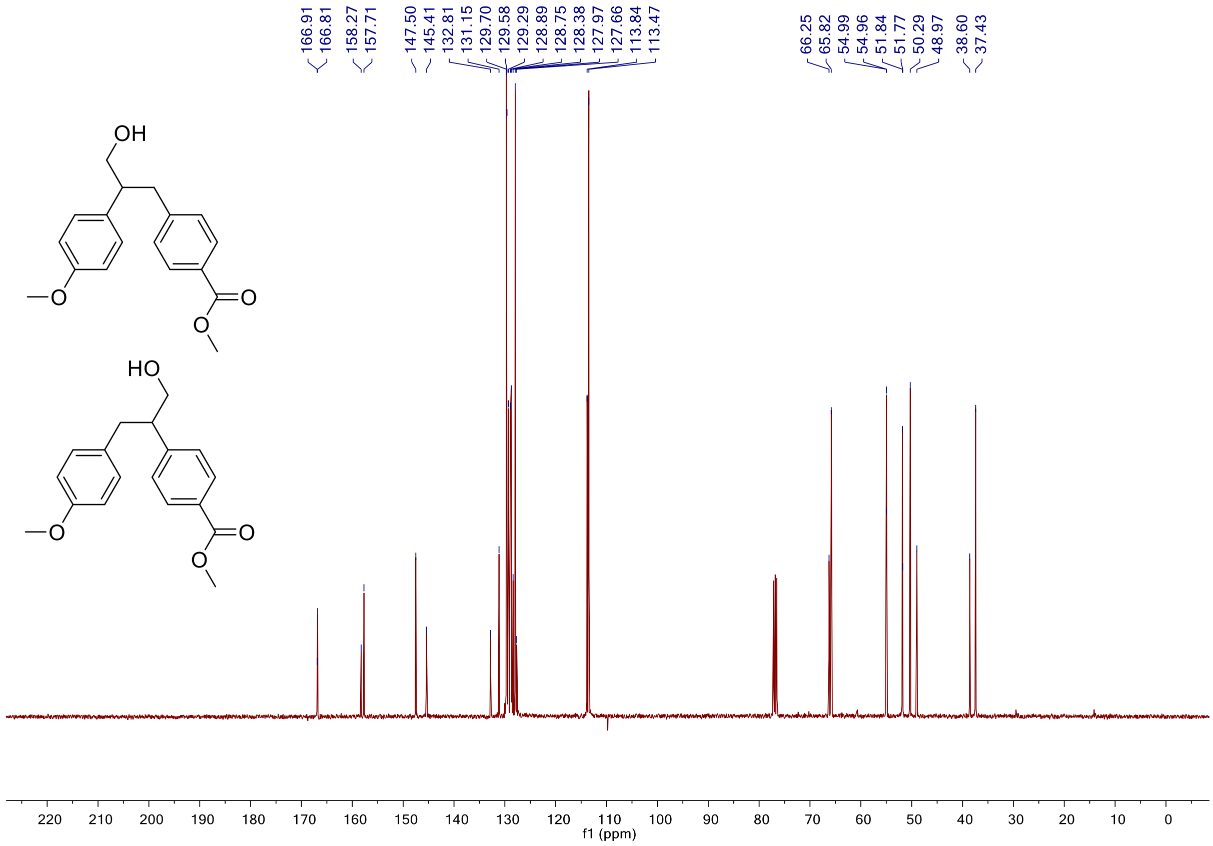

NMR spectra of cyclopentanecarboxylic acid

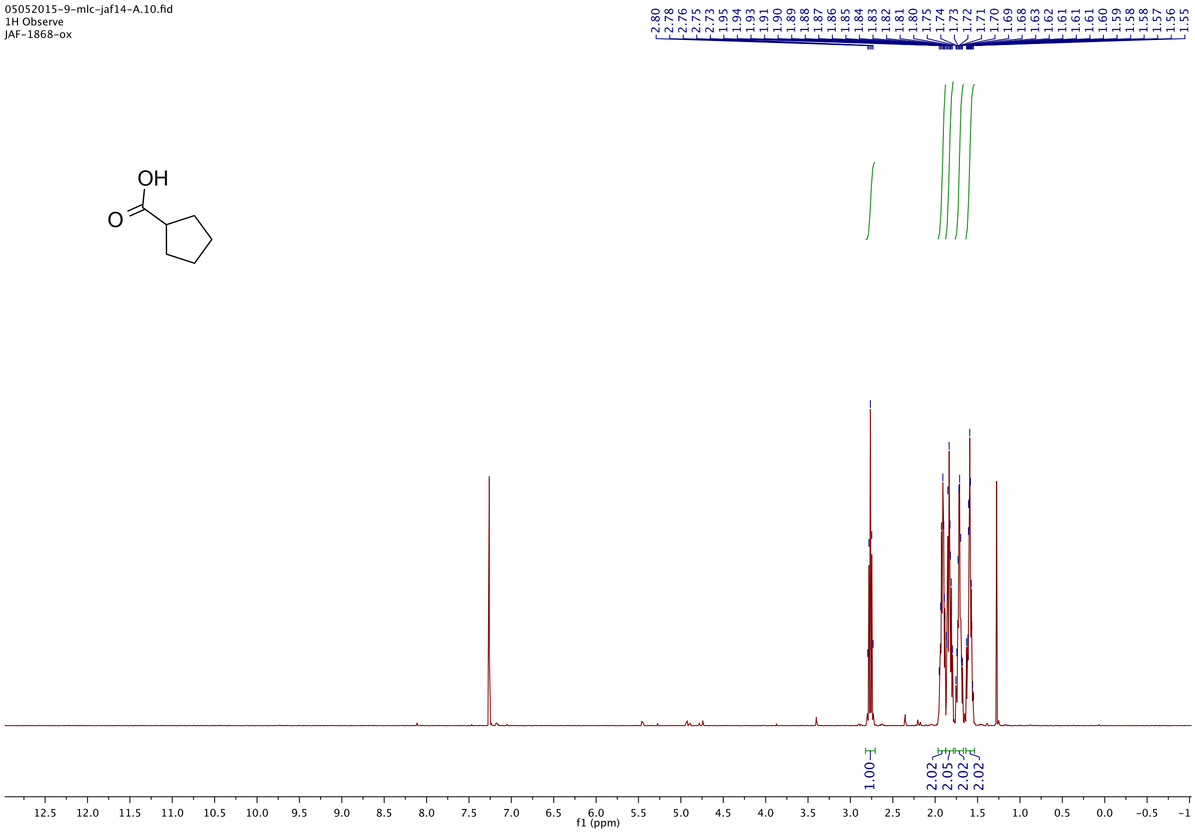

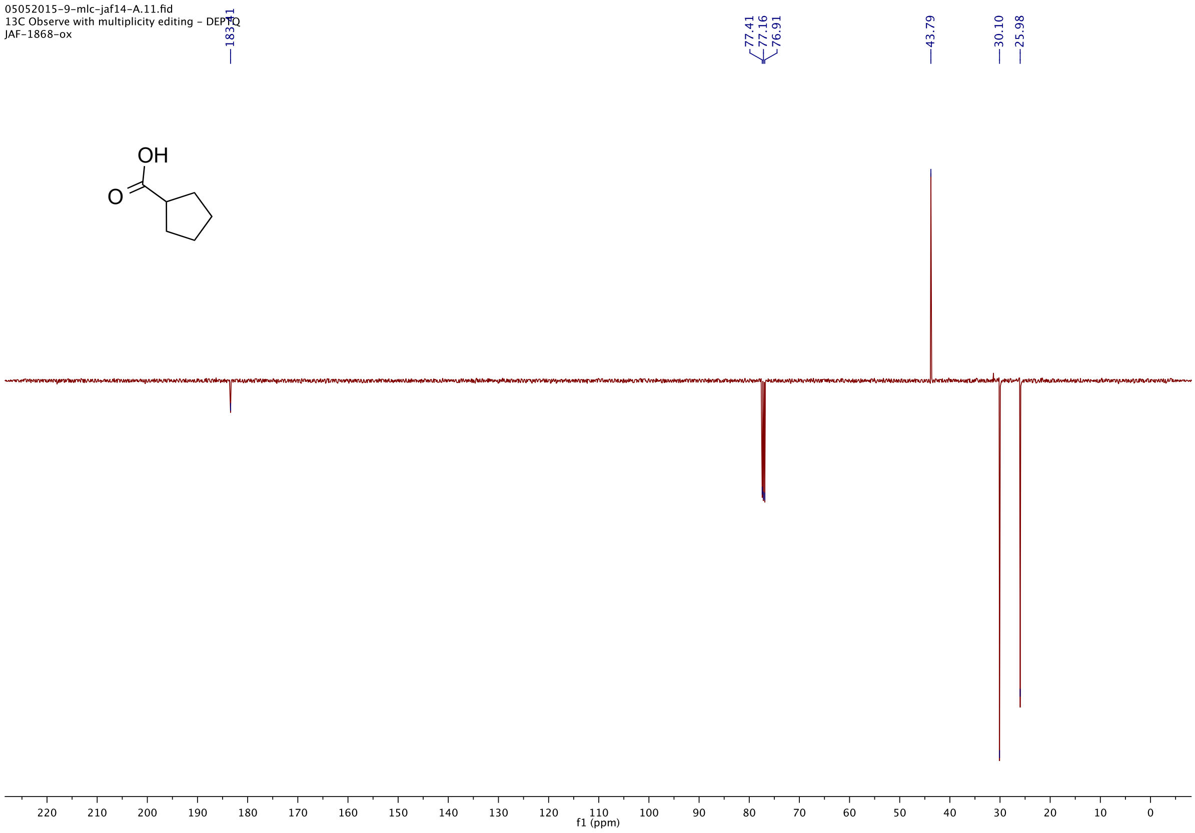

NMR spectra of (1-tosylpyrrolidin-3-yl)methanol

02172015-25-mlc-jaf14-F.11.fid
1H Observe
JAF-1805-2

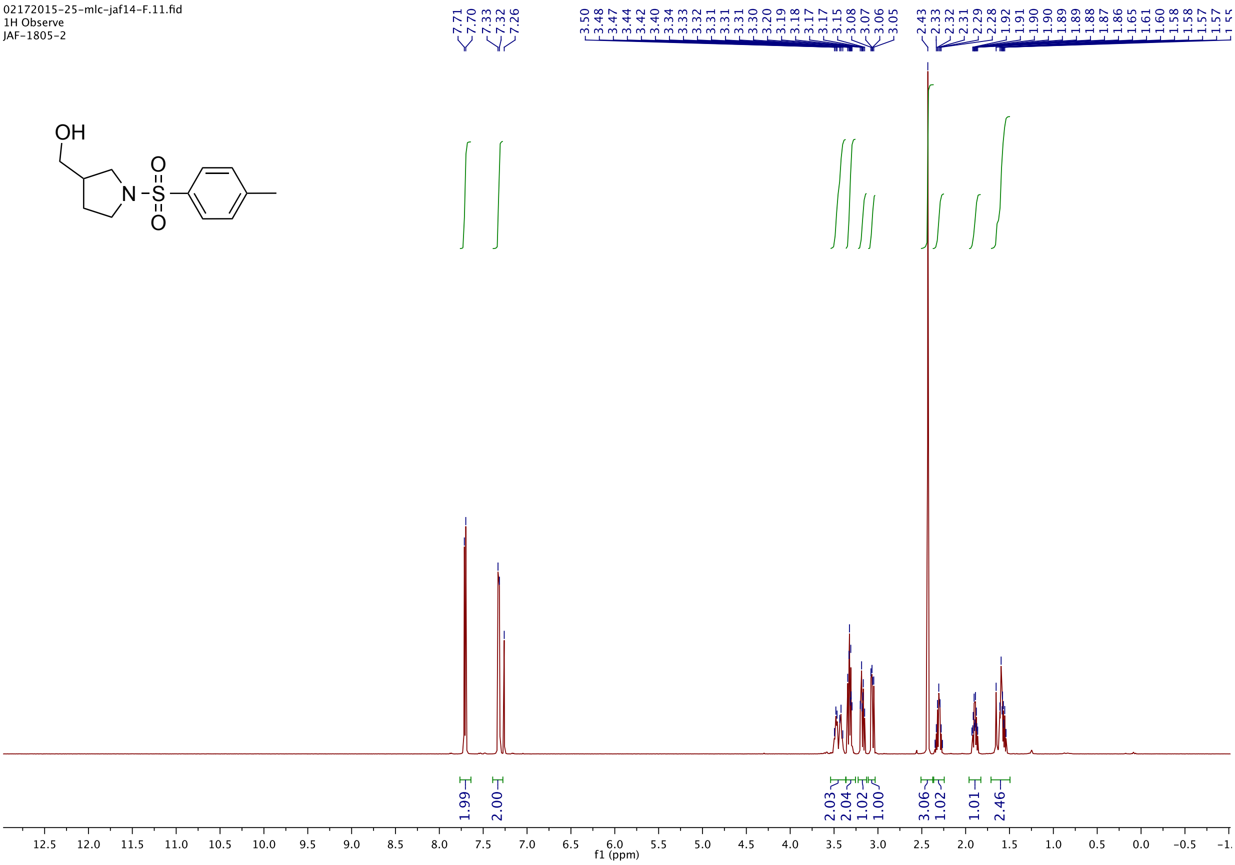

02172015-25-mlc-jaf14-F.12.fid
13C Observe with multiplicity editing - DEPTQ
JAF-1805-2

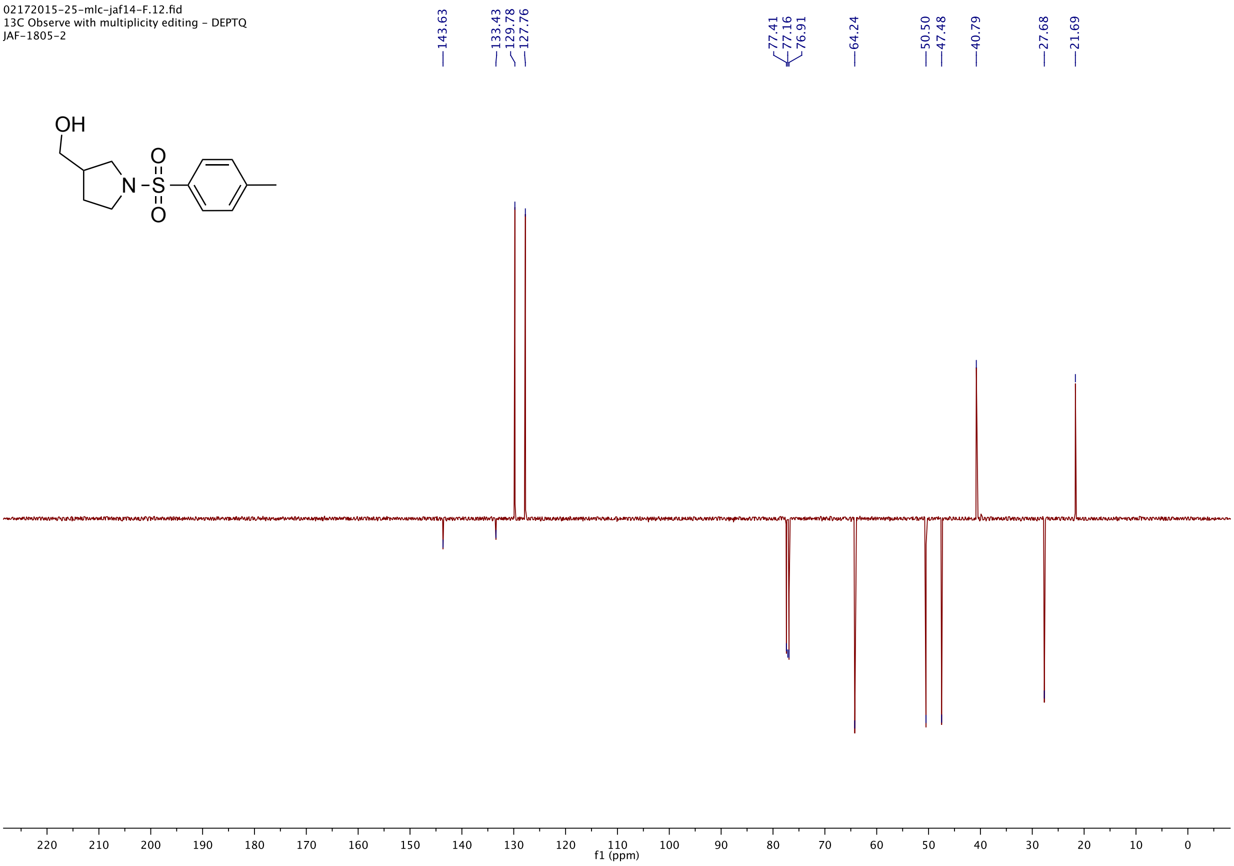

NMR spectra of (1-tosylpyrrolidin-2-yl)methanol

02172015-24-mlc-jaf14-F.10.fid
1H Observe
JAF-1805-1

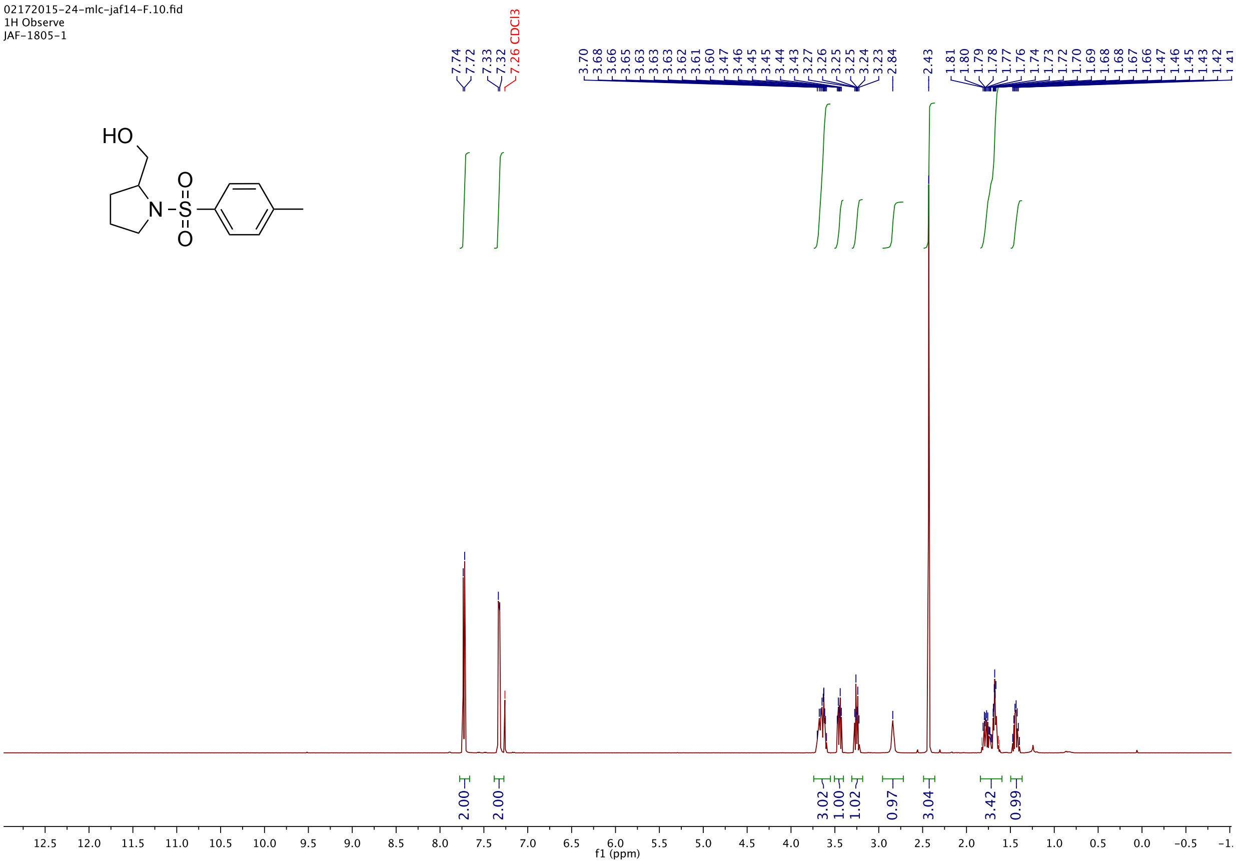

02172015-24-mlc-jaf14-F.11.fid
13C Observe with multiplicity editing - DEPTQ
JAF-1805-1

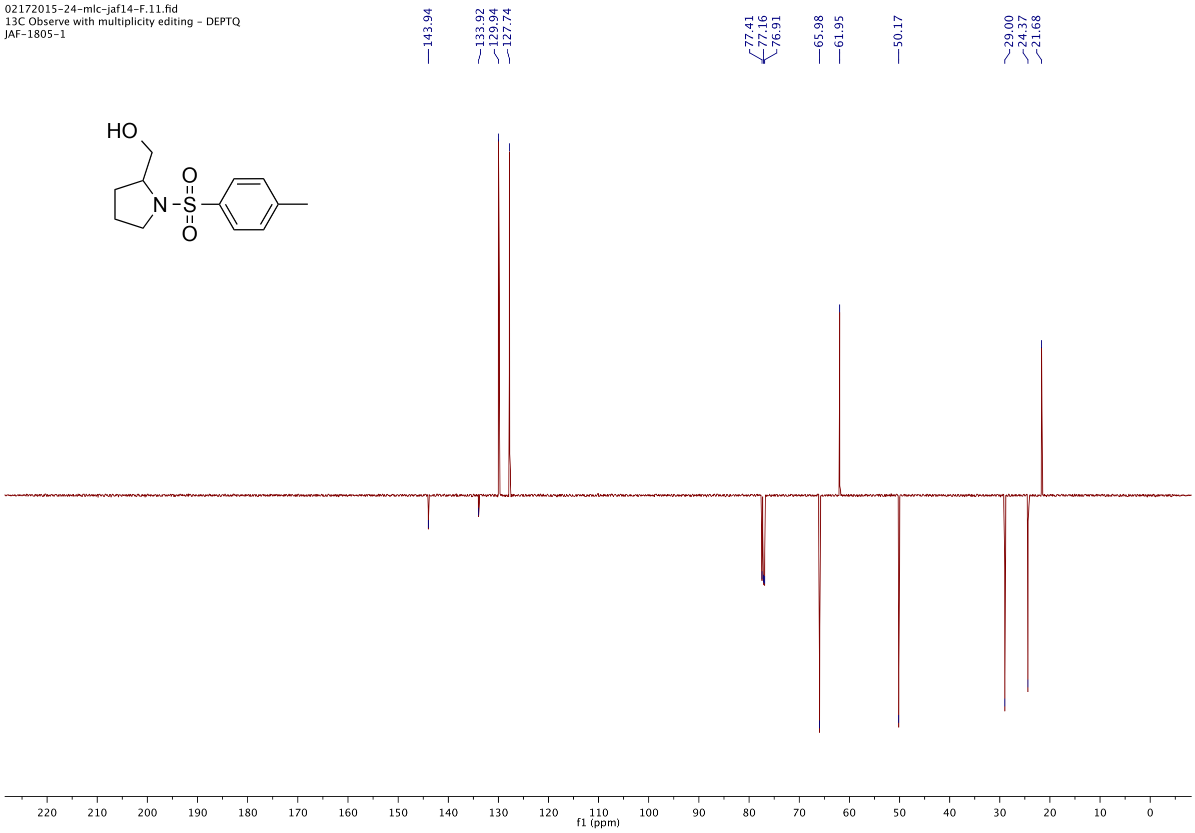

NMR spectra of benzyl 3-(hydroxymethyl)pyrrolidine-1-carboxylate

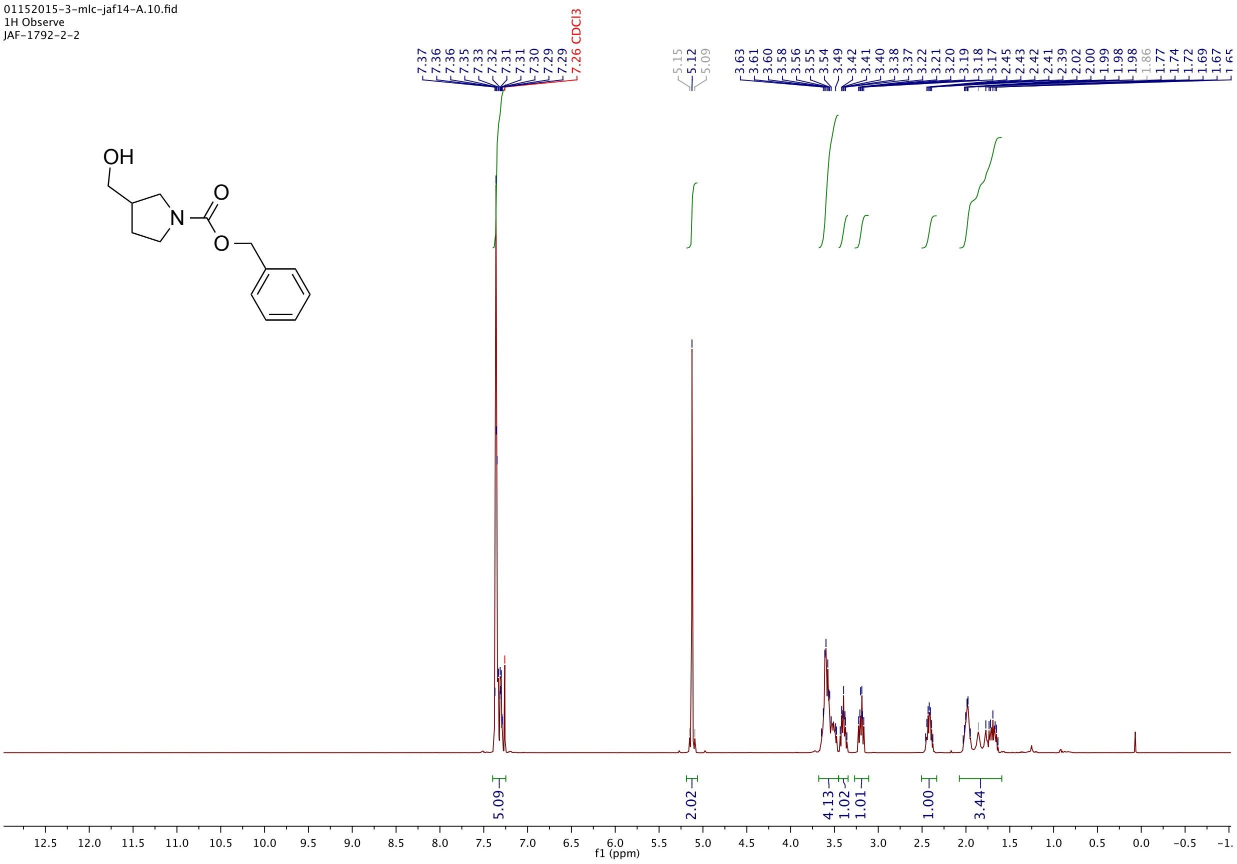

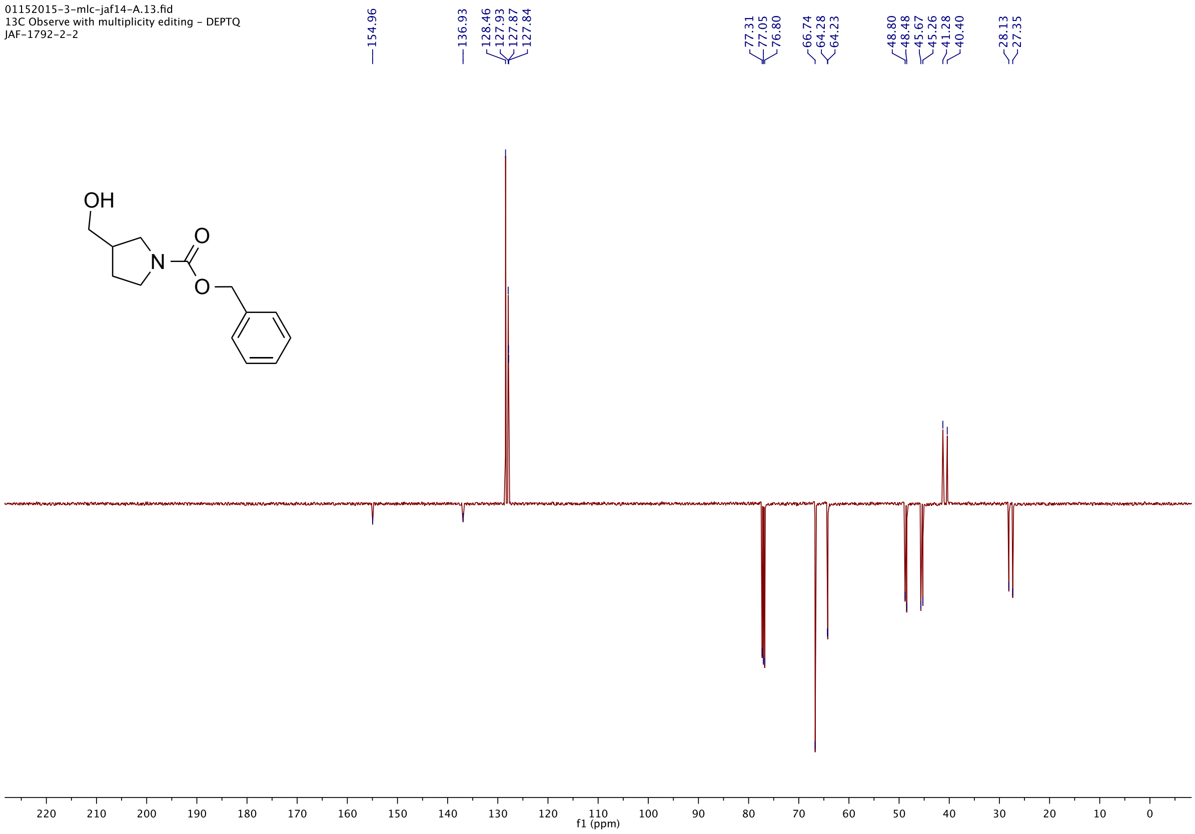

NMR spectra of benzyl 2-(hydroxymethyl)pyrrolidine-1-carboxylate

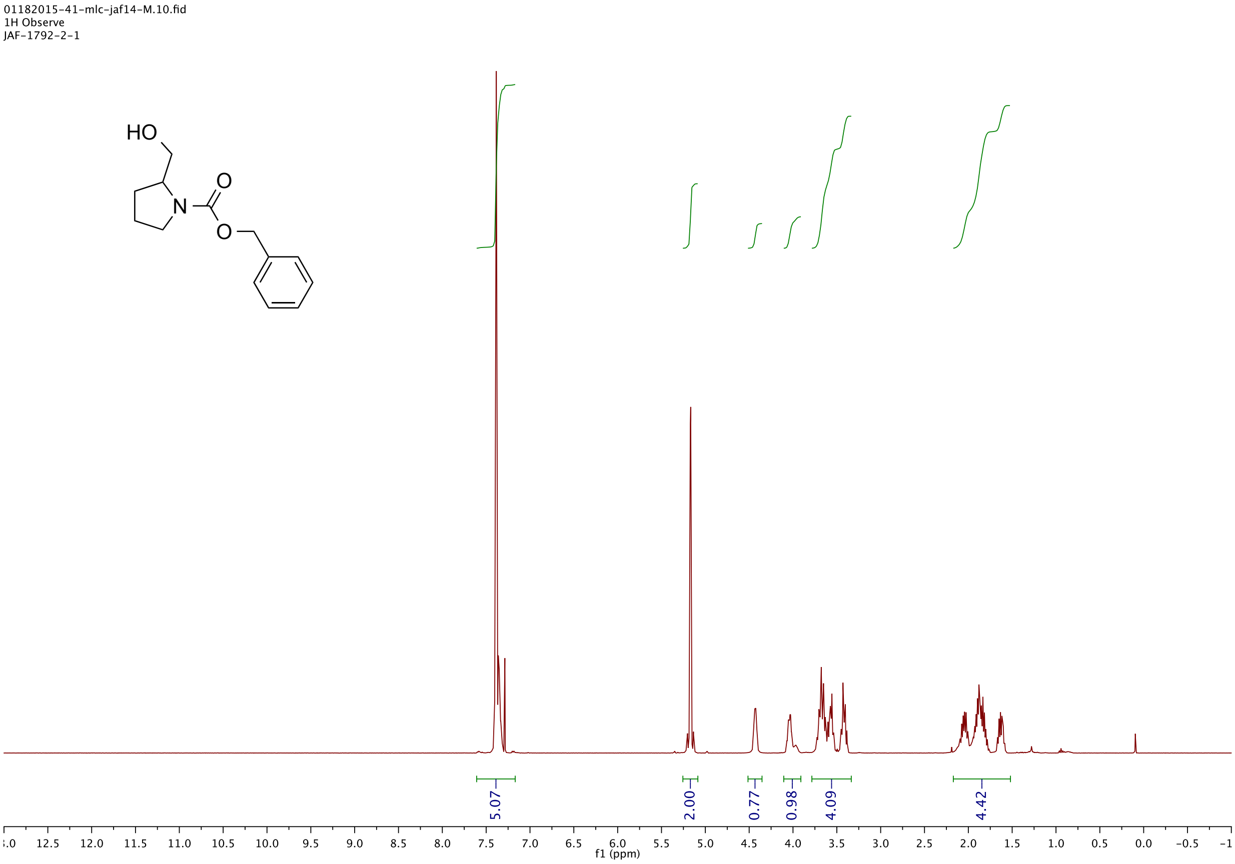

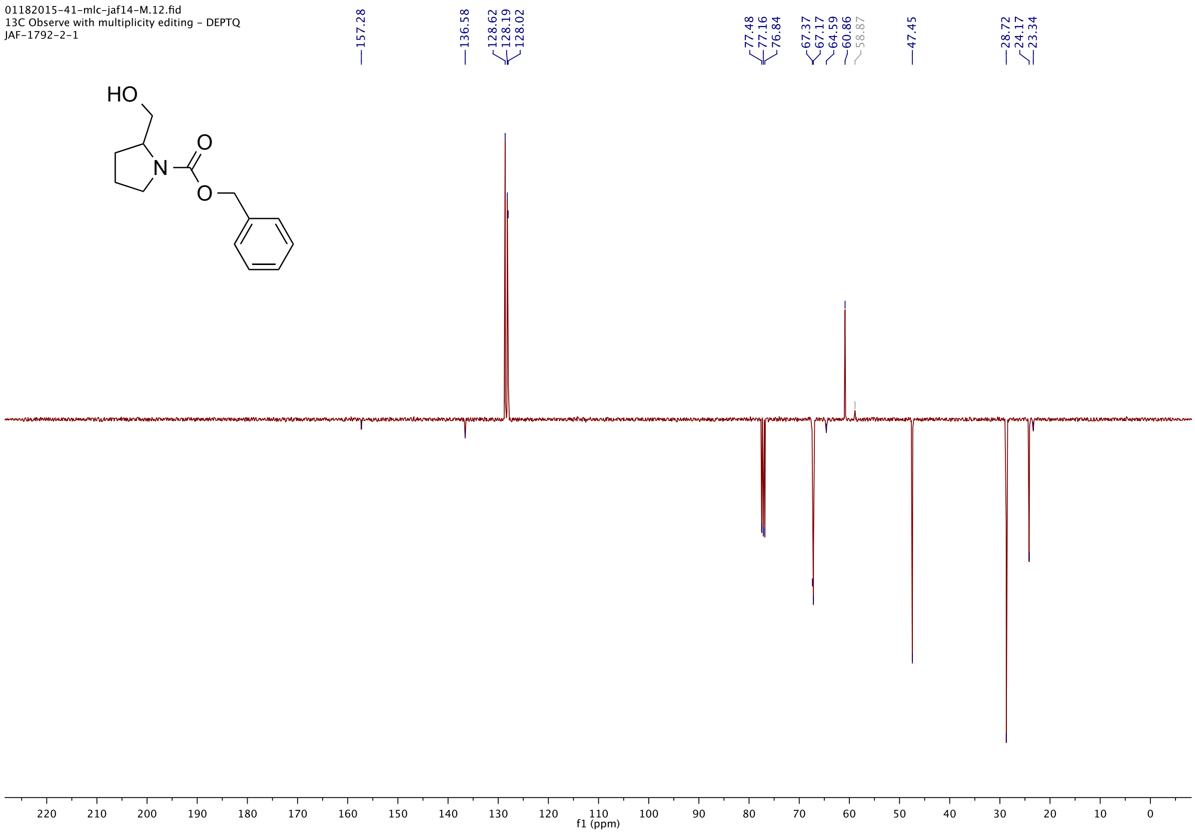

NMR spectra of (1*R*,4*R*,6*R*)-*tert*-butyl 6-formyl-3-oxo-2-azabicyclo[2.2.1]heptane-2-carboxylate and (1*S*,4*S*,5*S*)-*tert*-butyl 5-formyl-3-oxo-2-azabicyclo[2.2.1]heptane-2-carboxylate

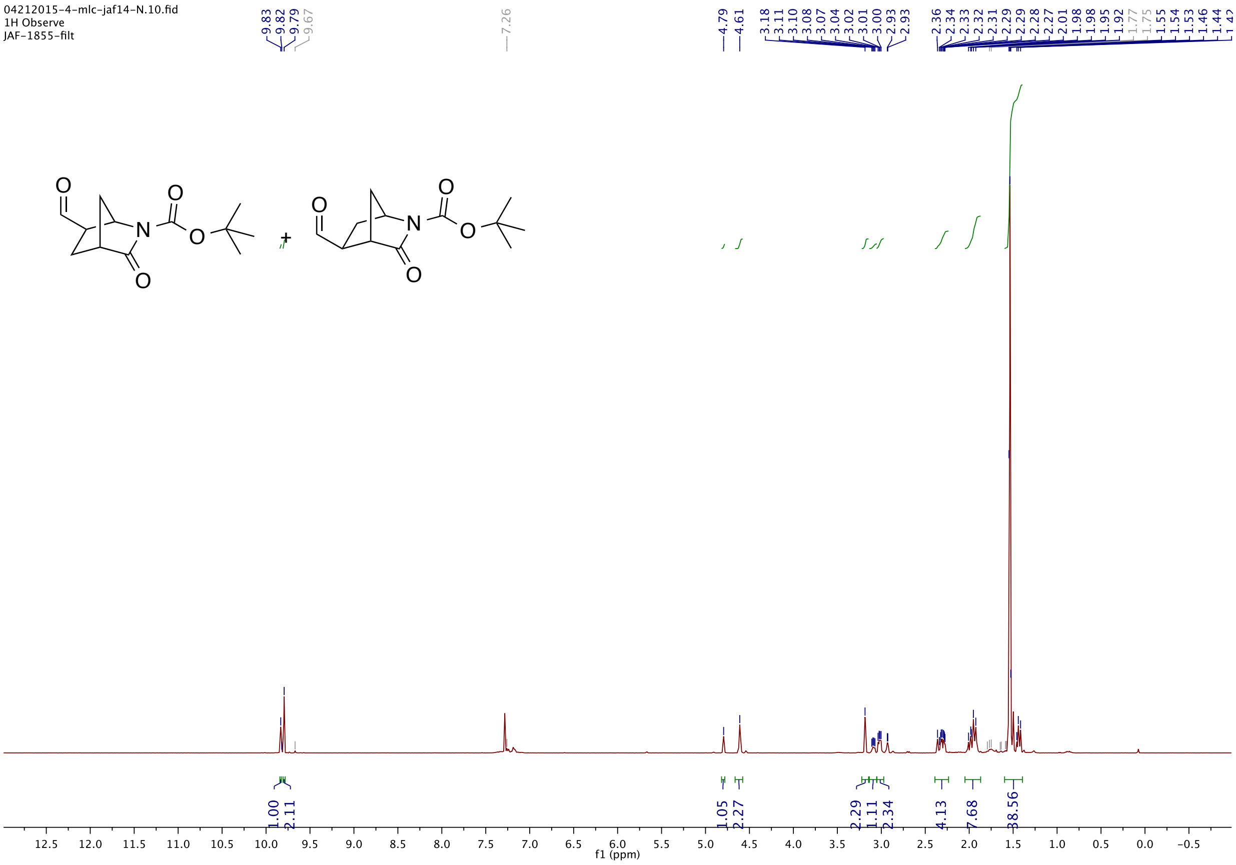

NMR spectra of (1,2-dihydroacenaphthylen-1-yl)methanol

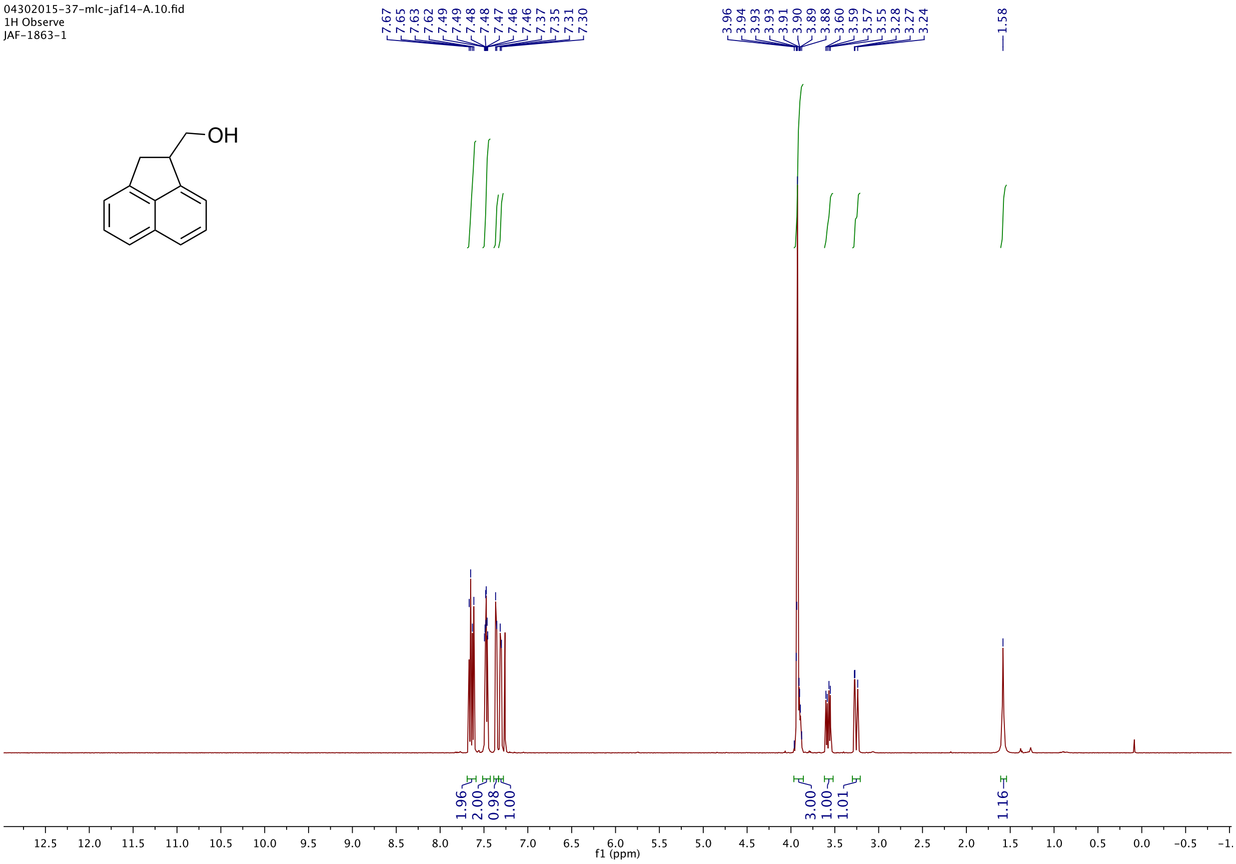

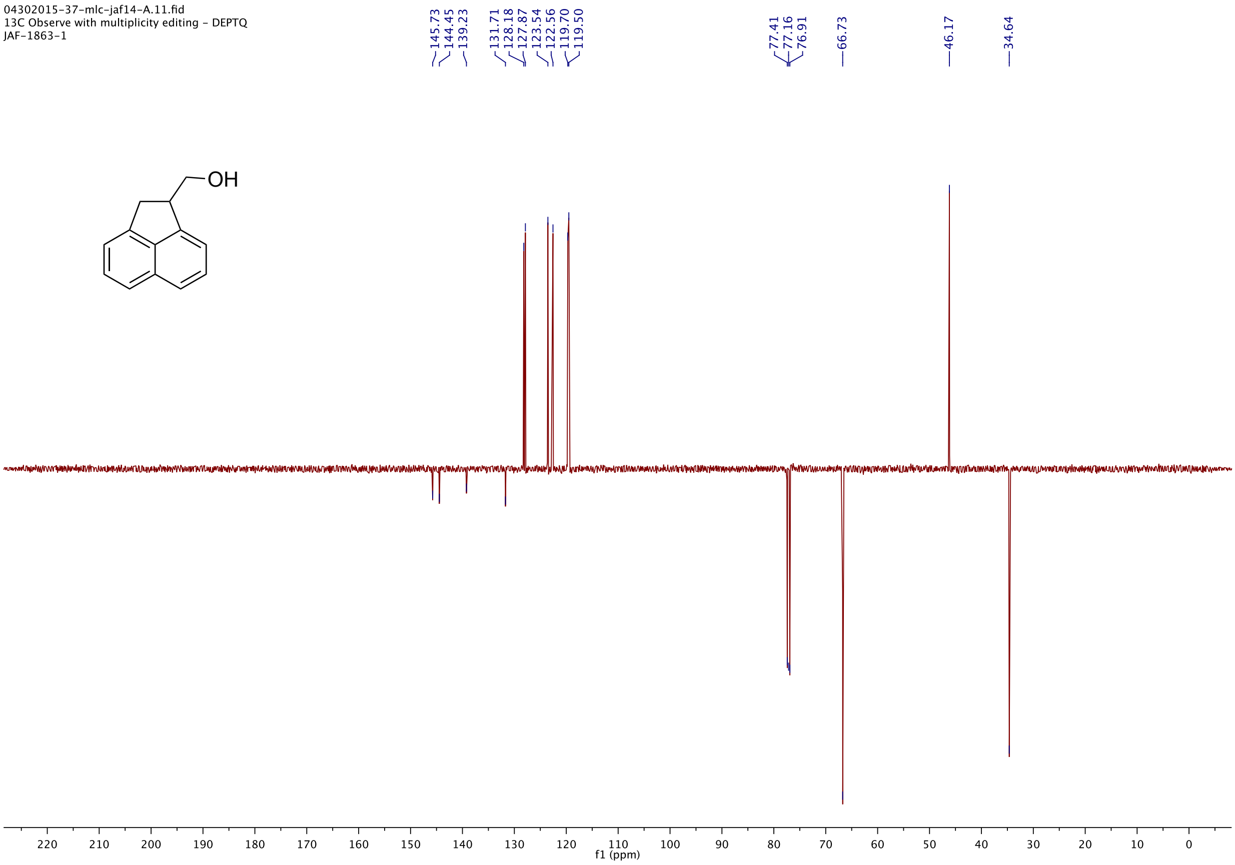

NMR spectra of (1,2-dihydroacenaphthylene-1,1-diyl)dimethanol

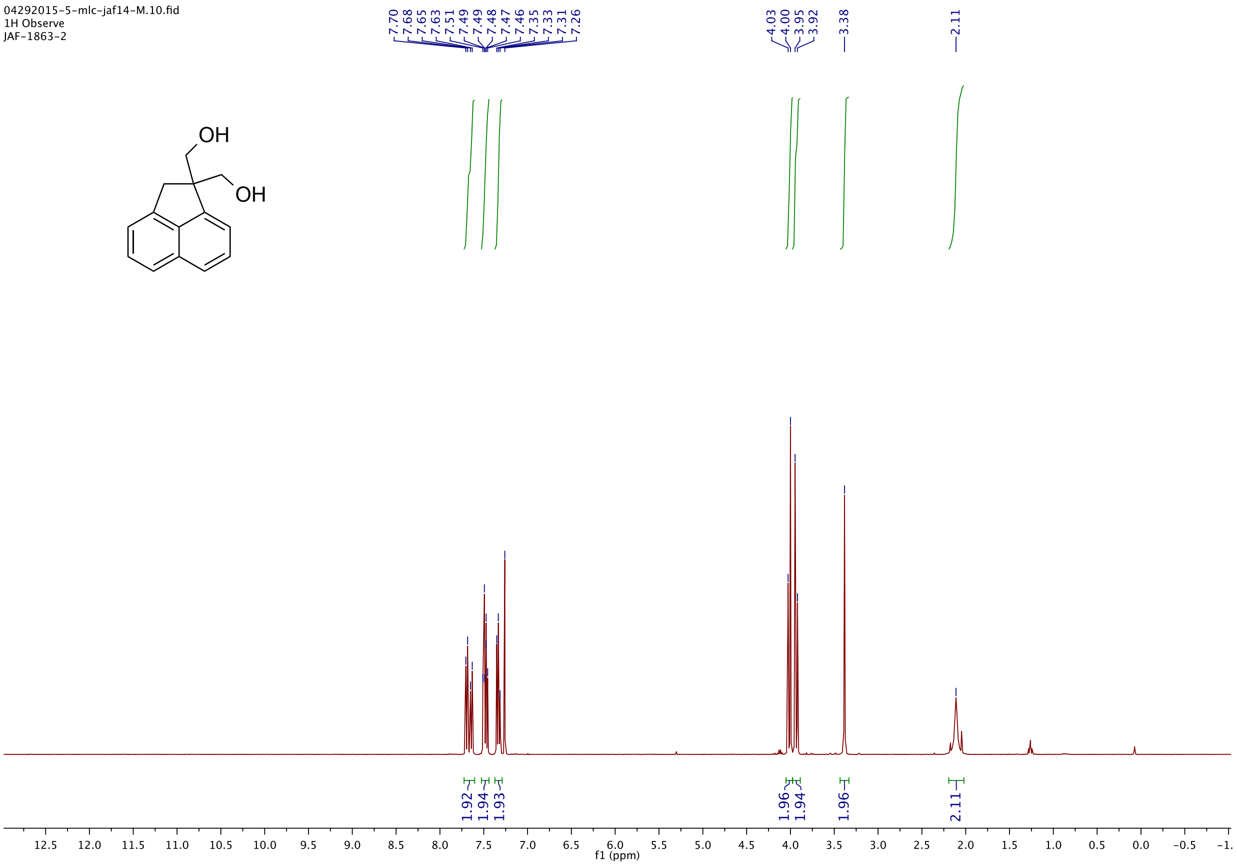

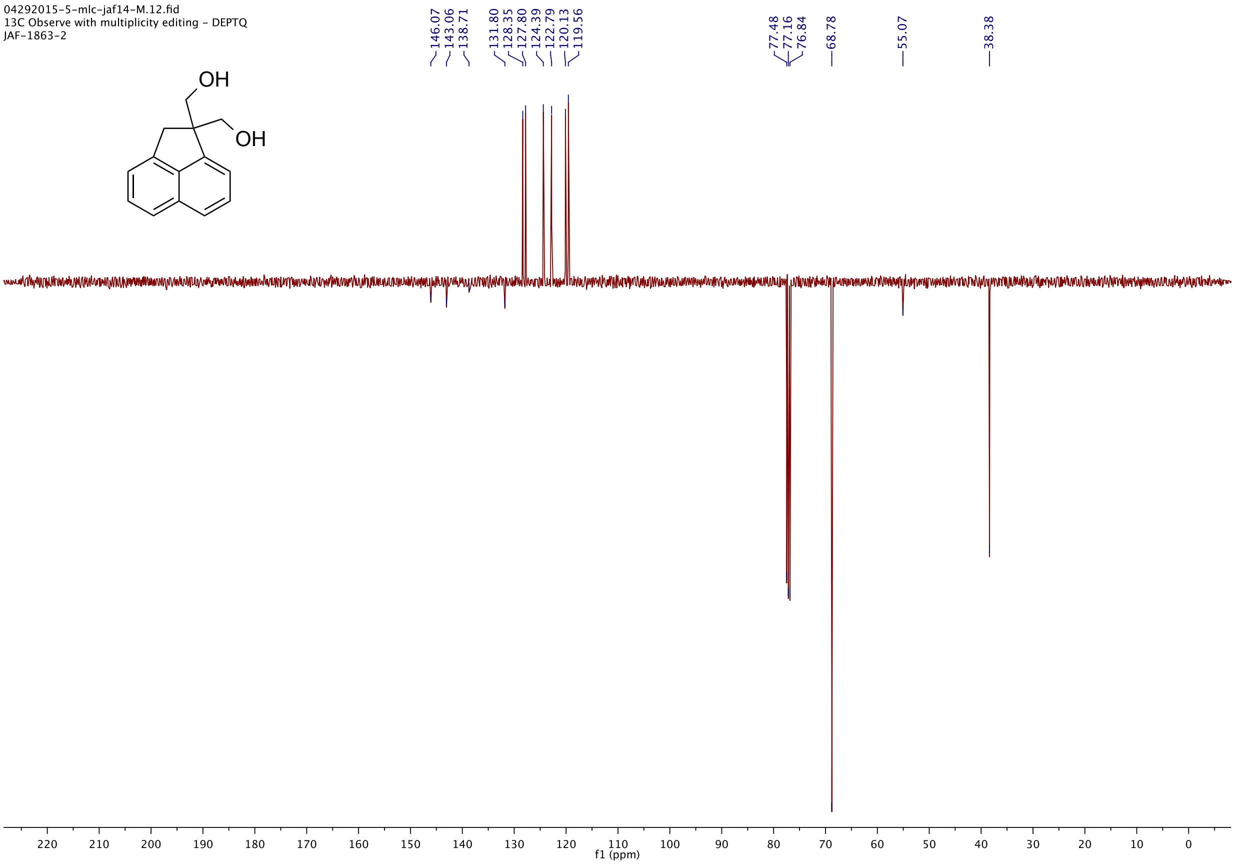

10. HPLC chromatograms for racemic alcohols and catalysis products

2,3-Diphenylpropan-1-ol,

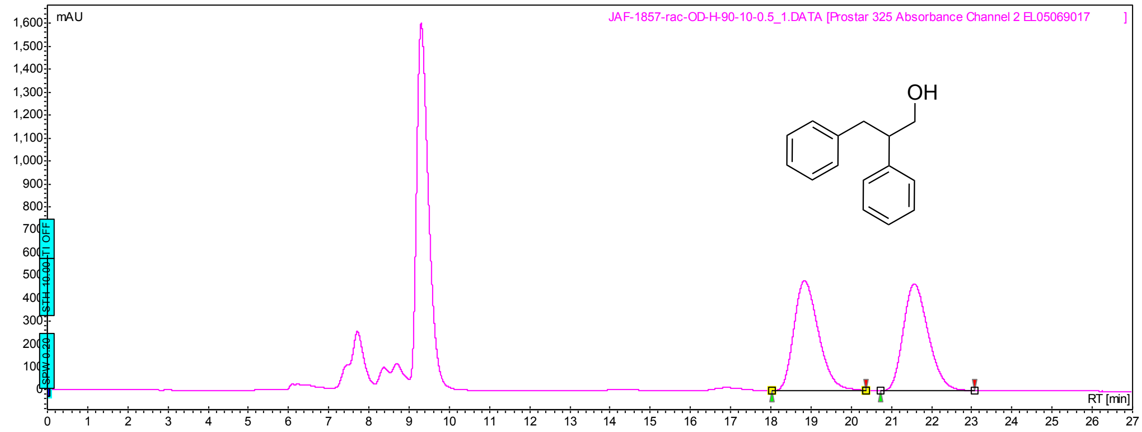

#	Name	Time [Min]	Quantity [% Area]	Height [mAU]	Area [mAU.Min]	Area % [%]
1	UNKNOWN	18.84	50.19	479.6	340.5	50.194
2	UNKNOWN	21.57	49.81	467.3	337.9	49.806
Total			100.00	946.9	678.4	100.000

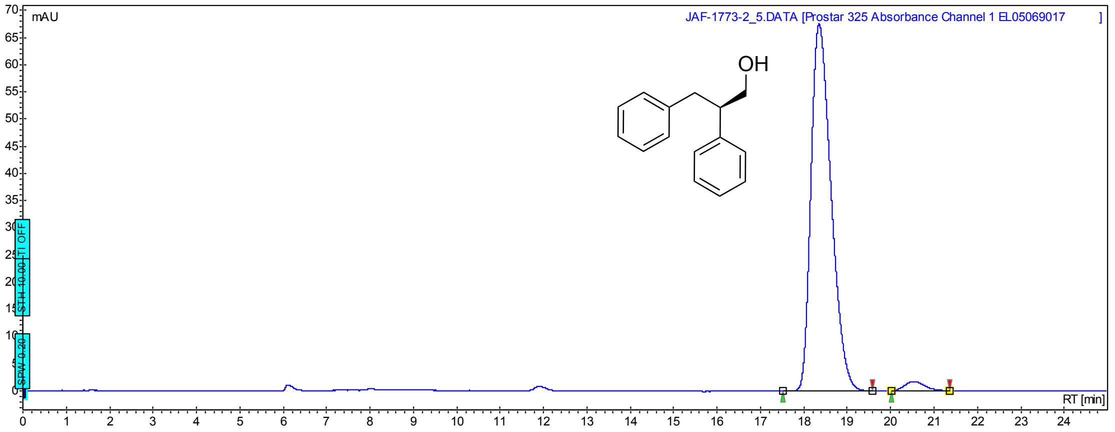

#	Name	Time [Min]	Quantity [% Area]	Height [mAU]	Area [mAU.Min]	Area % [%]
2	UNKNOWN	18.35	97.44	67.5	36.2	97.440
1	UNKNOWN	20.54	2.56	1.8	1.0	2.560
Total			100.00	69.3	37.2	100.000

2,3-Bis(4-methoxyphenyl)propan-1-ol

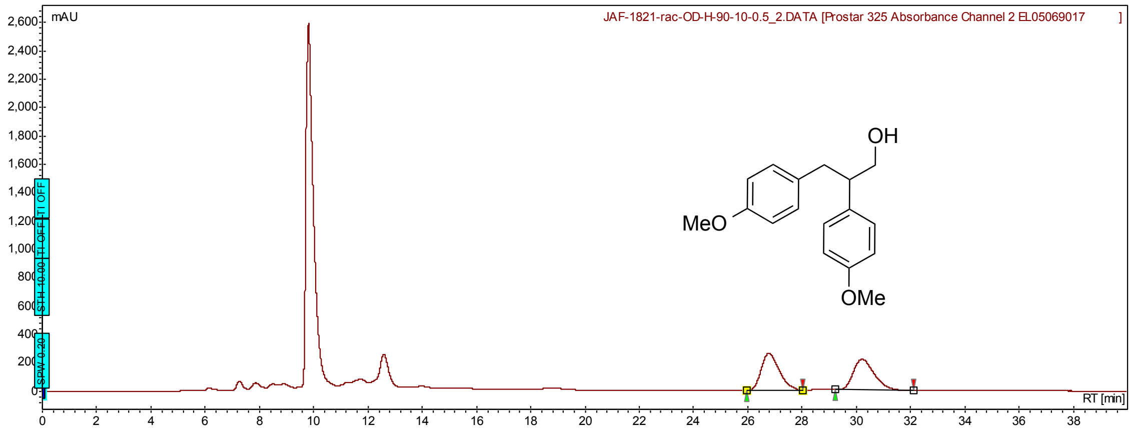

#	Name	Time [Min]	Quantity [% Area]		Height [mAU]	Area [mAU.Min] Area % [%]
1	UNKNOWN	26.78	50.72	259.4	199.9	50.720
2	UNKNOWN	30.21	49.28	217.8	194.2	49.280
Total			100.00	477.3	394.0	100.000

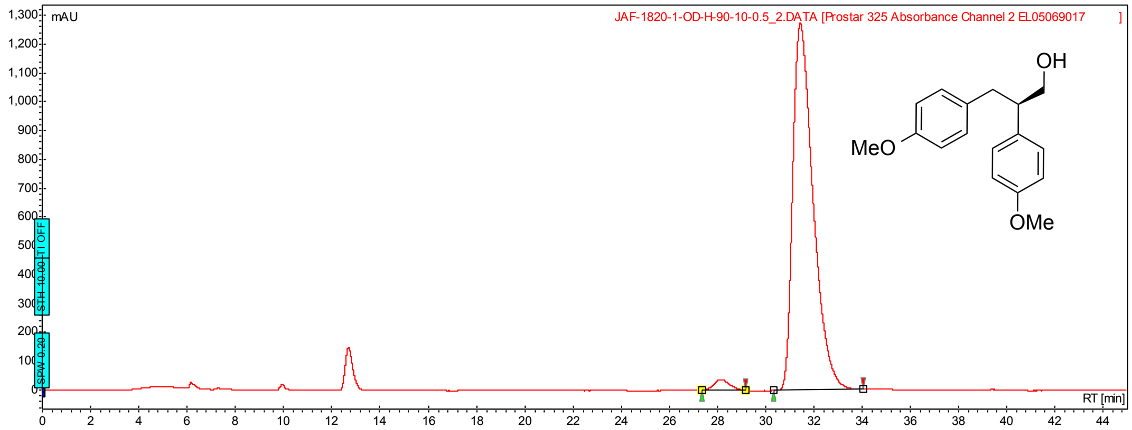

#	Name	Time [Min]	Quantity [% Area]		Height [mAU]	Area [mAU.Min] Area % [%]
1	UNKNOWN	28.14	2.19	36.7	27.9	2.193
2	UNKNOWN	31.42	97.81	1272.5	1243.8	97.807
Total			100.00	1309.2	1271.7	100.000

2,3-Bis(3-methoxyphenyl)propan-1-ol

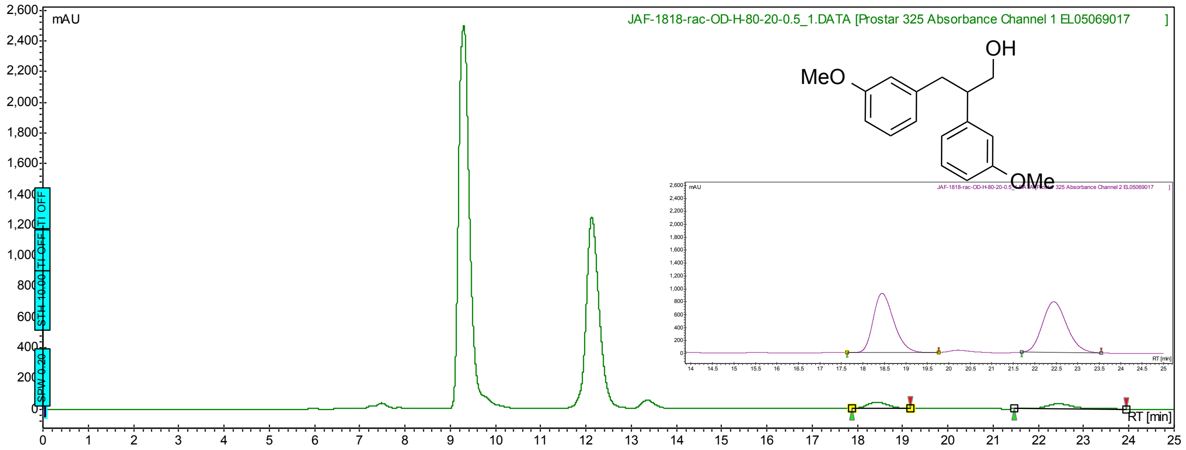

#	Name	Time [Min]	Quantity [% Area]	Height [mAU]	Area [mAU.Min]	Area % [%]
1	UNKNOWN	18.45	49.62	916.5	506.7	49.622
2	UNKNOWN	22.44	50.38	783.9	514.4	50.378
Total			100.00	1700.4	1021.1	100.000

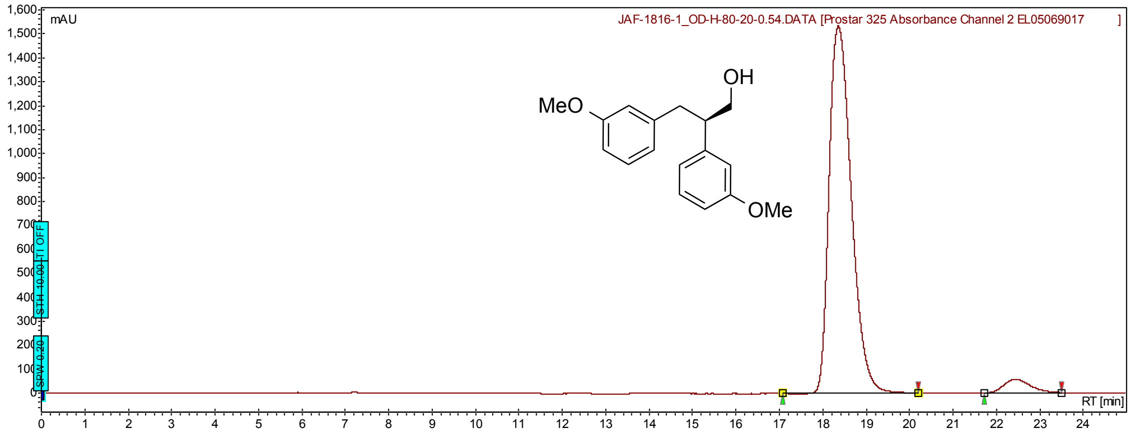

#	Name	Time [Min]	Quantity [% Area]	Height [mAU]	Area [mAU.Min]	Area %
1	UNKNOWN	18.35	95.78	1532.4	907.0	95.776
2	UNKNOWN	22.44	4.22	56.7	40.0	4.224
Total			100.00	1589.2	947.0	100.000

Major isomer: methyl 4-(1-hydroxy-3-(4-methoxyphenyl)propan-2-yl)benzoate (also showing Methyl 4-(3-hydroxy-2-(4-methoxyphenyl)propyl)benzoate)

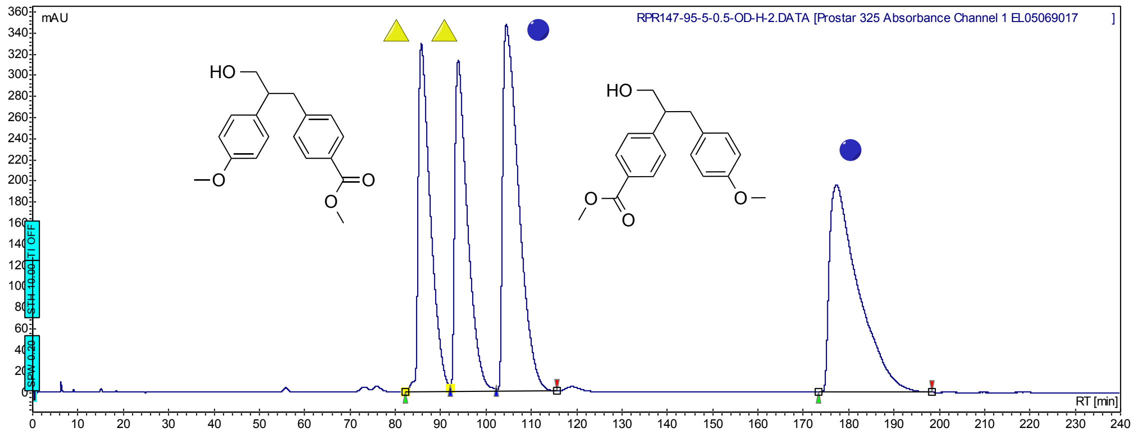

#	Name	Time [Min]	Quantity [% Area]	Height [mAU]	Area [mAU.Min]	Area % [%]
1	UNKNOWN	85.75	20.81	328.9	1027.2	20.810
3	UNKNOWN	93.88	20.71	313.1	1022.4	20.711
4	UNKNOWN	104.48	28.93	346.6	1428.2	28.933
2	UNKNOWN	177.38	29.55	196.6	1458.5	29.546
Total			100.00	1185.3	4936.2	100.000

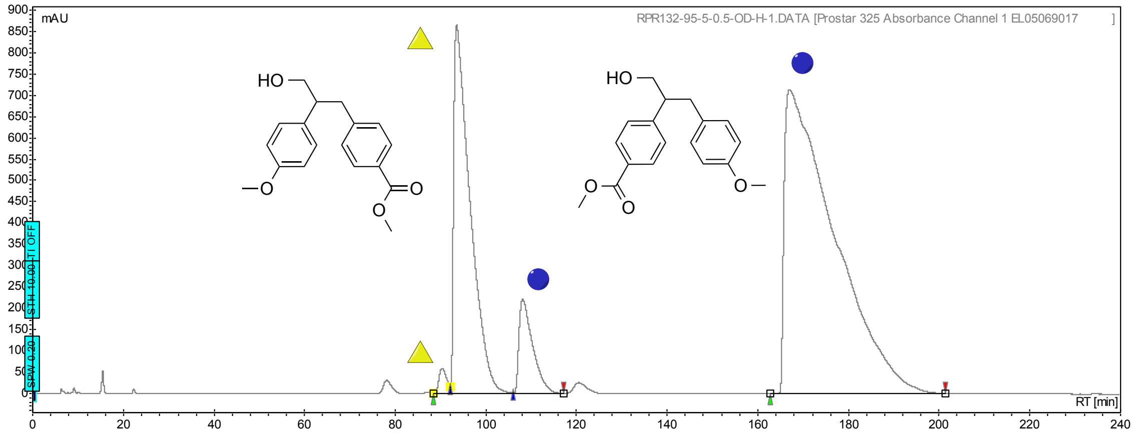

#	Name	Time [Min]	Quantity [% Area]	Height [mAU]	Area [mAU.Min]	Area % [%]
1	UNKNOWN	90.34	0.84	58.0	114.7	0.844
3	UNKNOWN	93.52	26.60	862.8	3614.3	26.602
4	UNKNOWN	108.10	5.70	221.5	773.9	5.696
2	UNKNOWN	166.95	66.86	712.1	9083.4	66.857
Total			100.00	1854.4	13586.3	100.000

(1-tosylpyrrolidin-3-yl)methanol

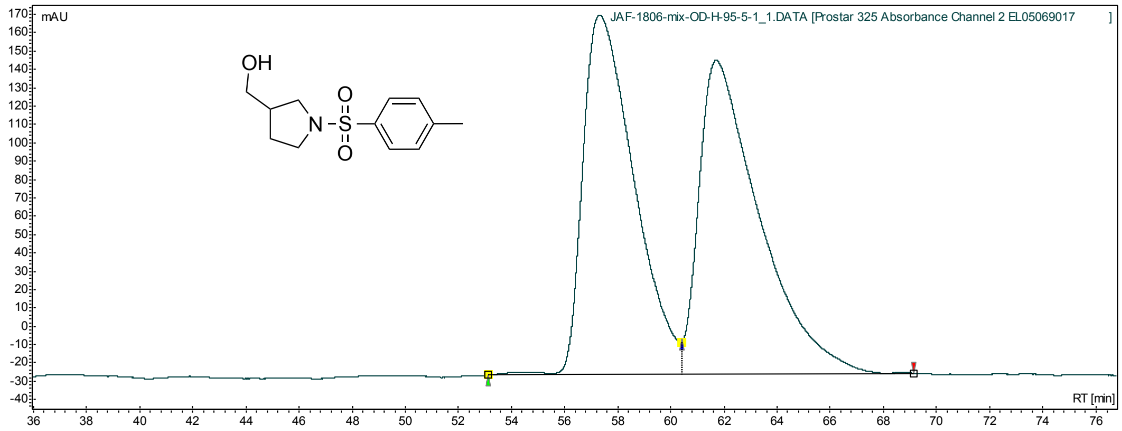

#	Name	Time [Min]	Quantity [% Area]		Height [mAU]	Area [mAU.Min]	Area % [%]
1	UNKNOWN	57.31	49.26	196.0	438.3		49.264
2	UNKNOWN	61.70	50.74	171.1	451.3		50.736
Total			100.0	367.1	889.6		100.000

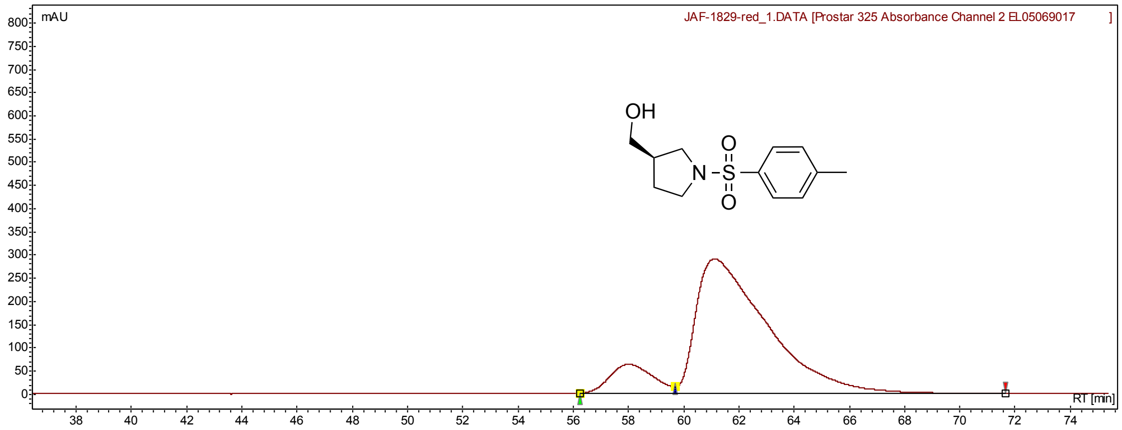

#	Name	Time [Min]	Quantity [% Area]		Height [mAU]	Area [mAU.Min]	Area % [%]
1	UNKNOWN	58.03	11.43	61.6	110.6		11.425
2	UNKNOWN	61.12	88.57	289.3	857.6		88.575
Total			100.00	350.9	968.3		100.000

(1-tosylpyrrolidin-2-yl)methanol

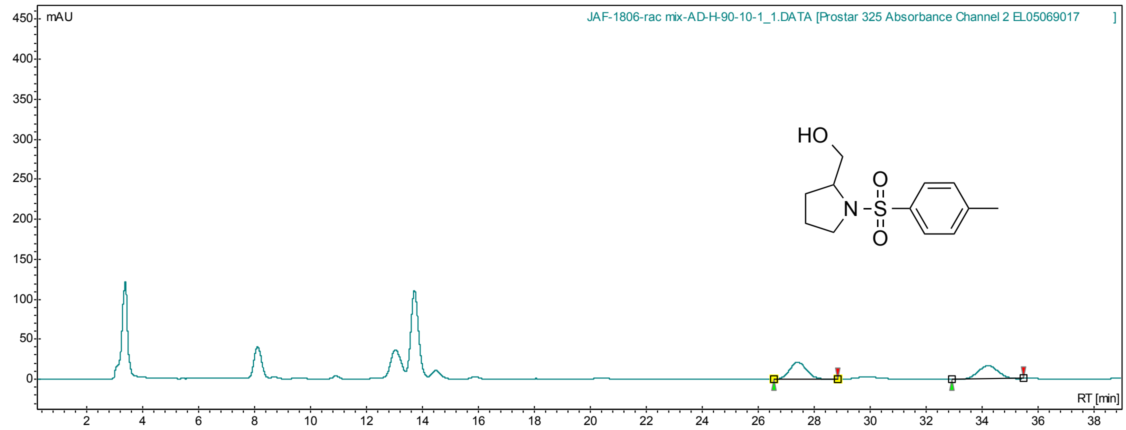

#	Name	Time [Min]	Quantity [% Area]	Height [mAU]	Area [mAU.Min]	Area % [%]
1	UNKNOWN	27.44	49.71	4.7	3.1	49.710
2	UNKNOWN	34.19	50.29	3.7	3.1	50.290
Total			100.00	8.4	6.2	100.000

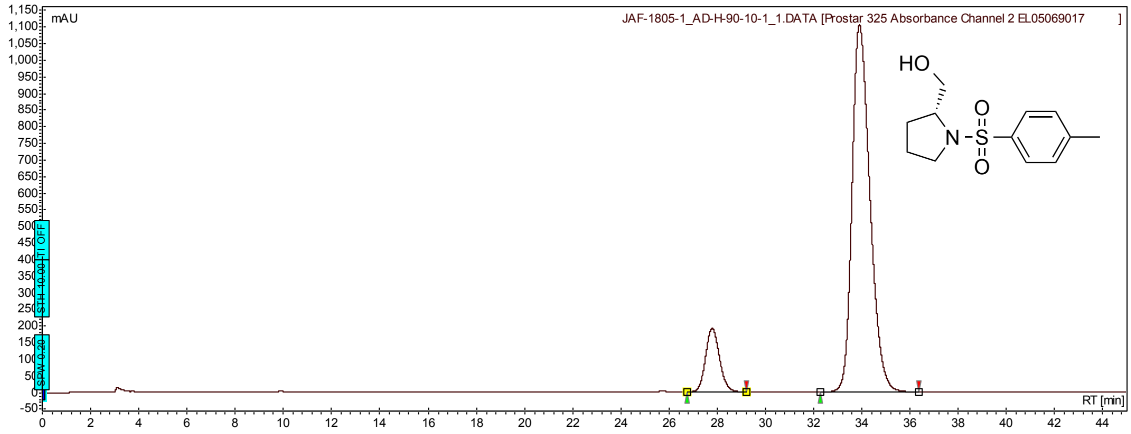

#	Name	Time [Min]	Quantity [% Area]	Height [mAU]	Area [mAU.Min]	Area % [%]
1	UNKNOWN	27.78	11.99	192.1	129.1	11.989
2	UNKNOWN	33.90	88.01	1103.9	947.4	88.011
Total			100.00	1296.0	1076.5	100.000

(1,2-dihydroacenaphthylen-1-yl)methanol

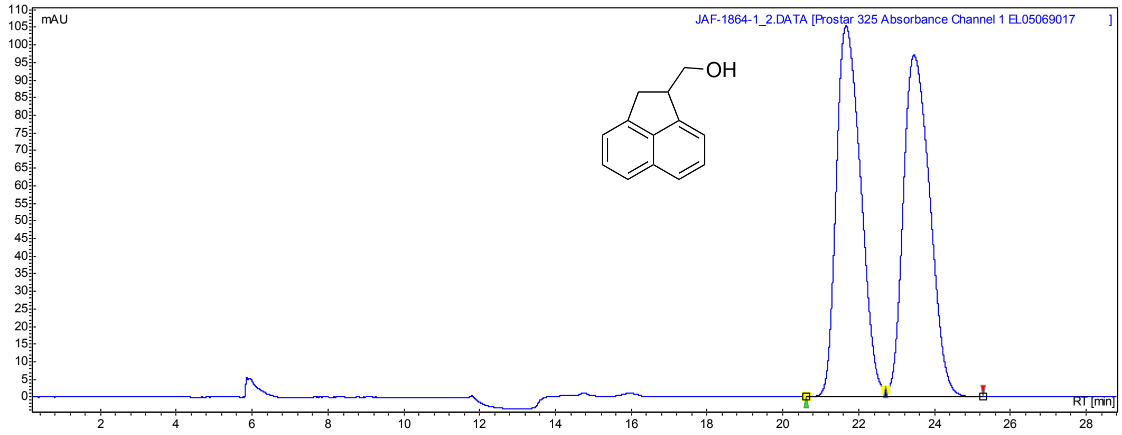

#	Name	Time [Min]	Quantity [% Area]	Height [mAU]	Area [mAU.Min]	Area % [%]
1	UNKNOWN	21.66	49.90	105.3	78.4	49.901
2	UNKNOWN	23.46	50.10	97.0	78.7	50.099
Total			100.00	202.2	157.1	100.000

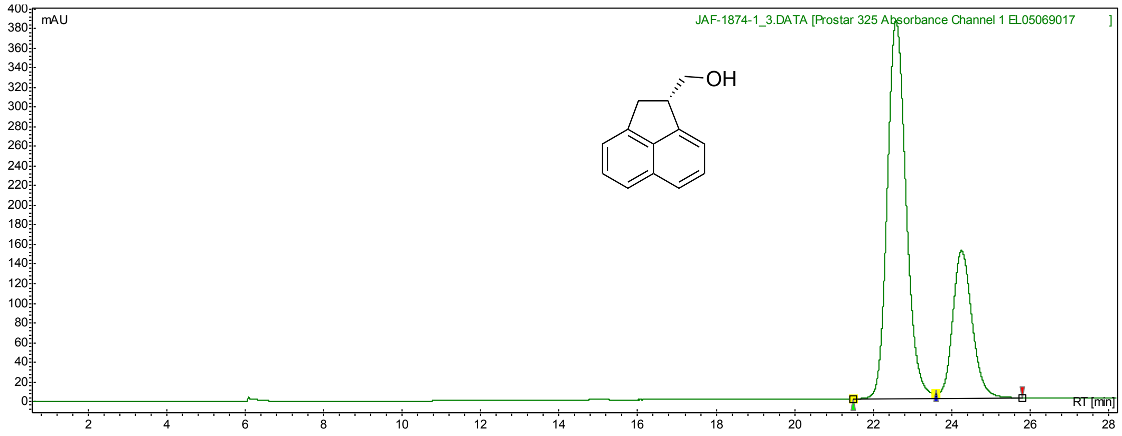

#	Name	Time [Min]	Quantity [% Area]	Height [mAU]	Area [mAU.Min]	Area % [%]
1	UNKNOWN	22.59	71.18	385.2	213.6	71.176
2	UNKNOWN	24.26	28.82	150.4	86.5	28.824
Total			100.00	535.7	300.1	100.000

11. References

1. R. A. Baber, M. L. Clarke, K. M. Heslop, A. C. Marr, A. G. Orpen, P. G. Pringle, A. Ward and D. E. Zambrano-Williams, *Dalton Trans.*, **2005**, 1079.

2. Y. Shi, X. Li, J. Liu, W. Jiang, L. Sun, Appl. *Organomet. Chem.* **2011**, *25*, 514.

3. S. Trosien, S. R. Waldvogel, *Org. Lett.* **2012**,14, 2976.

4. Y. Liu, L. Hu, H. Chen, H. Du, *Chem. Eur. J.* **2015**, 21, 3495.

5. F. Lara-Ochoa, G. Espinosa-Perez, *Tetrahedron Lett.* **2007**, *48*, 7007.

6. Y-J. Chen, C. Chen, *Tetrahedron Asymmetry*, **2007** 18, 1313.

7.F. Alonso, P. Riente, M. Yus, *Eur. J. Org. Chem.* **2009**, *34*, 6034.

8. A. L. Watkins, B. G. Hashiguchi, C. R. Landis, *Org. Lett.* **2008**, *10*, 4553.

9. R. Cano, M. Yus, D. J. Ramon, *Chem. Commun.* **2012**, *48*, 7628.

10. D. Rodriguez-Lucena, M. S. T. Morin, P. Compain, *Letters in Organic Chemistry*, **2011**, *8*, 155.

11. P. H. Fuller, J-W. Kim, S. R. Chemler. *J. Am. Chem. Soc.* **2008**, *130*, 17638.

12. G. M. Noonan, C. J. Cobley, T. Lebl, M. L. Clarke. *Chem. Eur. J.* **2010**, *16*, 12788.

13. E. Zuidema, L. Eascorihuela, T. Eichelsheim, J. J. Carbó, C. Bo, P. C. J. Kamer, P. W. N. M. van Leeuwen, *Chem. Eur. J.* **2008**, *14*, 1843.

14. J. Gu, T. Storz, F. Vyverberg, C. Wu, R. J. Varsolona, K. Sutherland. *Org. Process Res. Dev.* **2011**, *15*, 942.

15. N. Haddad, E. Abu-Shqara, *J. Org. Chem.* **1994**, *59*, 6090.

16. L.L.J.M. Cornelissen PhD thesis, Technical University of Eindhoven, (Prof D. Vogt research group), available from: http://repository.tue.nl/643446
